# A Functional Switch of NuRD Chromatin Remodeling Complex Subunits Regulates Mouse Cortical Development

**DOI:** 10.1016/j.celrep.2016.10.022

**Published:** 2016-11-01

**Authors:** Justyna Nitarska, Jacob G. Smith, William T. Sherlock, Michele M.G. Hillege, Alexi Nott, William D. Barshop, Ajay A. Vashisht, James A. Wohlschlegel, Richard Mitter, Antonella Riccio

**Affiliations:** 1 MRC Laboratory for Molecular and Cell Biology, University College London, London WC1E 6BT, UK; 2 Department of Biological Chemistry, David Geffen School of Medicine at UCLA, Los Angeles, CA 90095-1737 USA; 3 Lincoln’s Inn Fields Laboratory, The Francis Crick Institute, London WC2A 3LY, UK

**Keywords:** epigenetics, chromatin remodeling, CHD proteins, NuRD complex, mouse brain, cortical development, neural radial migration, neural progenitors, cortical laminar fate specification

## Abstract

Histone modifications and chromatin remodeling represent universal mechanisms by which cells adapt their transcriptional response to rapidly changing environmental conditions. Extensive chromatin remodeling takes place during neuronal development, allowing the transition of pluripotent cells into differentiated neurons. Here, we report that the NuRD complex, which couples ATP-dependent chromatin remodeling with histone deacetylase activity, regulates mouse brain development. Subunit exchange of CHDs, the core ATPase subunits of the NuRD complex, is required for distinct aspects of cortical development. Whereas CHD4 promotes the early proliferation of progenitors, CHD5 facilitates neuronal migration and CHD3 ensures proper layer specification. Inhibition of each CHD leads to defects of neuronal differentiation and migration, which cannot be rescued by expressing heterologous CHDs. Finally, we demonstrate that NuRD complexes containing specific CHDs are recruited to regulatory elements and modulate the expression of genes essential for brain development.

## Highlights

• The ATPases CHD3, CHD4, and CHD5 are mutually exclusive subunits of the NuRD complex
• CHD3, CHD4, and CHD5 regulate distinct and non-redundant aspects of cortical development
• Loss of each CHD leads to specific defects of neuronal proliferation and migration
• CHD3, CHD4, and CHD5 regulate distinct set of genes essential for brain development

## Graphical Abstract

**Figure:**
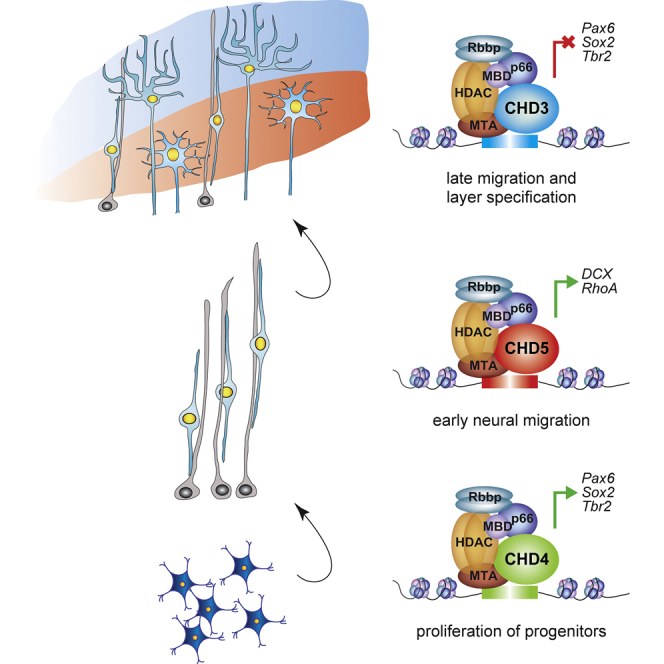

## Teaser

Neural development requires active chromatin remodeling. Nitarska et al. identify distinct NuRD chromatin remodeling complexes, containing CHD3, CHD4, or CHD5, during mouse embryonic cortical development. CHD4 promotes proliferation of basal progenitors, while CHD5 facilitates early radial migration and CHD3 drives late migration and laminar specification of neurons.

## Introduction

The ability to adjust the transcriptional output in response to ever-changing environmental conditions lies at the core of organismal development. The complex cytoarchitecture of the mammalian cortex provides a superb example of how extracellular and intracellular stimuli cooperate to transform a pool of undifferentiated neural progenitor cells (NPCs) into a highly organized layered tissue. Each cortical layer contains morphologically and functionally distinct subsets of neurons (Kwan et al., 2012) that derive from multipotent NPCs generated in the ventricular zone (VZ) of the embryonic brain. All developmental steps that lead to the formation of the mature cortex depend on the expression of specific genes that are necessary for NPC proliferation and, at later stages, neuronal migration and laminar specification.

Epigenetic modifications and changes of chromatin structure are emerging as fundamental mechanisms that regulate gene expression during brain development (Hirabayashi and Gotoh, 2010, Riccio, 2010). Chromatin is a highly dynamic structure that can be modified by a number of mechanisms, including DNA methylation, histone post-translational modifications, and ATP-dependent remodeling of chromatin (Borrelli et al., 2008). The latter mechanism uses energy released by hydrolysis of ATP to induce nucleosome sliding, facilitating the recruitment of transcriptional complexes (Narlikar et al., 2013) that activate or inhibit gene expression.

The nucleosome remodeling and deacetylase (NuRD) complex couples ATP-dependent chromatin remodeling with histone deacetylase activity (Tong et al., 1998, Xue et al., 1998, Zhang et al., 1998). NuRD is composed of six core subunits, each encoded by homologous gene families (Bowen et al., 2004). The ATPase activity of NuRD is provided by the chromodomain-helicase DNA-binding proteins (CHDs) 3, 4, or 5, and deacetylase activity by HDAC1 or HDAC2. Additional core subunits include methyl-CpG-binding domain proteins (MBDs) 1, 2, or 3; metastasis-associated proteins (MTAs) 1, 2, or 3; the histone-binding proteins Rbbp4 or Rbbp7; and the nuclear zinc-finger proteins Gata2a or Gata2b (Basta and Rauchman, 2015, Lai and Wade, 2011). NuRD has been linked to a number of basic cellular functions that take place during development, including the maintenance of genome integrity and cell-cycle progression (Lai and Wade, 2011). Recent studies have also linked NuRD to regulation of gene expression during embryonic stem cell (ESC) differentiation and lineage commitment of pluripotent cells (Kashiwagi et al., 2007, Reynolds et al., 2012a, Zhang et al., 2011). While NuRD has been implicated in the transcriptional repression of Polycomb-regulated genes in ESCs (Reynolds et al., 2012b) and in differentiated neurons (Egan et al., 2013), the role of NuRD in the developing nervous system remains largely unknown.

Here, we show that NuRD represents a major chromatin remodeling complex in the developing mouse cortex. We found that a sequential switch of CHDs within the complex results in the combinatorial assembly of NuRD complexes that regulate the transcription of genes necessary for neural progenitor proliferation, radial migration, and cortical layer specification.

## Results

### NuRD Is the Major Nuclear Complex Associated with HDAC2 in the Developing Cortex

**Figure 1:**
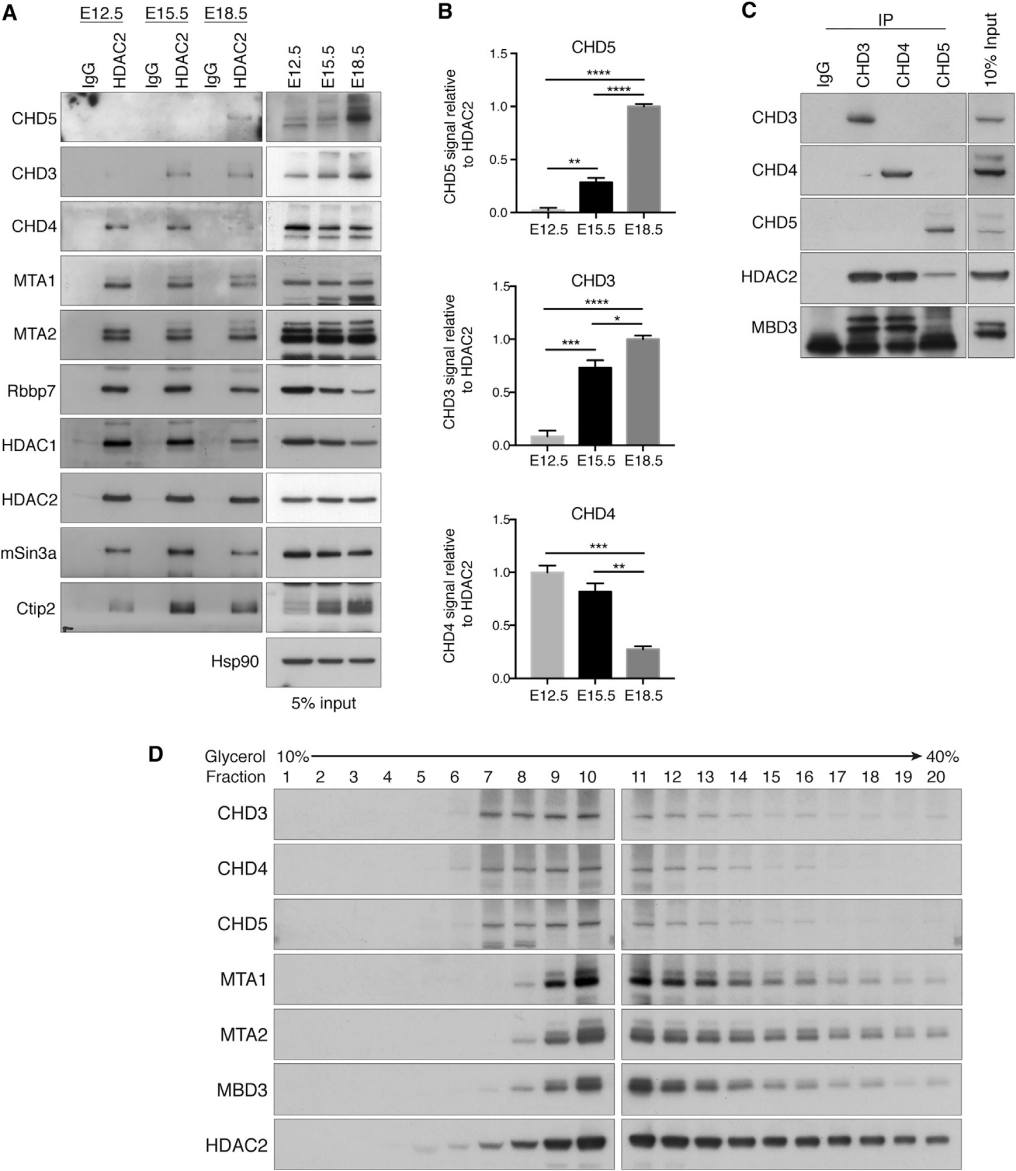
Characterization of NuRD Complexes in the Developing Cortex (A) Lysates of cortices were subjected to co-immunoprecipitation with HDAC2 antibody followed by immunoblotting for NuRD subunits. Representative western blot; n = 3. (B) Densitometry analysis of CHDs co-immunoprecipitating with HDAC2 in E12.5, E15.5, and E18.5 cortex. Data are presented as mean ± SEM of three independent experiments. ^∗^p < 0.05, ^∗∗^p < 0.01, ^∗∗∗^p < 0.001, and ^∗∗∗∗^p < 0.0001 by one way ANOVA with Tukey’s multiple comparisons test. (C) Lysates of E15.5 cortices were subjected to co-immunoprecipitation with CHD3, CHD4 or CHD5 antibodies followed by immunoblotting for HDAC2 and MBD3. Representative blot; n = 3. (D) Glycerol gradient co-sedimentation analysis of nuclear extracts from E15.5 cortices. Representative blot; n = 3. See also Figures S1–S4.

Recent work from our lab (Nott et al., 2008, Nott et al., 2013) and others (Hagelkruys et al., 2014, Hagelkruys et al., 2015) demonstrated that the class I histone deacetylases HDAC1 and HDAC2 control the expression of genes necessary for neuronal development. Class I HDACs do not bind DNA directly and are found within large multiprotein complexes that confer target specificity (Haberland et al., 2009). To identify HDAC2-containing complexes in the embryonic mouse brain, we performed mass spectrometry of proteins that co-immunoprecipitate with HDAC2 during cortical development. Cortices were dissected at embryonic day 12.5 (E12.5), E15.5, and E18.5, and cell lysates were incubated with HDAC2 antibody or normal immunoglobulin G (IgG). Immunoprecipitated proteins were proteolytically digested and analyzed by mass spectrometry (Figure S1A). The relative abundance of identified proteins was calculated using normalized spectra abundance factors (NSAFs). Proteins enriched in HDAC2 immunoprecipitates are listed in Figure S1B and Table S1. Strikingly, all known subunits of the NuRD complex were associated with HDAC2 at all embryonic stages (Figure S1B). Subunits of the CoREST (REST corepressor 1) and mSin3 complexes were also detected (Figure S1B). HDAC2 co-immunoprecipitation followed by western blot analysis of CHD3, CHD4, CHD5, MTA1, MTA2, and Rbbp7 confirmed the findings of mass spectrometry analysis and revealed that the association of CHDs with HDAC2 changed according to developmental stage (Figures 1A and 1B). While CHD4 co-immunoprecipitated with HDAC2 at E12.5 and E15.5, CHD3 and CHD5 were not detected at E12.5 and became part of the NuRD complex at later stages of cortical development. CHD3 co-immunoprecipitated with HDAC2 at both E15.5 and E18.5, whereas CHD5 was detected predominantly at E18.5. Importantly, co-immunoprecipitation experiments confirmed mutually exclusive occupancy of CHD3, CHD4, and CHD5 within NuRD complexes (Figure 1C). Glycerol gradient analysis performed on E15.5 cortices indicated that 78% of CHDs co-sediment as a complex in fractions that also contain the NuRD subunits MTA1, MTA2, and Mbd3 (Figures 1D and S1C). Taken together, these results indicate that the composition of NuRD complex changes during cortical development and that CHD3, CHD4, and CHD5 occupancy within the complex is mutually exclusive.

### CHDs Are Developmentally Regulated in the Cortex

The mouse cortex is formed between E11 and E18 in a characteristic inside-out manner, with deep layers (layers IV–VI) generated first and more superficial layers (layers II and III) generated later (Florio and Huttner, 2014). Neurons that will eventually occupy the external layers of the cortex must migrate through the deeper layers in order to reach their final location. During migration and laminar formation, neurons mostly maintain the molecular properties acquired at early stages, although their laminar identity is further specified by postmitotic factors that contribute to the establishment of mature neuronal identity (Kwan et al., 2012). To investigate the expression of CHDs at different stages of cortical development, mouse cortices were dissected at E12.5, E15.5, and E18.5 and subjected to qRT-PCR and western blot analyses (Figures S2A and S2B). At E12.5, CHD3 and CHD5 were expressed at relatively low levels that increased at E15.5 and E18.5. In contrast, CHD4 expression remained constant throughout development. Immunostaining of coronal sections confirmed that CHD4 was the only CHD detected at E12.5, with strong expression observed in NPCs (Figure S2C). At later stages (E15.5 and E18.5), CHD3 and CHD5 were also expressed and found in differentiated neurons of the nascent cortical plate (CP), where they co-localized with the deep layer neuronal marker Ctip2 (Figures S2C and S3). A similar expression pattern was observed when neural progenitors isolated from E14.5 rat cortices were differentiated in vitro and immunostained for CHDs and either nestin, a marker of NPCs, or the neuronal marker MAP2 (Figures S4A and S4B). Co-immunoprecipitation experiments confirmed that HDAC2 and the core NuRD subunit MTA2 mostly interact with CHD4 in NPCs, whereas differentiation correlates with a decline in CHD4 interaction and association of both CHD3 and CHD5 (Figures S4C and S4D). Thus, the ATPase activity of NuRD is mostly mediated by CHD4 in NPCs, whereas this function is largely provided by CHD3 and CHD5 at later differentiation stages. Given that the occupancy of CHDs within the NuRD complex is mutually exclusive (Figure 1C), a possible interpretation of these findings is that the coexistence of distinct NuRD complexes within neurons may be necessary to regulate diverse molecular functions.

### Deletion of CHD4 Causes Premature Cell-Cycle Exit of NPCs and Depletion of Intermediate Progenitor Cells

**Figure 2:**
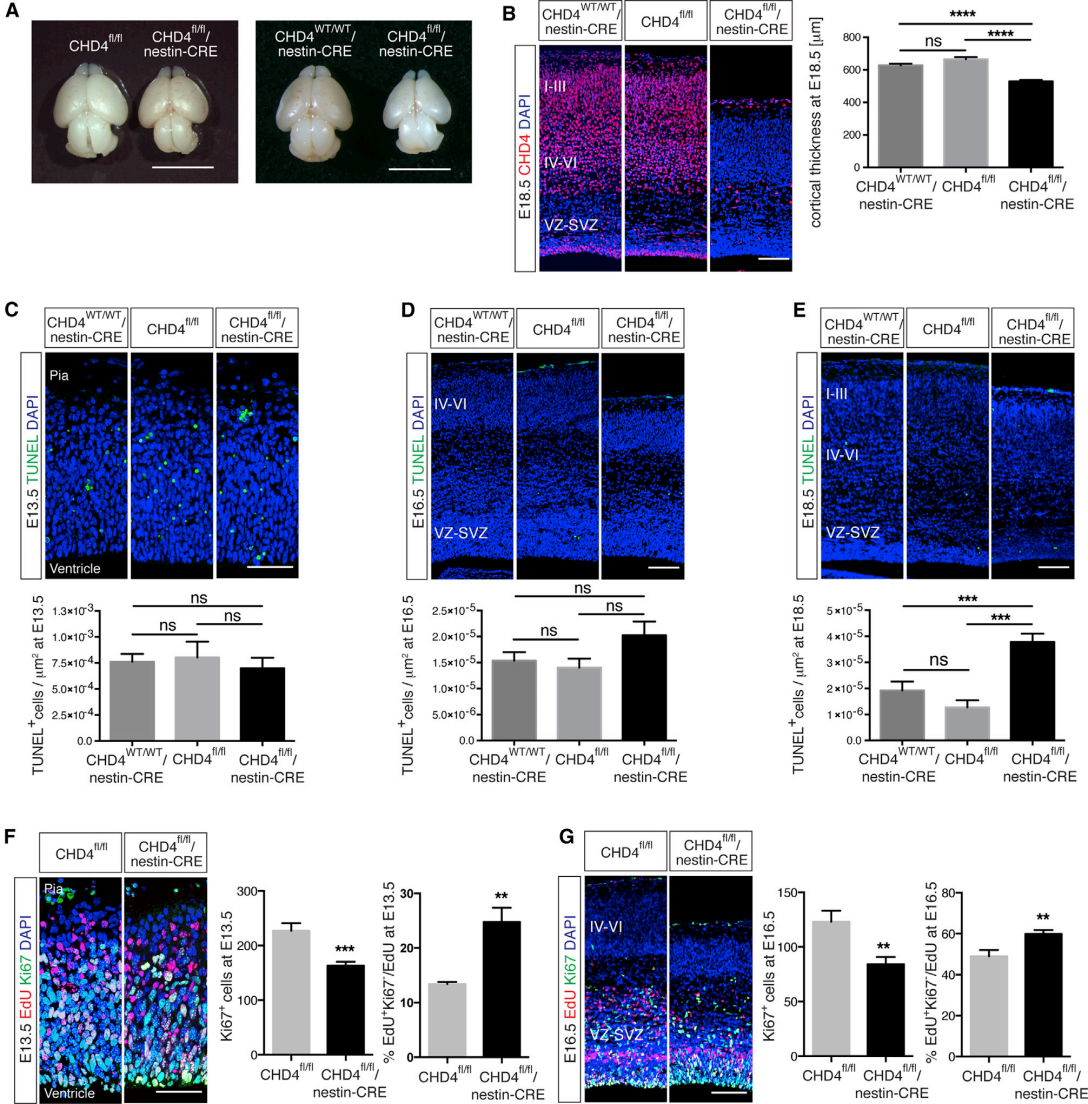
Deletion of CHD4 Causes Microcephaly and Premature Cell-Cycle Exit of NPCs (A) CHD4^fl/fl^/nestin-CRE and control (CHD4^fl/fl^ and CHD4^WT/WT^/nestin-CRE) brains dissected at E18.5. Scale bar, 5 mm. (B) Left: coronal sections of CHD4^fl/fl^/nestin-CRE and control brains at E18.5 immunolabeled for CHD4 (red). Scale bar, 100 μm. Right: cortical thickness in E18.5 embryos, measured across the cortical wall using coronal sections; five to ten embryos obtained from four litters were analyzed per genotype. (C) Top: TUNEL staining of CHD4^fl/fl^/nestin-CRE cortex at E13.5. Scale bar, 50 μm. Bottom: number of TUNEL-positive cells per 1 μm^2^ of cortical area. Four to nine embryos obtained from four litters were analyzed per genotype. (D) Top: TUNEL analysis of CHD4^WT/WT^/nestin-CRE, CHD4^fl/fl^ and CHD4^fl/fl^/nestin-CRE cortices at E16.5. Scale bar, 100 μm. Bottom: number of TUNEL-positive cells per 1 μm^2^ of cortical area. 5–12 embryos obtained from five litters were analyzed per genotype. (E) Top: TUNEL analysis of CHD4^fl/fl^/nestin-CRE cortex at E18.5. Scale bar, 100 μm. Bottom: number of TUNEL-positive cells per 1 μm^2^ of cortical area. Five to ten embryos obtained from four litters were analyzed per genotype. (F) Analysis of cell-cycle exit index of CHD4^fl/fl^/nestin-CRE cortices at E13.5. EdU in vivo labeling at E12.5 followed by analysis of Ki67 expression at E13.5. 5–11 embryos obtained from five litters were analyzed per genotype. Scale bar, 50 μm. (G) Analysis of Ki67-positive cells and cell-cycle exit index of the CHD4^fl/fl^/nestin-CRE cortex at E16.5 performed as in (F). Scale bar, 100 μm. All data are presented as mean ± SEM. ^∗∗^p < 0.01 and ^∗∗∗^p < 0.001 (ns, not significant) by one-way ANOVA with Tukey’s multiple comparisons test (B–E) or unpaired t test (F and G).

To investigate the role of CHDs during cortical development, we first analyzed the cortex of mice carrying a conditional mutation of the CHD4 gene (Williams et al., 2004). CHD4 floxed mice were crossed with transgenic mice carrying CRE recombinase under the control of the nestin promoter, which results in deletion of CHD4 in the nervous system from early embryonic stages (Dubois et al., 2006). Most mice lacking CHD4 (CHD4^fl/fl^/nestin-CRE) died at birth and at E18.5 the brain was significantly smaller than control littermates (CHD4^fl/fl^ and CHD4^WT/WT^/nestin-CRE; Figure 2A), showing remarkable reduction of cortical thickness (Figure 2B). Although TUNEL staining performed at both E13.5 and E16.5 showed no increase in cell death in CHD4^fl/fl^/nestin-CRE cortices compared to control littermates (Figures 2C and 2D), we observed a reduction of the number of cells expressing the proliferation marker Ki67 (Figures 2F and 2G). EdU (5-ethynyl-2’-deoxyuridine) labeling coupled with Ki-67 immunostaining revealed that in CHD4^fl/fl^/nestin-CRE cortices, NPCs prematurely exited the cell cycle (Figures 2F and 2G), and this event was associated with increased apoptosis at E18.5 (Figure 2E). Thus, in cortices lacking CHD4, a proportion of NPCs precociously exit the cell cycle, fail to differentiate, and die.

**Figure 3:**
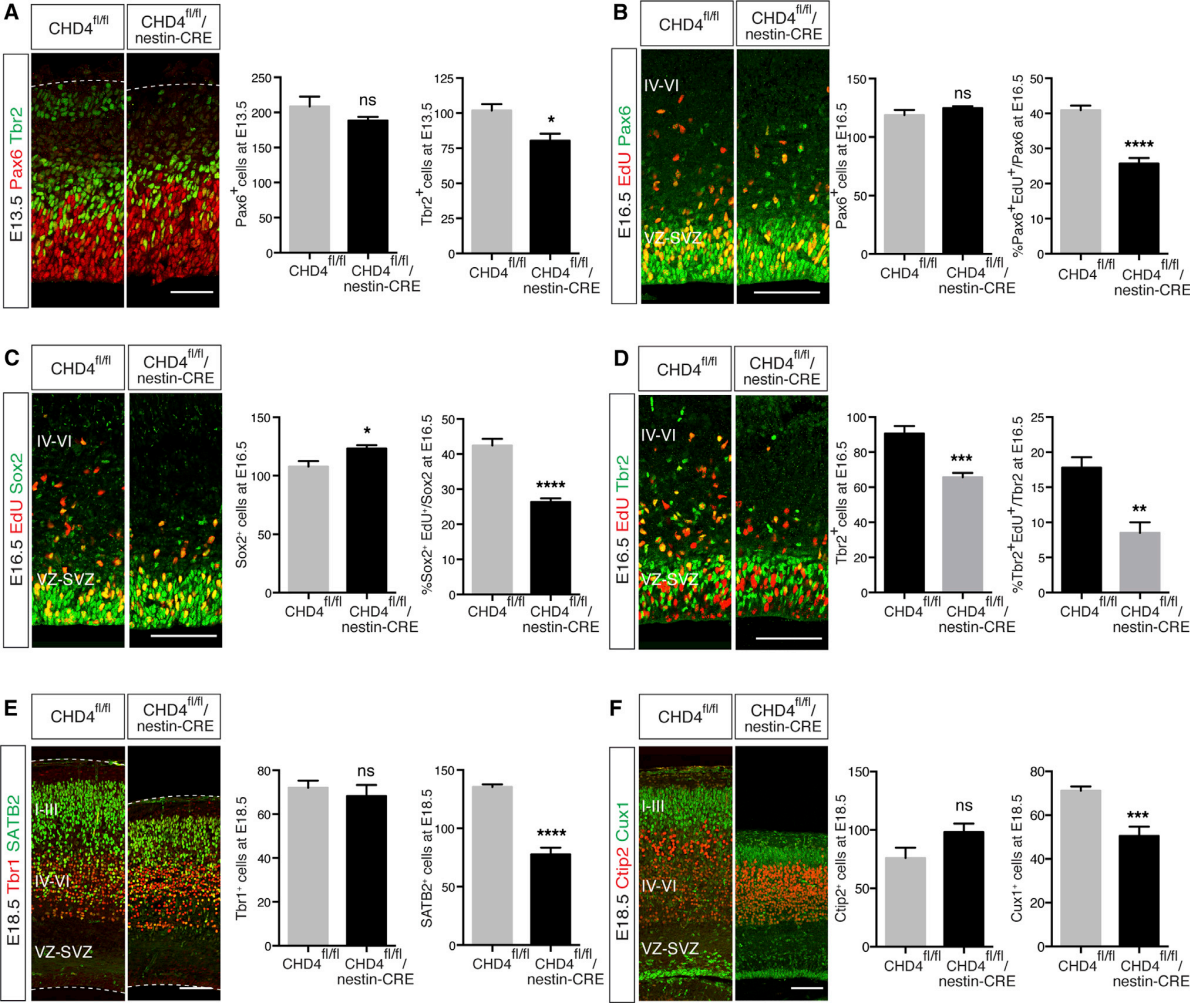
Deletion of CHD4 Causes IPCs Depletion and Defects of Cortical Lamination (A) Left: coronal sections of E13.5 CHD4^fl/fl^/nestin-CRE and CHD4^fl/fl^ control brains immunostained for Pax6 (red) and Tbr2 (green). Scale bar, 50 μm. Right: quantification of Pax6- and Tbr2-expressing cells per section. 5–12 embryos obtained from five litters were analyzed per genotype. (B–D) Left: coronal sections of E16.5 CHD4^fl/fl^/nestin-CRE and control brains harvested 2 hr after EdU injection and immunolabeled with EdU (red) and Pax6 (B), Sox2 (C), or Tbr2 (D) (green). Scale bar, 100 μm. Right: number of cells expressing Pax6 (B), Sox2 (C), and Tbr2 (D) per section and percentage of cells co-labeled with EdU. Five to six embryos obtained from three litters were analyzed per genotype. (E and F) Left: coronal sections of E18.5 CHD4^fl/fl^/nestin-CRE and CHD4^fl/fl^ control brains immunolabeled with Tbr1 (red) and SATB2 (green) (E) or Ctip2 (red) and Cux1 (green) (F). Scale bar, 100 μm. Right: quantification of cells expressing Tbr1 and SATB2 (E) or Ctip2 and Cux1 (F) in each section. Littermates were obtained from five litters, and five to ten embryos were analyzed per genotype. All data are presented as mean ± SEM. ^∗^p < 0.05, ^∗∗^p < 0.01, ^∗∗∗^p < 0.001, and ^∗∗∗∗^p < 0.0001 (ns, not significant) by unpaired t test. See also Figure S5.

Progenitor cells, such as radial glia (RG) and apical progenitors (APs), are located in the VZ and express the transcription factors Pax6 and Sox2 (Götz et al., 1998, Graham et al., 2003). To study whether deletion of CHD4 affected NPC number, cortices of CHD4^fl/fl^/nestin-CRE mice and control littermates were immunostained for Pax6 and Sox2. At E13.5 and E16.5, the number of cells expressing Pax6 in the VZ was unchanged (Figures 3A and 3B), whereas at E16.5, the number of Sox2-expressing cells was slightly increased (Figure 3C). However, when Pax6 and Sox2 immunostaining were combined with a 2 hr EdU pulse, it revealed a significant reduction in the number of actively proliferating apical progenitors in CHD4^fl/fl^/nestin-CRE mice (Figures 3B and 3C). RG and APs divide at the apical surface and give rise to neurons as well as another neuronal progenitor cell type called intermediate progenitor cells (IPCs, also known as basal progenitors) (Noctor et al., 2004). IPCs divide in a basal position within the VZ and subventricular zone (SVZ) and express the transcription factor Tbr2 (also known as EOMES) (Englund et al., 2005). In lissencephalic rodents, IPCs represent the principal neurogenic cells and are considered to be responsible for the amplification of neuronal cell number that drove cortical expansion during evolution (Martínez-Cerdeño et al., 2006). To investigate whether CHD4 influences IPC number, cortices of CHD4^fl/fl^/nestin-CRE mice were immunostained for Tbr2 and analyzed using confocal microscopy. In the absence of CHD4, the number of Tbr2-expressing cells was reduced at E13.5 and E16.5 (Figures 3A and 3D), and EdU pulse experiments demonstrated a striking reduction of proliferating Tbr2-positive IPCs at E16.5 (Figure 3D). Because symmetric division of IPCs is necessary for the expansion of neuronal output their depletion may account for the cortical thinness observed in CHD4^fl/fl^/nestin-CRE mice.

### Deletion of CHD4 Alters Cortical Lamination

To ask how IPC depletion affected the formation of cortical layers we analyzed a number of markers specific for either upper (II and III) or deeper (IV–VI) layer neurons. E18.5 CHD4^fl/fl^/nestin-CRE and control cortices were immunostained for upper (SATB2 and Cux1) (Alcamo et al., 2008, Britanova et al., 2008, Nieto et al., 2004) or deeper (Tbr1 and Ctip2) (Arlotta et al., 2005, Hevner et al., 2001) layer-specific markers. In the absence of CHD4, the number of neurons expressing Tbr1 and Ctip2 was unchanged (Figures 3E and 3F). In contrast, upper layer thickness and the number of neurons expressing SATB2 and Cux1 were greatly reduced (Figures 3E and 3F). Although IPCs generate neurons that populate all cortical layers (Sessa et al., 2008), they are the most abundant cell type in the SVZ, which develops between E13 and E15, at a time when deeper layer neurons are already generated. Thus, in CHD4^fl/fl^/nestin-CRE mice IPC depletion in the SVZ severely and specifically affects the formation of the upper layers of the cortex.

### CHD3, CHD4, and CHD5 Regulate Distinct Aspects of Neural Radial Migration

**Figure 4:**
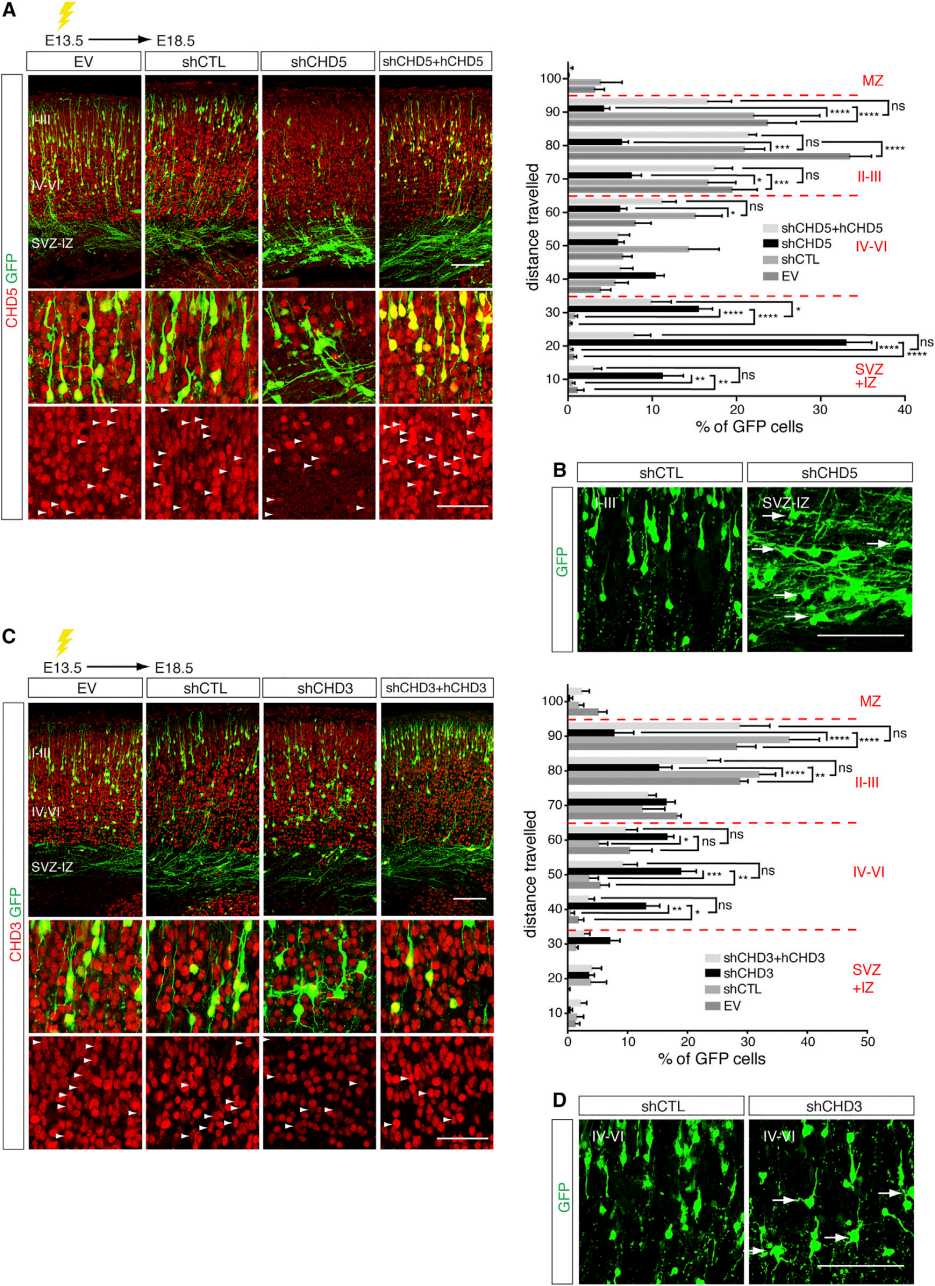
CHD5 and CHD3 Regulate Distinct Aspects of Neural Radial Migration (A) Left: E13.5 embryos were in utero electroporated with the indicated shRNA-GFP vectors, and electroporated cells (green) expressing CHD5 (red) were analyzed at E18.5. Scale bar, 100 μm. Bottom: higher-magnification images of the sections. Arrowheads indicate electroporated cells. Scale bar, 50 μm. Right: quantification of electroporated cells traveling the distance between ventricular surface (0) and pial surface (100). 5–13 embryos were analyzed per condition; n = 3. (B) Representative images of neurons within the layers II–III or SVZ-IZ electroporated with shRNA-GFP vectors. Arrows indicate multipolar neurons. Scale bar, 100 μm. (C) Left: E13.5 embryos were in utero electroporated with shRNA-GFP vectors and CHD3 expression (red) in electroporated cells was analyzed at E18.5. Scale bar, 100 μm. Bottom: higher-magnification images of the sections. Arrowheads indicate electroporated cells. Scale bar, 50 μm. Right: distribution of electroporated cells quantified as in (A). 5–11 embryos were analyzed per condition; n = 3. (D) Representative images of layers IV–VI neurons electroporated with shRNA-GFP vectors. Arrows indicate multipolar neurons. Scale bar, 100 μm. All data are presented as mean ± SEM. ^∗^p < 0.05, ^∗∗^p < 0.01, ^∗∗∗^p < 0.001, and ^∗∗∗∗^p < 0.0001 (ns, not significant) by two-way ANOVA with Tukey’s multiple comparisons test.

Contrary to CHD4, CHD3, and CHD5 expression is very low in neural progenitors and increases steadily during the late stages of neurogenesis, when neurons migrate radially to populate the CP (Figure S2). In the developing cortex, CHD3 and CHD5 show similar, albeit not entirely overlapping, expression profiles. CHD3 is expressed in neurons that have reached the CP, whereas CHD5 was also detected in the SVZ (Figure S2C). To ask whether CHD3 and CHD5 influence specific aspects of neuronal differentiation and radial migration, we employed the in utero electroporation technique. We tested the suitability of this approach by comparing the defects observed in the cortex of CHD4^fl/fl^/nestin-CRE mice with CHD4^fl/fl^ embryos subjected to in utero electroporation using either empty vector (EV) or CRE-containing vector. Brains were electroporated at E14.5 and Tbr2 expression was analyzed 24 hr later. Electroporation with the CRE vector resulted in a striking reduction of Tbr2-positive cells comparable to CHD4^fl/fl^/nestin-CRE mice (Figures S5A and 3D). Similarly, electroporation of E14.5 brains with a CHD4-specific small hairpin RNA (shRNA) decreased both NPC proliferation and the number of IPCs (Figures S5B–S5D) compared to control shRNA (shCTL). Next, we performed in utero electroporation at E13.5 using shRNA targeting either CHD5 or CHD3 (shCHD5 and shCHD3, respectively), shCTL, or an EV (Figure 4). Potential non-specific effects were ruled out by co-electroporation of rescue vectors encoding human CHD5 and CHD3. Five days after electroporation, cortices were dissected and neural migration was assessed by counting the number of GFP expressing cells that had reached the CP. NPCs are electroporated in the VZ, exit the cell cycle and migrate radially crossing the SVZ and IZ to reach their final position in the CP. Most neurons electroporated with shCHD5 accumulated within the IZ and failed to reach the CP, whereas these defects were absent in brains electroporated with EV or shCTL and were completely rescued by co-electroporation with hCHD5 (Figure 4A). Newly generated postmitotic neurons are initially multipolar and transiently grow a variable number of immature neurites. This phase precedes the establishment of neuronal polarity that is essential for radial migration (Barnes and Polleux, 2009). To test whether abnormal neuronal polarization was responsible for the defects of radial migration, we analyzed the morphology of postmitotic neurons within the SVZ of embryos electroporated with shCTL or shCHD5. Lack of CHD5 resulted in the accumulation of multipolar neurons within the IZ (Figure 4B), suggesting that CHD5 contributes to the establishment of neuronal polarity.

Because CHD3 is confined to postmitotic, differentiated neurons (Figures S2C and S4B), we reasoned that it may influence the late stages of cortical development. E13.5 embryos were electroporated with EV, shCTL, shCHD3, or shCHD3 + hCHD3 and after 5 days, cortices were immunostained for GFP and CHD3. Knockdown of CHD3 induced a delay of neuronal migration, with a significant number of cells retained in the deeper layers (IV–VI) and fewer neurons reaching the upper layers (II and III) of the cortex (Figure 4C). Similarly to CHD5, multipolar cells accumulated in the deeper layers of the CP (Figure 4D), suggesting that also in this case abnormalities of neuronal polarity may contribute to radial neural migration defects. When brains of CHD4^fl/fl^ mice were electroporated with EV or vector expressing CRE, radial migration and neuronal polarity were not affected (Figure S5E). These findings show that CHD3, CHD4, and CHD5 have non-overlapping roles during cortical development and further support the hypothesis that they are part of distinct NuRD complexes.

### CHD3 and CHD5 Differentially Regulate Cortical Layer Specification

**Figure 5:**
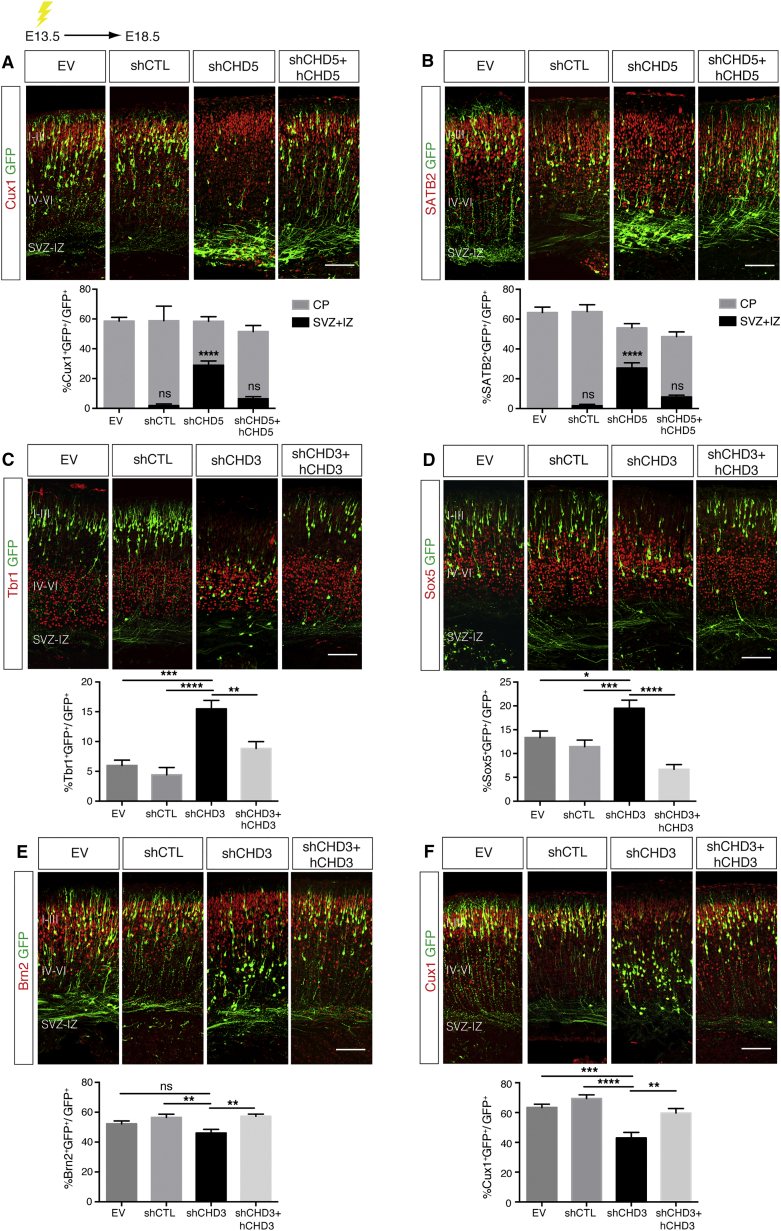
CHD3 and CHD5 Regulate Cortical Layer Specification (A and B) Cortical neurons were in utero electroporated with shRNA-GFP vectors and immunolabeled for GFP (green) and Cux1 (red) (A) or SATB2 (red) (B). Percentage of GFP-positive cells expressing Cux1 (A) or SATB2 (B) present in SVZ + IZ or CP. 6–13 embryos were analyzed per condition; n = 3. Scale bar, 100 μm. (C–F) Cortical neurons were in utero electroporated with shRNA-GFP vectors and immunolabeled for GFP (green) and Tbr1 (red) (C), Sox5 (red) (D), Brn2 (red) (E), or Cux1 (red) (F). The percentage of co-labeled GFP-positive cells was quantified. Scale bar, 100 μm. 3–15 embryos were analyzed per condition; n = 3. All data are presented as mean ± SEM. ^∗^p < 0.05, ^∗∗^p < 0.01, ^∗∗∗^p < 0.001, and ^∗∗∗∗^p < 0.0001 (ns, not significant) by two way (A) and (B) or one-way ANOVA (C–F) with Tukey’s multiple comparisons test. See also Figure S5.

Neuronal differentiation, radial migration and laminar identity are known to be co-regulated processes (Kwan et al., 2012). It is therefore possible that CHD5 and CHD3 influence the transcription of genes that control both neural migration and layer positioning. We first investigated whether the early defects of neural radial migration in neurons lacking CHD5 depended on abnormal differentiation of NPCs. Brains electroporated with EV, shCTL, shCHD5, or shCHD5 + hCHD5 were immunostained for the neural progenitor markers Pax6 and Ki67. In contrast to published data (Egan et al., 2013), we did not find that knockdown of CHD5 caused persistent expression of progenitor markers (Figures S5F and S5G). Instead, in brains electroporated with shCHD5, a remarkable number of neurons that did not migrate toward the CP expressed the upper layer markers Cux1 and SATB2 (Figures 5A and 5B). Because these neurons never reach layers II to III, they give rise to an ectopic layer located beneath the CP.

We next asked whether the defects of late neural radial migration caused by inhibition of CHD3 are coupled with abnormalities of laminar identity. E13.5 embryos were electroporated with shCHD3 or control vectors and after 5 days, brains were immunostained for the layer-specific markers Tbr1, Sox5, Brn2, and Cux1. Neurons lacking CHD3 were more likely to express Tbr1 and Sox5 (Figures 5C and 5D), which are transcription factors that regulate laminar positioning and differentiation of deeper cortical layers (Hevner et al., 2001, Lai et al., 2008), whereas a lower number of neurons expressed the upper layer markers Brn2 and Cux1 (Dominguez et al., 2012, Nieto et al., 2004) (Figures 5E and 5F). Thus, despite being largely co-expressed, deletion of CHD5 or CHD3 results in strikingly different cortical defects. CHD5 is necessary during early radial migration and does not affect the expression of laminar-specific markers. In contrast, CHD3 promotes late neural radial migration and layer specification, implicating that it may influence the expression of genes that couple radial migration with laminar identity.

### Non-Redundant Functions of CHD3, CHD4, and CHD5 during Cortical Development

**Figure 6:**
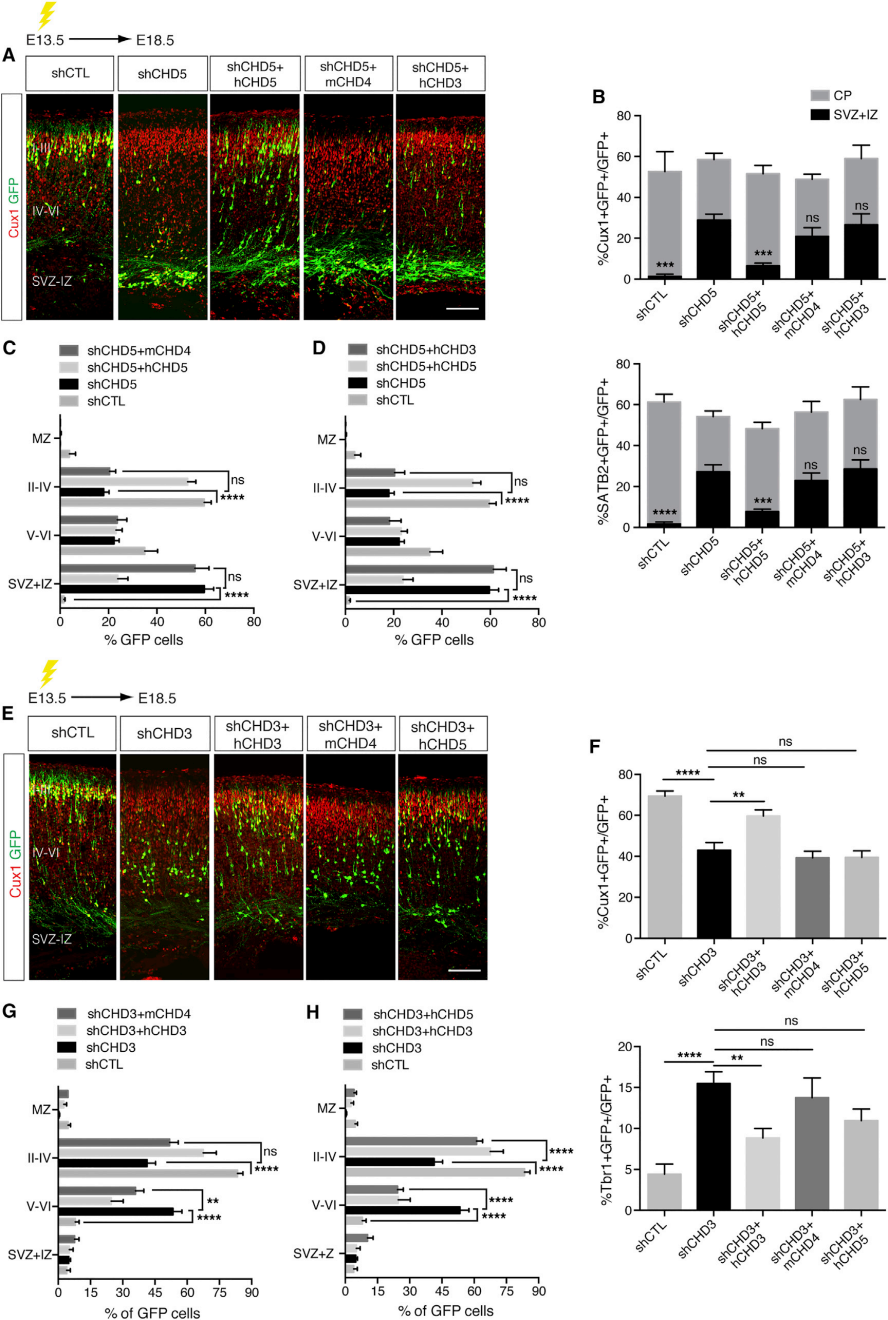
Non-redundant Functions of CHDs during Cortical Development (A) E13.5 embryos were electroporated with the indicated vectors and immunolabeled with GFP (green) and Cux1 (red) antibodies at E18.5. Scale bar, 100 μm. (B) Quantification of neurons expressing Cux1 and SATB2 in cortices electroporated as in (A). 5–13 embryos were analyzed per condition; n = 3. shCTL, shCHD5, and shCHD5 + hCHD5 conditions are identical to those shown in Figures 5A and 5B, because the experiments were performed at the same time, and data were split in two figures for clarity. (C and D) Distribution of cells electroporated with the indicated vectors at E13.5 and analyzed at E18.5. 5–13 embryos were analyzed per condition; n = 3. shCTL, shCHD5, and shCHD5 + hCHD5 conditions are identical in (C) and (D), because the experiments were performed at the same time, and data were split in two graphs for clarity. (E) E13.5 embryos were in utero electroporated with the indicated vectors and immunolabeled with GFP (green) and Cux1 (red) antibodies 5 days later. Scale bar, 100 μm. (F) Quantification of neurons expressing Cux1 and Tbr1 in cortices in utero electroporated as in (E). 8–13 embryos were analyzed per condition; n = 3. shCTL, shCHD3, and shCHD3 + hCHD3 conditions are identical to those shown in Figures 5C and 5D, because the experiments were performed at the same time, and data were split in two figures for clarity. (G and H) Distribution of cells electroporated with the indicated vectors at E13.5 and analyzed at E18.5. 8–13 embryos were analyzed per condition; n = 3. shCTL, shCHD3, and shCHD3 + hCHD3 conditions in (G) and (H) are identical, because the experiments were performed at the same time, and data were split in two graphs for clarity. All data are presented as mean ± SEM. ^∗∗^p < 0.01, ^∗∗∗^p < 0.001, and ^∗∗∗∗^p < 0.0001 (ns, not significant) by one-way (F) or two-way (B–D, G, and H) ANOVA with Tukey’s multiple comparisons test. See also Figure S6.

We reasoned that if CHD3, CHD4, and CHD5 regulate non-overlapping aspects of cortical development, the abnormalities observed in brains lacking one of them should not be reverted by co-expression of a rescue vector encoding another. First, we confirmed that mCHD4, hCHD3, and hCHD5 rescue constructs were expressed in cells transfected with shCHD3 or shCHD5. Overexpressed CHDs were easily detected in postmitotic neurons depleted of either CHD5 or CHD3 (Figures 4A, 4C, and S6A–S6C) and were incorporated within NuRD complexes under these conditions (Figure S6D). We used cortices depleted of CHD3 or CHD5, because these proteins are more likely to be functionally redundant due to a similar pattern of expression (Figure S2C). In brains co-electroporated with shCHD5 and either hCHD3 or mCHD4, the number of neurons that reached the CP (Figures 6A, 6C, and 6D) and ectopic expression of the upper layer markers SATB2 and Cux1 (Figures 6A and 6B) were comparable to neurons electroporated with shCHD5 alone. When similar experiments were performed in brains electroporated with shCHD3, expression of mCHD4 did not rescue the neural radial migration defects (Figures 6E and 6G) or change the number of cells expressing Cux1 and Tbr1 (Figures 6E and 6F). Moreover, co-electroporation of shCHD3 and hCHD5 did not influence the number of neurons expressing Cux1 or Tbr1 (Figures 6E and 6F), whereas radial migration defects were rescued under these conditions (Figures 6E and 6H), suggesting that CHD5 may provide functional compensation in regards to neuronal migration. Thus, CHD3, CHD4, and CHD5 have distinct and mostly non-redundant functions during cortical development that depend on their mutually exclusive inclusion within NuRD complexes.

### CHD Subunits Are Recruited to Distinct Gene Promoters

**Figure 7:**
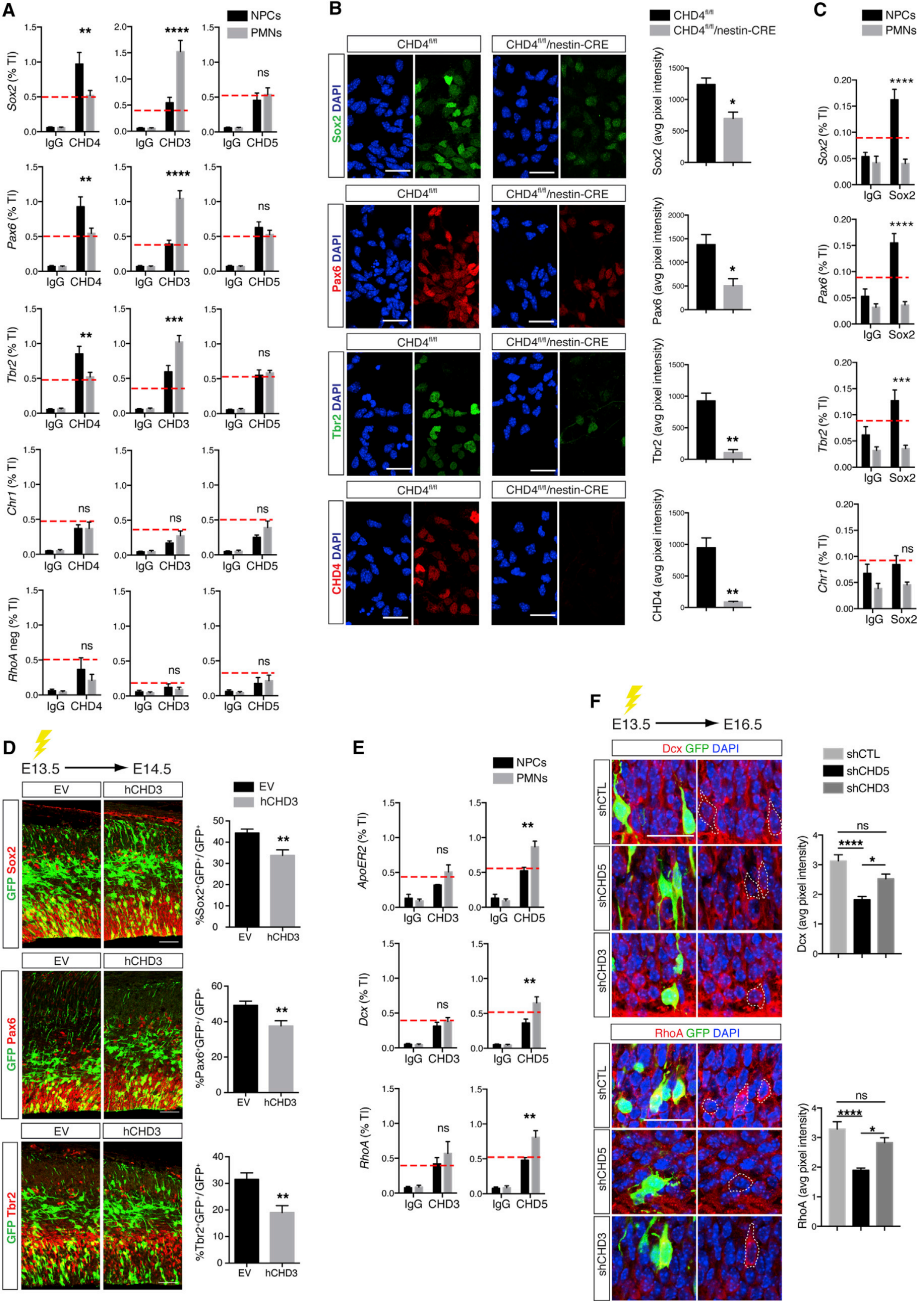
Specific CHD Subunits Regulate the Expression of Genes Necessary for Cortical Development (A) ChIP of CHDs on *Sox2*, *Pax6*, and *Tbr2* promoters in NPCs and PMNs. Chromosome 1 (*Chr1*) and *RhoA* intergenic region (*RhoA neg*) were used as negative controls. The red dotted line indicates background signal detected on *Chr1* (CHD3 ChIP n = 8, CHD4 ChIP n = 7, and CHD5 ChIP n = 7). (B) Immunofluorescence of Sox2, Pax6, and Tbr2 in NPCs derived from CHD4^fl/fl^/nestin-CRE and control E12.5 embryos; n = 3. Scale bar, 50 μm. (C) ChIP of Sox2 binding to *Pax6*, *Sox2*, and *Tbr2* promoters in NPCs and PMNs. *Chr1* was used as negative control; n = 4. (D) E13.5 embryos were in utero electroporated with either EV or hCHD3 expressing vectors and immunolabeled for GFP (green) and Sox2, Pax6, or Tbr2 (red) at E14.5. Five to nine embryos were analyzed per condition; n = 3. Scale bar, 50 μm. (E) ChIP of CHD subunits binding on *ApoER2*, *Dcx*, and *RhoA* promoters in NPCs and PMNs (CHD3 ChIP n = 6 and CHD5 ChIP n = 5). (F) Expression of Dcx and RhoA in the cortex of mice electroporated with shCTL, shCHD5, or shCHD3 at E13.5 and harvested at E16.5 25–50 cells were analyzed per embryo; n = 3. Average pixel intensity of Dcx and RhoA in GFP-expressing cells was normalized to background (ImageJ). Scale bar, 25 μm. Data are presented as mean ± SEM. ^∗^p < 0.05, ^∗∗^p < 0.01, ^∗∗∗^p < 0.001, and ^∗∗∗∗^p < 0.0001 (ns, not significant) by two-way ANOVA (A, C, and E) with Sidak’s multiple comparisons test, unpaired t test (B and D), or one-way ANOVA (F) with Tukey’s multiple comparisons test. See also Figure S7.

First, we analyzed the transcriptional profile of the developing cortex by performing microarray analysis of mRNA purified from E12.5, E15.5, or E18.5 cortices (mouse WG-6 v2.0 expression BeadChip Illumina, n = 4 for condition). 30,869 transcripts were identified, 3,627 of which (corresponding to 2,835 genes) were differentially expressed during development (fold change of ±2 coupled with a false discovery rate [FDR] < 0.01) (Figures S7A and S7B; Table S2). The transcription factors Sox2, Pax6, and Tbr2 were of particular interest, as they were developmentally regulated (Figure S7C). Sox2 and Pax6 are both expressed in NPCs; Sox2 maintains apical progenitors in a proliferative state (Graham et al., 2003), and Pax6 regulates cell-cycle length of apical progenitors and IPC specification (Englund et al., 2005). Tbr2 is necessary for IPC proliferation and neurogenesis (Arnold et al., 2008), and ablation of either Pax6 or Tbr2 results in a substantial loss of cortical progenitors (Quinn et al., 2007, Sessa et al., 2008). To avoid potential confounding issues due to the cellular heterogeneity of the cortex, experiments were performed on NPCs maintained in vitro for 2 days or cultured in differentiating conditions for 7 days (postmitotic neurons [PMNs]) (Figure S4A). Binding of CHD subunits to *Sox*2, *Pax6*, and *Tbr2* promoters in NPCs and PMNs was assessed by chromatin immunoprecipitation (ChIP) assay. CHD4 was bound to *Sox2*, *Pax6*, and *Tbr2* gene promoters at much greater levels in NPCs than in PMNs (Figure 7A). Conversely, the recruitment of CHD3 to the same regions was higher in PMNs than in NPCs, whereas CHD5 binding remained unchanged. The switch of CHD4 to CHD3 binding correlated with transcriptional inhibition, suggesting that, at least for these genes, the role of NuRD complexes on gene expression may depend on the incorporation of specific CHD subunits. As expected, levels of Sox2, Pax6, and Tbr2 were remarkably reduced in NPCs obtained from CHD4 null mice (Figure 7B). To identify putative transcription factors that may be involved in the recruitment of CHD4-containing NuRD complexes to target genes, we investigated whether Sox2 was bound to promoters occupied by CHD4. Sox2 represented an interesting candidate, as it interacts with CHD4 in neural stem cells (Engelen et al., 2011). ChIP experiments demonstrated that similar to CHD4, Sox2 was recruited to *Sox2*, *Pax6*, and *Tbr2* promoters in NPCs, and binding was significantly reduced in PMNs (Figure 7C). Strikingly, ectopic expression of hCHD3 at E13.5 had an effect similar to ablation of CHD4 and caused a reduction of Pax6, Sox2, and Tbr2 expression in NPCs (Figure 7D), indicating that the composition of NuRD complexes may represent a mechanism by which Pax6, Sox2, and Tbr2 expression is developmentally regulated.

CHD5 and CHD3 binding was tested on the promoters of doublecortin (Dcx) and apolipoprotein E receptor 2 (ApoER2), two genes that regulate neural radial migration and cortical lamination (Francis et al., 1999, Gleeson et al., 1999, Trommsdorff et al., 1999). Enrichment of CHD5 was detected on both promoters in PMNs (Figure 7E), whereas CHD3 binding was unchanged in NPCs and PMNs. A similar result was observed when the promoter of *RhoA*, a gene that regulates numerous aspects of neuronal migration in the cortex (Cappello, 2013), was analyzed (Figure 7E). Moreover, electroporation of shCHD5, but not shCHD3, reduced Dcx and RhoA levels in migrating cortical neurons (Figure 7F). Thus, CHD3 and CHD5 exhibit specificity in regards to both recruitment to target genes and transcriptional regulation at these regions.

## Discussion

During brain development, chromatin remodeling is essential for the expression of genes that regulate the differentiation of pluripotent cells into mature neurons. A number of components of the BAF (Brg1- and Brm-associated factors) complex, for example, undergo subunit switch during neuronal differentiation, generating unique complexes that activate specific transcriptional programs (Yoo and Crabtree, 2009). In this study, we show that NuRD represents a major ATP-dependent chromatin remodeling complex in the developing mouse brain. NuRD has been increasingly linked with transcriptional regulation of genes necessary for cell differentiation and growth (Basta and Rauchman, 2015). In addition to transcriptional repression and silencing, NuRD has more complex effects on gene expression, including transcriptional activation, regulation of enhancers, and inactivation of activity-dependent genes (Shimbo et al., 2013, Yang et al., 2016). One important function of NuRD is to maintain progenitor cell populations and to inhibit the expression of pluripotency and Polycomb-regulated genes (Egan et al., 2013, Reynolds et al., 2012a, Reynolds et al., 2012b). At least nine CHDs have been identified so far, and several have been implicated in brain functions (Basta and Rauchman, 2015). CHD4, for example, induces presynaptic differentiation of cerebellar granule neurons and regulates the transcription of genes necessary for establishing synaptic connectivity and neurotransmission in Purkinje cells (Yamada et al., 2014). CHD4 has also been shown to be responsible for the inactivation of activity-dependent genes in the cerebellum, by promoting deposition of the histone variant H2A.z at these loci (Yang et al., 2016).

Most CHDs are expressed in neurons (Micucci et al., 2015); however, mass spectrometry analysis indicated that only CHD3, CHD4, and CHD5 are associated with NuRD in the embryonic cortex (Figures 1 and S1). Knockdown of CHD5 in the developing cortex induces severe defects of neural radial migration (Egan et al., 2013). In cortical neurons, depletion of CHD5 alters the expression of neuron-specific genes, transcription factors, and, surprisingly, the BAF45b subunit of the BAF complex (Potts et al., 2011), perhaps suggesting a functional link between two major chromatin remodeling complexes. We found that in the brain, CHD3, CHD4, and CHD5 undergo a developmentally regulated subunit exchange that results in the assembly of stage-specific NuRD complexes (Figure 1A). Importantly, deletion or inhibition of each CHD has distinct effects on cortical development. Conditional deletion of CHD4 induced premature cell-cycle exit of NPCs (Figures 2F and 2G) that led to a depletion of IPCs (Figure 3D) and may have contributed to the reduced size of the cortex observed in CHD4^fl/fl^/nestin-CRE mice (Figure 2A). Interestingly, CHD3, CHD4, and CHD5 are all detected in postmitotic neurons (Figure S3), yet they exert distinct and mostly non-overlapping functions. Lack of CHD5 affects the early stages of neural migration (Figure 4A) and induces ectopic expression of the upper layer markers Cux1 and SATB2 (Figures 5A and 5B), whereas CHD3 is necessary for the late stages of neural radial migration (Figure 4C) and laminar specification (Figures 5C–5F). Rescue experiments using human and mouse CHDs showed little functional redundancy among CHD3, CHD4, and CHD5 (Figure 6), indicating that intracellular expression of a CHD protein in and of itself is not sufficient to compensate for the specific developmental defects.

HDAC2 co-immunoprecipitated with all NuRD subunits; however, it should be noted that loss of HDAC2 is not expected to resemble the defects observed in cortices lacking CHD3, CHD4, or CHD5. HDAC2 is part of many nuclear complexes; therefore, its effect on gene expression is not limited to its inclusion within NuRD. Moreover, there is a significant functional redundancy between HDAC1 and HDAC2, perhaps even within the NuRD complex, which can recruit both deacetylases (Montgomery et al., 2009). In contrast, we found little functional compensation among CHD3, CHD4, and CHD5 (Figure 6); thus, inhibition of only one CHD is expected to induce defects that are distinct from loss of HDAC1 and/or HDAC2. Analysis of the remaining NuRD core subunits indicated that, in many instances, their inclusion within NuRD complexes is also developmentally regulated (Figure S1) and may undergo developmental switch. Of note, deletion of MBD3 results in cortical defects strikingly similar to the abnormalities observed in brains lacking CHD4 (Knock et al., 2015), suggesting that MBD3 and CHD4 may be part of NuRD complexes that regulate genes necessary for determining IPC number and upper layer specification. The majority (78%) of NuRD subunits co-sediment as a complex (Figures 1D and S1C); however, it is possible that at least some cortical defects depend on CHDs acting independently. CHD4, for example, interacts with the histone acetyl transferase p300 in thymocytes (Williams et al., 2004) and regulates the expression of the γ-globin gene in an MBD2-independent manner (Amaya et al., 2013). Further investigation will be needed to define whether there is an NuRD-independent role for CHDs during cortical development.

How do NuRD complexes containing different CHDs regulate the expression of specific genes? Chromatin remodeling factors usually lack sequence-specific DNA-binding ability; therefore, NuRD complexes may interact with transcription factors that mediate their targeting to regulatory elements, such as gene promoters and enhancers. In lymphocytes, NuRD interacts with the transcription factor Ikaros, which mediates NuRD recruitment to genes necessary for lymphoid differentiation (Zhang et al., 2011), and in neuroblastoma cell lines, NuRD complexes associate with Ctip2 (Topark-Ngarm et al., 2006). We found that in NPCs, the transcription factor Sox2 is recruited to promoters of CHD4-target genes and that this binding decreases upon differentiation to PMNs (Figure 7C). Since CHD4 and Sox2 interact in ESCs (Engelen et al., 2011), this represents a potential mechanism through which CHD4-containing NuRD complexes may be recruited to specific genomic loci. In addition, post-translational modifications of NuRD subunits may also influence the composition of NuRD complexes and their recruitment to chromatin.

Finally, our study highlights the potential role of NuRD complexes in establishing cortical connectivity. Mutations in a number of ATP-dependent chromatin remodeling enzymes have been associated with autism spectrum disorders, intellectual disability, and epilepsy (Neale et al., 2012, O’Roak et al., 2012, Allen et al., 2013). Further studies will be necessary to determine the role of ATP-dependent chromatin remodeling enzymes during the establishment of neuronal circuitry in early brain development and how alteration of their function contributes to neurodevelopmental defects.

## Experimental Procedures

### Animals

All animal experiments were approved by the UCL Animal Welfare and Ethical Review Body and carried out in accordance to appropriate UK Home Office licenses.

WT, nestin-CRE and CHD4^fl/fl^ conditional knockout mice were of C57BL/6J background. Nestin-CRE heterozygous mice were purchased from Jackson Laboratory (strain B6.Cg(SJL)-TgN(NesCre)1Kln). CHD4 homozygous mice were crossed with nestin-CRE heterozygous mice to obtain CHD4^fl/+^/nestin-CRE offspring. CHD4^fl/+^/nestin-CRE heterozygous mice were intercrossed with either CHD4^fl/+^/nestin-CRE heterozygous or CHD4^fl/fl^ homozygous mice to generate CHD4 null and control littermates.

### Whole-Sample Mass Spectrometry

HDAC2-containing complexes were immunoprecipitated with HDAC2 antibody, and immune complexes were eluted from the beads in elution buffer containing 0.5% Progenta anionic acid labile surfactant I (AALS I) (Protea). Samples were digested by the sequential addition of lys-C and trypsin proteases, desalted using C-18 StageTips, and fractionated online using microscale C18 reverse-phase chromatography as previously described (Wohlschlegel, 2009). Tandem mass spectrometry (MS/MS) spectra were acquired in a data-dependent manner on a Q-Exactive mass spectrometer (Thermo Fisher Scientific). Peptide identifications were generated from MS/MS spectra by searching the UniProt SwissProt (May 2010 release) protein database using the ProLuCID algorithm. Peptide identifications were filtered using DTASelect and required at least two peptides per protein and a decoy-database estimated false-positive rate of less than 5% (Elias and Gygi, 2007). NSAF values were calculated for each protein in order to determine the approximate degree of enrichment of putative HDAC2-interacting proteins (Florens et al., 2006).

### Nuclear Protein Extraction and Glycerol Gradient Co-sedimentation

Nuclear protein extraction from E15.5 cortices was carried out according to a protocol obtained from the Crabtree lab (http://crablab.stanford.edu/Protocolsneuronextra.htm). Extracts were run on 10%–40% glycerol gradients as previously described (Staahl et al., 2013) and analyzed by SDS-PAGE.

### In Utero Electroporation

Timed-pregnant mice (E13.5 and E14.5) were anesthetized with isoflurane in oxygen carrier (Abbot Laboratories), and uterine horns were exposed through a small incision in the ventral peritoneum. Plasmid DNA solution (2–5 μg/μL), prepared using the EndoFree plasmid purification kit (QIAGEN), was mixed with 0.05% Fast Green (Sigma) and injected through the uterine wall into the lateral ventricles of the embryos using pulled borosilicate needles and a Femtojet microinjector (Eppendorf). Five electrical pulses were applied at 35 V (50 ms duration) across the uterine wall at 950-ms intervals using 5-mm platinum tweezertrodes (Harvard Apparatus) and an ECM-830 BTX square wave electroporator (Harvard Apparatus). The uterine horns were then replaced in the abdominal cavity, and the abdomen wall and skin were sutured. Pregnant mice were sacrificed 24 hr, 3 days, or 5 days following surgery, and embryos were subjected to immunofluorescence analyses to study neural radial migration and expression of proliferation and laminar-specific markers.

### Statistical Analysis

Data are presented as mean ± SEM. Statistics for multiple comparisons was performed using either unpaired t test or one- or two-way ANOVA followed by appropriate post-test indicated in the figure legends. All analysis was performed using GraphPad Prism version 6.0 for Macintosh (GraphPad Software) (^∗^ p < 0.05, ^∗∗^ p < 0.01, ^∗∗∗^ p < 0.001, and ^∗∗∗∗^ p < 0.0001 for all statistical analysis).

## Author Contributions

J.N. performed most experiments and helped conceive the project and write the manuscript. J.G.S. performed experiments shown in Figures 1C, 1D, 7D, S1, S4, and S7 and helped write the manuscript. W.T.S. performed the experiments shown in Figure 7B. M.M.G.H. helped with in utero electroporation. A.N. performed the initial co-immunoprecipitation experiments. W.D.B., A.A.V., and J.A.W. performed the mass spectrometry, and R.M. was responsible for bioinformatics analysis. A.R. conceived the project and wrote the manuscript.

## Accession Numbers

The accession number for the microarray data reported in this study is GEO: GSE70298.

## Supplemental Information

Document S1. Supplemental Experimental Procedures and Figures S1–S7

Table S1. Proteins Interacting with HDAC2 in the Developing Embryonic Cortex, Related to Figures 1 and S1

Table S2. Differentially Expressed Genes in the Developing Mouse Embryonic Cortex, Related to Figures 7 and S6

Document S2. Article plus Supplemental Information
