# Supplementary material for: A Functional Switch of NuRD Chromatin Remodeling Complex Subunits Regulates Mouse Cortical Development
Source: Cell Rep. 2016 Nov 1;17(6):1683–98. doi: 10.1016/j.celrep.2016.10.022 (PMC5149529; doi:10.1016/j.celrep.2016.10.022)
Supplement: Document S1. Supplemental Experimental Procedures and Figures S1–S7 [file mmc1.pdf]

Cell Reports, Volume *17*

Supplemental Information

A Functional Switch of NuRD
Chromatin Remodeling Complex Subunits
Regulates Mouse Cortical Development

Justyna Nitarska, Jacob G. Smith, William T. Sherlock, Michele M.G. Hillege, Alexi Nott, William D. Barshop, Ajay A. Vashisht, James A. Wohlschlegel, Richard Mitter, and Antonella Riccio

A

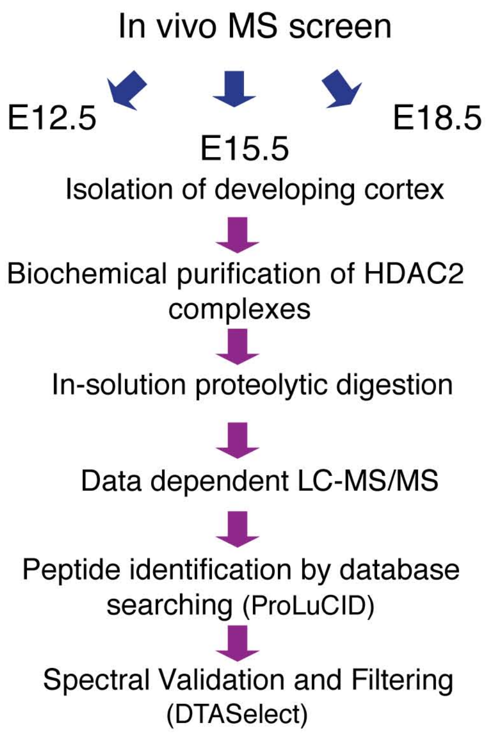

B

	Name	Locus ID	MW	E12.5	E15.5	E18.5
NuRD	HDAC2	spIP70288IHDAC2_MOUSE	55302	975.513	595.091	634.144
	HDAC1	spIO09106IHDAC1_MOUSE	55075	631.452	248.650	351.115
	CHD3	trIB1AR17IB1AR17_MOUSE	232744	337.988	439.650	437.652
	CHD4	trIE9QAS4IE9QAS4_MOUSE	216369	984.385	493.971	678.162
	CHD5	trIE9PYL1IE9PYL1_MOUSE	222683	201.085	256.550	181.902
	MTA1	trIF8WHY8IF8WHY8_MOUSE	79189	1319.318	805.688	907.494
	MTA2	spIQ9R190IMTA2_MOUSE	75030	1203.327	848.772	1056.828
	MTA3	trIE9Q794IE9Q794_MOUSE	58454	926.168	573.957	620.881
	MBD2	spIQ9Z2E1IMBD2_MOUSE	43501	131.953	111.343	81.757
	MBD3	trID3YTR5ID3YTR5_MOUSE	29070	1209.492	782.440	838.603
	GATA2a	trIE9QMN5IE9QMN5_MOUSE	67462	1078.565	446.680	591.456
	GATA2b	spIQ8VHR5IP66B_MOUSE	65411	998.504	783.786	830.312
	Rbbp4	spIQ60972IRBBP4_MOUSE	47656	3415.442	2917.601	3606.601
	Rbbp7	spIQ60973IRBBP7_MOUSE	47790	2038.247	1041.226	1228.747
CoREST	CoREST1	trIK3W4P9IK3W4P9_MOUSE	52715	471.498	230.480	332.430
	CoREST2	spIQ8C796IRCOR2_MOUSE	57908	626.716	273.226	536.234
	CoREST3	spIQ6PGA0IRCOR3_MOUSE	49779	138.432	81.767	0
mSin3	Sin3a	spIQ60520ISIN3A_MOUSE	145088	336.912	296.693	288.451
	Sin3b	spIQ62141ISIN3B_MOUSE	126405	106.613	205.710	162.940
	Sds3	spIQ8BR65ISDS3_MOUSE	38107	214.137	196.751	221.129
	Sap130	trIJ3QNK5IJ3QNK5_MOUSE	111240	66.449	82.859	82.343
	Sap30L	spIQ5SQF8ISP30L_MOUSE	20745	0	50.655	53.136

C

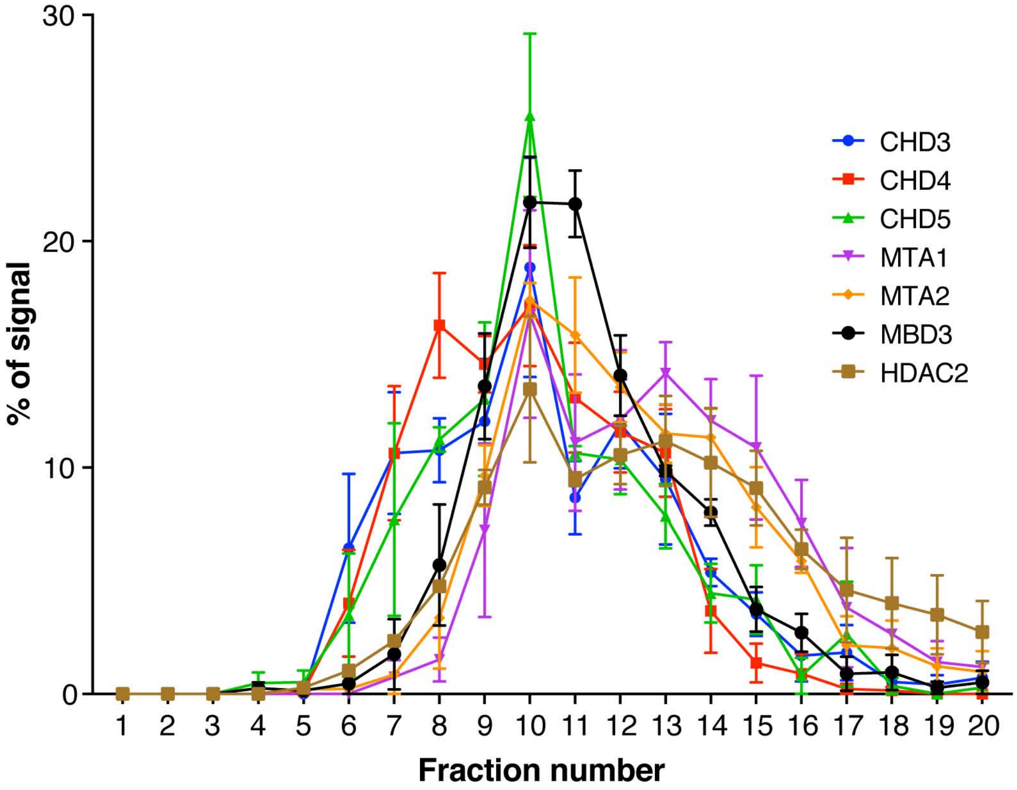

Figure S1. Related to Fig.1 NuRD complex subunits co-immunoprecipitate with HDAC2 in the developing cortex (**A**) Workflow of purification and identification of proteins that co-immunoprecipitate with HDAC2 in the embryonic cortex at the indicated stages. (**B**) List of the most abundant proteins that interact with HDAC2 identified by mass spectrometry. The numbers shown represent the normalised spectral abundance factor (NSAF) for each protein. MW, molecular weight. Complete list of HDAC2 interacting proteins can be found in **Table S1**. (**C**) Densitometry analysis of the glycerol gradient analysis represented in **Fig.1D**. For each protein, the percentage of signal detected in each fraction was assessed using Image J. n=3, except for fraction 11 (n=2).

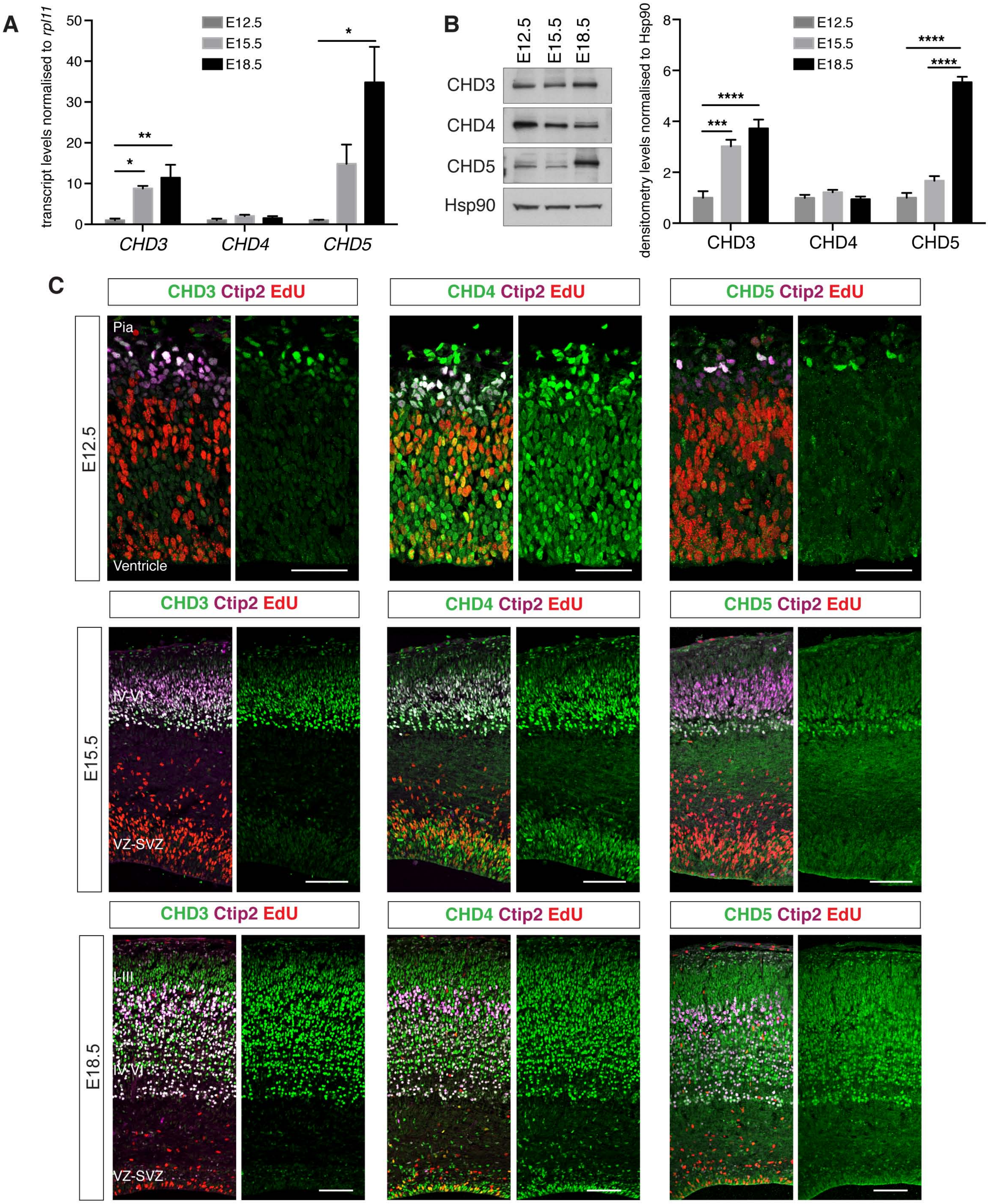

Figure S2. Related to Fig.1 CHD3, CHD4 and CHD5 proteins are differentially expressed during cortical development *in vivo*
(**A**) RT-qPCR analysis of *CHD3*, *CHD4* and *CHD5* mRNA at the indicated embryonic stages. Transcript levels were normalised to *rpl11* transcript. n=6. Data are represented as mean ± SEM. * p<0.05, ** p<0.01, ns, not significant; one way Anova with Tukey's multiple comparisons test. (**B**) Western blot and densitometry analysis of CHD3, CHD4 and CHD5 in embryonic cortex at the indicated embryonic stages. Hsp90 was used as a loading control. Data are represented as mean ± SEM of 7 independent experiments. *** p<0.001, **** p<0.0001; one way Anova with Tukey's multiple comparisons test. (**C**) Immunofluorescence analysis of CHD3, CHD4 and CHD5 (green) in cortical coronal sections dissected 2h after the administration of an EdU pulse at the indicated developmental stages. Proliferating neural progenitors are labelled with EdU (red) and deep layer neurons are labelled with Ctip2 (magenta) antibody. Bar=50 µm (E12.5) and 100 µm (E15.5 and E18.5) n=3.

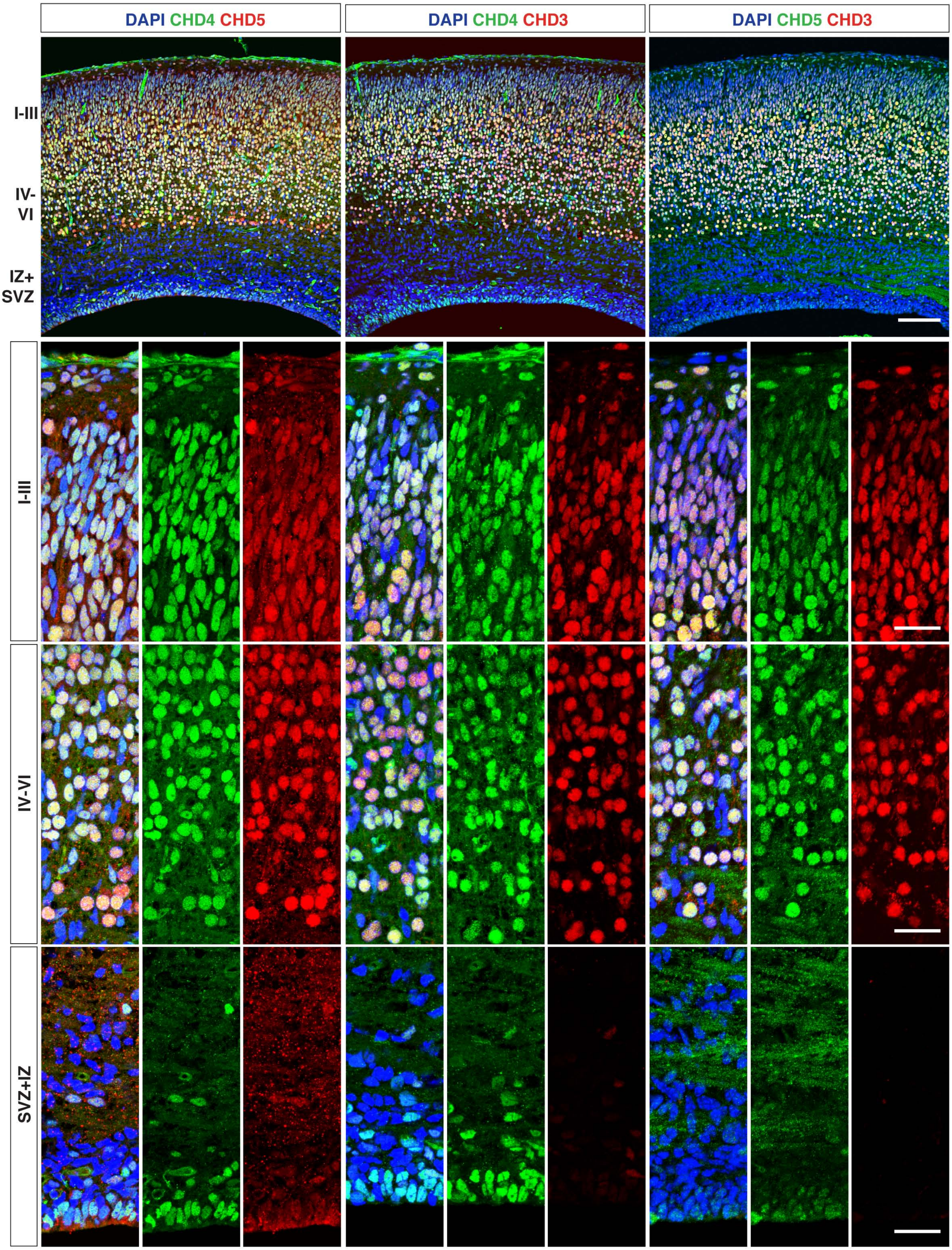

Figure S3. Related to Fig.1 CHD3, CHD4 and CHD5 proteins are co-expressed in the majority of cells within the CP. Coronal sections of E18.5 cortex immunostained with CHD3, CHD4 and CHD5. Bar=100µm. High magnification images demonstrate co-localisation of CHD4 and CHD5, CHD4 and CHD3 and CHD5 and CHD3 proteins in cells within the upper (I-III) and deeper (IV-VI) layers of the CP, as well as the SVZ. Bar= 25µm

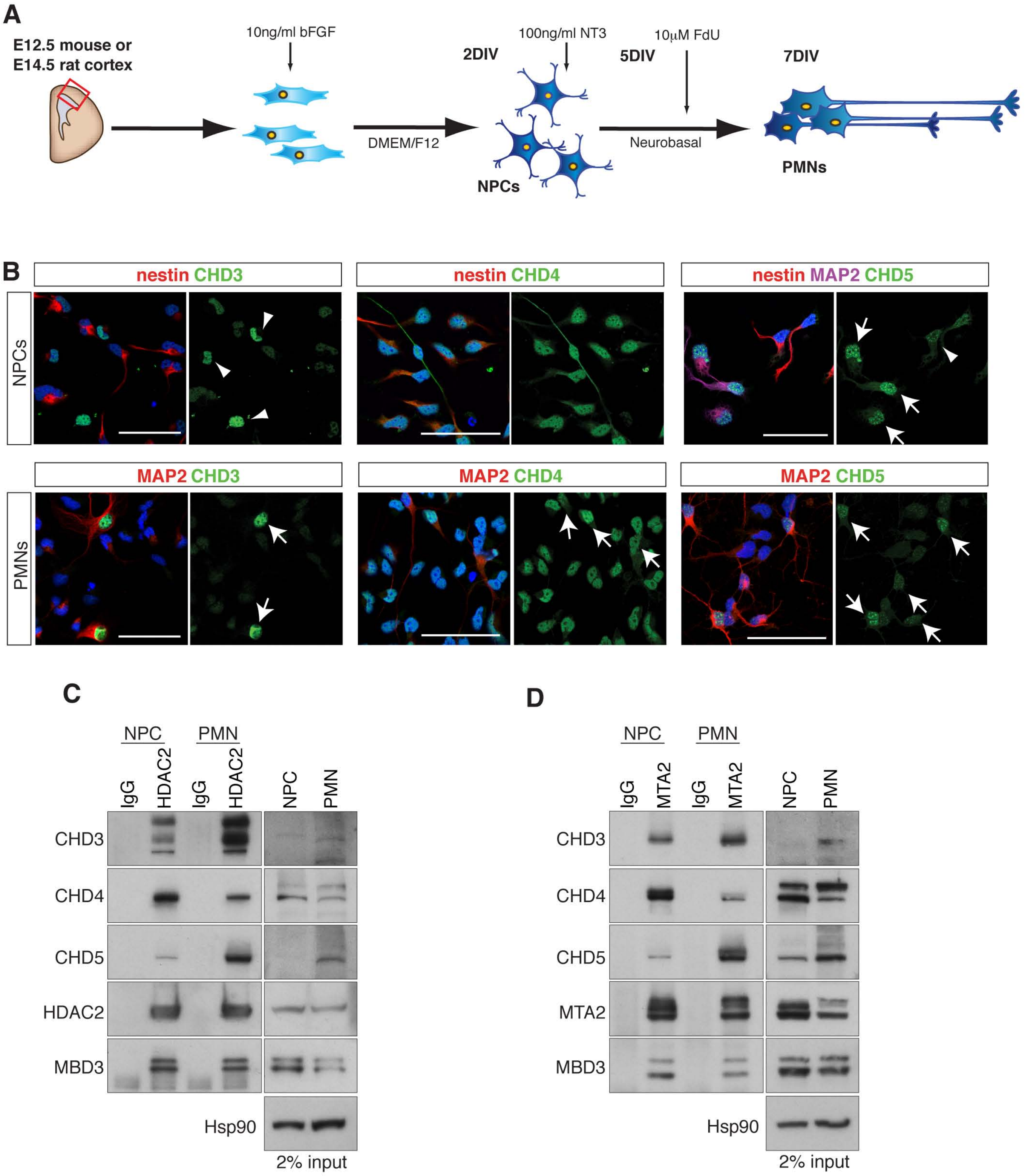

Figure S4. Related to Fig.1 CHD3, CHD4 and CHD5 proteins are differentially expressed during neuronal differentiation *in vitro*
(**A**) Diagram of cortical NPC cultures derived from E12.5 mouse or E14.5 rat embryos and differentiated into PMNs. (**B**) CHD3, CHD4 and CHD5 immunostaining (green) of rat cortical NPCs and PMNs. Neural progenitors were immunostained with nestin (arrowheads) and postmitotic neurons with MAP2 (arrows). n=3. Bar= 50µm (**C**) Western blot of proteins co-immunoprecipitating with HDAC2 in rat cortical NPCs and PMNs. Hsp90 was used as a loading control. Representative blot; n=3. (**D**) Western blot of proteins co-immunoprecipitating with MTA2 in rat cortical NPCs and PMNs. Hsp90 was used as a loading control. Representative blot; n=3

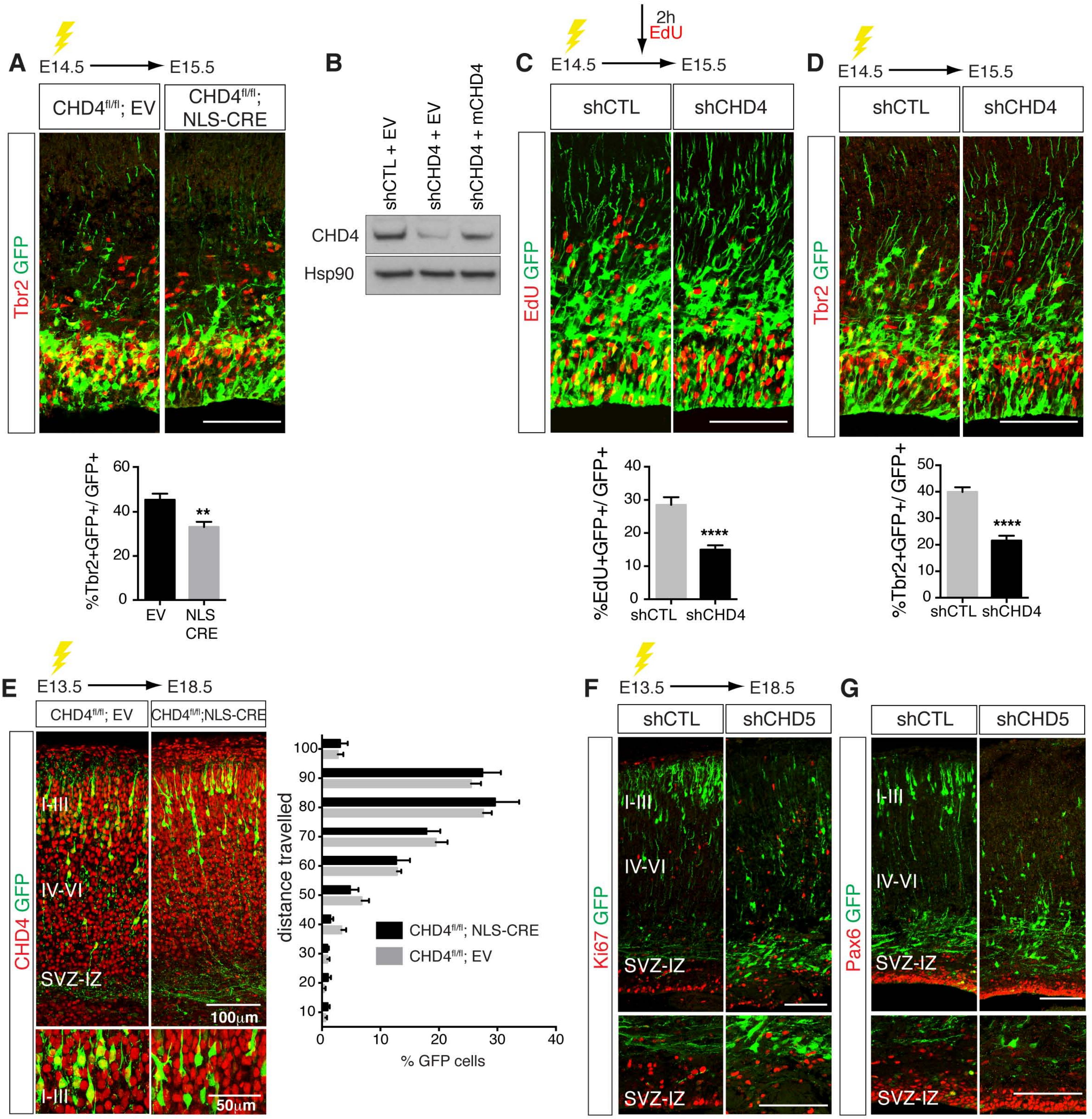

Figure S5. Related to Fig.3 and Fig.5 Proliferation analysis of cells depleted of CHD4 or CHD5 (**A**) E14.5 CHD4$^{fl/fl}$ embryos were subjected to *in utero* electroporation with GFP empty vector (EV) or NLS CRE constructs and immunolabeled with GFP (green) and Tbr2 (red) antibodies at E15.5. Shown are representative images and quantification of Tbr2 positive cells. Data were obtained from 6 to 10 embryos per condition harvested from 3 independent experiments. Bar=100μm. (**B**) shRNA designed against the 3' UTR region of CHD4 was expressed in N2A cells. Cells were cotransfected with either EV or a vector expressing a shRNA-resistant CHD4 coding sequence. CHD4 levels were assessed by western blot with Hsp90 as a loading control. Representative blot of n=3. (**C**) E14.5 embryos were *in utero* electroporated with shCTL or shCHD4 constructs. Embryos were subjected to a 2h EdU pulse and harvested at E15.5 then immunolabeled with GFP (green) antibody and EdU (red). Shown are representative images and quantification of EdU positive cells. Data were obtained from 6 to 14 embryos per condition, harvested from three independent experiments. Bar=100μm. (**D**) E14.5 embryos were *in utero* electroporated with either shCTL or shCHD4 constructs and immunolabeled with GFP (green) and Tbr2 (red) antibodies at E15.5. Shown are representative images and quantification of Tbr2 positive cells. Data were obtained from 10 to 14 embryos per condition harvested from 3 independent experiments. Bar=100μm. (**E**) E13.5 CHD4$^{fl/fl}$ embryos were *in utero* electroporated with either EV or NLS CRE constructs and analysed at E18.5. Shown are representative images of coronal sections immunostained for CHD4 (red) and GFP (green) and quantification of the migration of electroporated neurons at E18.5. Data were obtained from 8 embryos per condition harvested from 3 independent experiments. Bar=100μm (top) and 50μm (bottom). (**F and G**) E13.5 embryos were subjected to *in utero* electroporation with indicated shRNA-GFP constructs and analysed at E18.5. Shown are representative images of coronal sections stained for GFP (green) and Ki67 (red) (**F**) or Pax6 (red) (**G**). Bar= 100μm; n= 5. Data are represented as mean ± SEM. ** $p<0.01$, **** $p<0.0001$, unpaired t test.

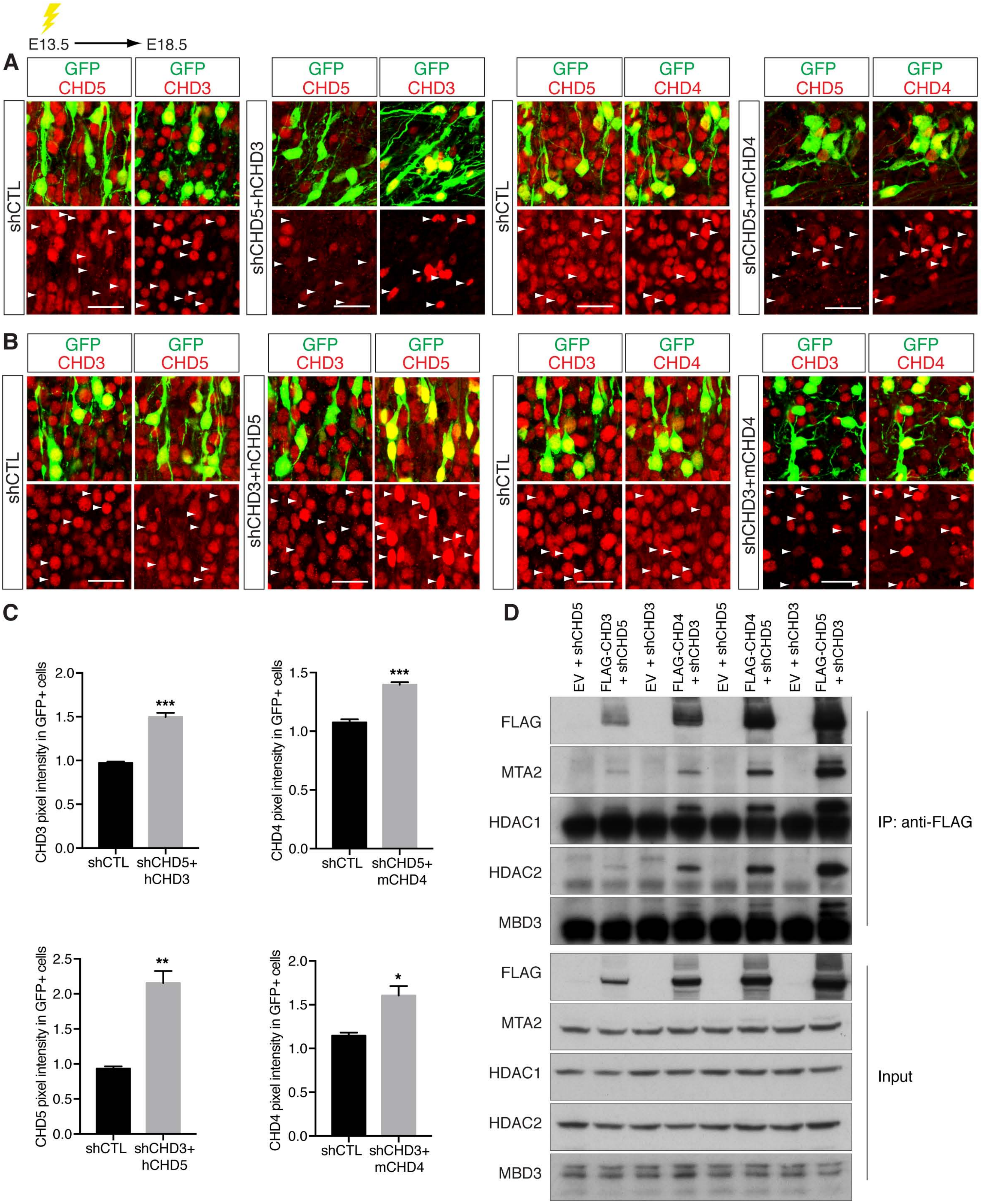

Figure S6. Related to Fig.6 Overexpression of CHD rescue constructs leads to the formation of NuRD complexes
(**A**) Immunofluorescence analysis of E13.5 embryos *in utero* electroporated with either shCTL or shCHD5 and the indicated CHD rescue constructs and analyzed at E18.5 (as in **Fig.6A**). Bar=25µm (**B**) Immunofluorescence analysis of E13.5 embryos *in utero* electroporated with either shCTL or shCHD3 with and the indicated rescue constructs and analyzed at E18.5 (as in **Fig.6E**). Bar=25µm (**C**) Quantification of pixel intensity of the experiments shown in (**A** and **B**). Average pixel intensity of overexpressed CHD proteins in GFP-expressing cells was normalised to average signal of the endogenous protein measured in neighbouring GFP-negative cells. 25-50 GFP positive and GFP negative cells analysed per embryo, n=3. Data are represented as mean ± SEM. * $p<0.05$, ** $p<0.01$, *** $p<0.001$; unpaired t-test (**D**) N2A cells were transfected with FLAG tagged forms of CHD3, CHD4 or CHD5 along with shRNAs against CHD3 and CHD5, as indicated. Co-immunoprecipitation was carried out using an anti-FLAG antibody, followed by western blotting for HDAC1, HDAC2, MTA2 and MBD3. Representative blot, n=3.

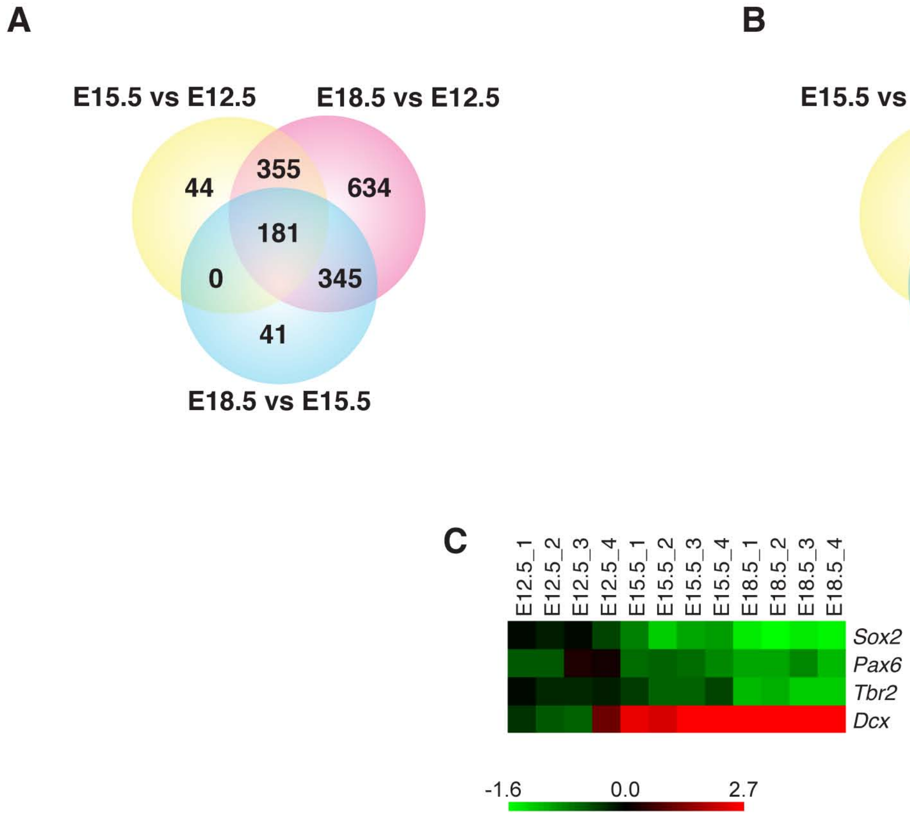

Figure S7. Related to Fig.1 and Fig.7 Microarray analysis of transcripts expressed during mouse cortical development (**A-B**) Venn diagrams of the 2035 genes upregulated (**A**) and 1613 genes downregulated (**B**) during cortical development. (**C**) Heatmap representing the log base 2 transformed expression levels of 4 putative NuRD target genes at the indicated embryonic stages. Colour scale bar represents log base 2 fold changes between each replicate and E12.5 group [2log(expression sample) – 2log(average expression E12.5 group)]; red, up-regulation; green, down-regulation; black, no change; n = 4.

SUPPLEMENTAL TABLES

Table S1. Proteins interacting with HDAC2 in the developing embryonic cortex. Related to Figure 1 and S1. The list of proteins identified as HDAC2 interactors in the E12.5, E15.5 and E18.5 embryonic mouse cortex. The list comprises NSAF values for proteins identified in the HDAC2 samples but absent from the normal IgG controls. Two biological repeats were analyzed for each time point. Components of the NuRD complex are highlighted in red.

Table S2. Differentially expressed genes in the developing mouse embryonic cortex. Related to Figure 7 and S6. The list of 2835 significantly differentially expressed genes identified by performing pairwise comparisons between each two developmental time points analyzed, (E15_Vs_E12, E18_Vs_E12 and E18_Vs_E15)**.** FDR controlled p-value threshold of <0.01 together with a +/- 2 fold change was applied to assess significance, cortices from four independent embryos per each condition were analyzed.

SUPPLEMENTAL EXPERIMENTAL PROCEDURES

Co-immunoprecipitation

Co-immunoprecipitation experiments were performed using mouse embryonic cortices or rat cortical progenitor cultures. Cells were plated on 90mm dishes and after 5 days in culture, were washed with cold PBS and harvested in cold RIPA buffer (50mM Tris pH 7.5, 150mM NaCl, 1% NP40, 0.5% Deoxycholate) containing a protease inhibitor cocktail (Sigma). Cells or homogenized cortices were lysed in cold RIPA buffer containing the protease inhibitor cocktail for 30 minutes on ice, and lysates were sheared using 23G needle and cleared by centrifugation at 1000xg for 10 minutes at 4ºC. Protein concentration was determined using BCA Protein Assay (Thermo Scientific), according to the manufacture instructions and 0.5-1mg of protein was used for each co-immunoprecipitation. Lysates were pre-cleared with protein G-Sepharose (GE Healthcare Life Science) for 1 hour at 4^0C and incubated with the following antibodies: rabbit anti-CHD3 (5μg, Bethyl A301-220A), rabbit anti-CHD4 (2μg, Abcam ab72418), rabbit anti-CHD5 (5μL, generated by the Pazin Lab), rabbit IgG (Dako X 0903), mouse anti-HDAC2 (4μg, Abcam ab12169), mouse IgG (Santa Cruz sc2025), rabbit anti-MTA2 (5μg, Abcam ab8106) and mouse anti-FLAG (2.8μg, Sigma F31650). Lysates were rotated overnight at 4ºC and immune complexes were collected with protein G–Sepharose beads (2 hours at 4ºC). Beads were washed 4 times with washing buffer (50mM Tris pH 7.5, 150mM NaCl, 0.1% TX100, 5% glycerol) and two times with PBS. Proteins were eluted by boiling the beads with 2x Laemmli buffer (2% SDS, 20% glycerol, 2% 2-Mercaptoethanol, 0.006% bromophenol blue and 0.125 M Tris HCl, pH 6.8) and samples were analyzed by SDS-polyacrylamide gel electrophoresis (SDS-PAGE).

Cortical progenitor cultures

E14.5 rat cortices or E12.5 mouse cortices were isolated in dissociation buffer (2.5mM Hepes pH 7.4, 30mM glucose, 98mM Na_2SO_4, 1mM $CaCl_2$, 1mM $MgSO_4$, 4mM $NaHCO_3$, 1xHBSS) supplemented with 4mg/ml collagenase (Worthington, NJ) and 0.6mg/ml DNase (Sigma). Dissociated cortices were digested in dissociation media (1mM Hepes pH 7.4, 20mM glucose, 98mM Na_2SO_4, 30mM K_2SO_4, 5.8mM $MgCl_2$, 0.25mM $CaCl_2$, 0.001% Phenol red, 0.126mN NaOH) supplemented with 20U/ml of papain (Wortington) for 25min at 37^0C. After digestion, cortices were washed, dissociated and plated on Nunc dishes (Thermo Scientific) or glass coverslips coated with 40μg/ml poly-D-lysine (Sigma) and 2 μg/ml Laminin (BD Bioscience) in DMEM/F12 medium supplemented with 1x B27, 1x N2, 1mM glutamine and 1mM $NaHCO_3$. Plating medium was initially supplemented with 10ng/ml of bFGF (Life technologies). Cells were plated at $1.25*10^6$ cells per 90mm dish and $2.5*10^4$ cells on glass cover slips in 4 well plates. After 2 days *in vitro*, half of the medium was changed into Neurobasal medium with 1x B27, 1mM glutamine and supplemented with 100ng/ml NT3 (Alomone labs). NT3 was supplemented every 3 days. After 5 days cells were supplemented with 10μM 5-Fluoro-2′ -deoxyuridine (FdU). Cells were maintained in 37ºC, 5% CO_2 incubators for up to 7 days.

Western blotting

Tissues were homogenized using a hand-potter homogeniser (Sigma) in cold PBS. Cells were lysed in RIPA buffer containing a protease inhibitor cocktail (Sigma) on ice and sonicated for 5sec (Branson Sonifier 450). Proteins were measured using a BCA Protein Assay (Thermo Scientific). Prior to loading on gel, samples were boiled with 5 x Laemmli sample buffer (0.3125M Tris-HCl, pH 6.8, 5% SDS, 50% Glycerol, 5% 2-Mercaptoethanol and 0.015% bromphenol blue) for 5 min Samples were separated either on 8% polyacrylamide gels (Bio-Rad) or precast NuPAGE Novex 4-12% Bis-Tris gels (Life Technologies) and transferred onto PVDF membrane (GE Healthcare Life Science) using the Mini Trans-Blot tank transfer system (Bio-Rad) for 3h at 4ºC at constant 100V. Membranes were incubated with 5% milk in TBST (50mM Tris pH 7.6, 150mM NaCl, 0.1% Tween-20) for 1h at room temperature followed by primary antibodies diluted 1:1000 in 5% milk in TBST overnight at 4ºC. The primary antibodies used were goat anti-Hsp90 (Santa Cruz sc1055), rabbit anit-CHD3 (Abcam 109195), rabbit anti-CHD4 (Active Motif 39289), rabbit anti-CHD5 (gift from M. Pazin), rabbit anti-HDAC2 (Santa Cruz sc-7899), rabbit anti-HDAC1 (Abcam ab19845-100), mouse anti-MTA1 (Abcam ab50263), rabbit anti-MTA2 (Abcam ab8106), rabbit anti-MTA3 (Bethyl A300-160A), rabbit anti-p66 (Millipore 07-365), rabbit anti-RbAp46 (Abcam ab3535), rabbit anti-MBD3 (Abcam ab157464) and mouse anti-tubulin α (Sigma T9026). After three washes with TBST membranes were incubated with the appropriate secondary antibody for 2h at room temperature. Anti-mouse (GE Healthcare Life Science), anti-rabbit (GE Healthcare Life Science), anti-goat (Sigma) and anti-rat (Dako) secondary antibodies conjugated to horseradish peroxidase (HRP) were diluted 1:10000 in 5% milk in TBST. Signal was detected using ECL or ECL Prime detecting reagents (GE Healthcare Life Science) and by exposing the immunoblot to the Amersham Hyperfilm (GE Healthcare Life Science).

Plasmids

pCIG-NLSCRE plasmid was a gift from Francois Guillemot. pClneoB-3Flag-hCHD3 plasmid containing full sequence of human CHD3 was provided by Odd Stokke Gabrielsen. pCMV-SPORT6-mCHD4 plasmid containing complete coding sequence of mouse CHD4 was purchased from Open Biosystems. pDEST26-hCHD5 plasmid encoding full sequence of human CHD5 was purchased from Source Bioscience. Complete hCHD3, mCHD4 and hCHD5 coding sequences were subcloned into pCIG-IRES-GFP vector downstream of Flag tag using Gibson Assembly (NEB). In order to generate hCHD3 construct resistant to shCHD3 seven silent mutations were introduced into pCIG-hCHD3 plasmid using QuikChange Site-Directed Mutagenesis Kit (Agilent Technologies).

shRNA plasmids

For in utero electroporation experiments the oligo hairpins were ligated into pSUPER GFP plasmid (Oligoengine) using BglII/HindIII restriction sites. The shRNA sequences targeting mouse transcripts were as follows: shCHD5 5’-GATGCAAACATGTTTGTCTTG-3’, shCHD3 5’-GCCAGGCCAACAAAGTGATG-3’ and shCHD4 5’-AGTGAAAGACCCAGAGTGAT-3’. shCTL shRNA sequence 5'-GCGTACGGGGAAACTTCGA-3' was described previously (Egan, et al., 2014).

Tissue preparation

Embryonic brains were fixed using 4% paraformaldehyde (PFA) in PBS overnight at 4ºC. Fixed samples were cryoprotected using 30% sucrose overnight at 4ºC. Brains were frozen in Optimal Cutting Temperature (O.C.T, Sakura) and 10μm coronal sections were cut using a Leica cryostat.

Immunostaining

Cortical progenitor cultures were fixed with 4% PFA for 10min at RT. Fixed cells or tissue sections were permeabilised using 0.3% Triton X100 and 10% normal goat or donkey serum in PBS at room temperature for 1h and incubated with primary antibodies overnight at 4ºC. The following primary antibodies were used: rabbit anti-Ki67 (Abcam ab16667), chicken anti-GFP (Abcam ab13970), rat anti-Ctip2 (Abcam, ab18465), chicken anti-Tbr2 (Millipore AB15894), mouse anti-SATB2 (Abcam ab51502), rabbit anti-Cux1 (Santa Cruz sc13024), mouse anti-NeuN (Millipore MAB377), mouse anti-nestin (Santa Cruz sc33677), rabbit anti-MAP2 (Santa Cruz sc20172), rabbit anti-Pax6 (Covance PRB-278P), rabbit anti-Sox2 (Cell Signalling 27485), goat anti Brn2 (Santa Cruz sc-6029), rabbit anti Sox5 (Abcam ab94396), guinea pig anti Dcx (Millipore AB2253), mouse anto RhoA (Abcam ab54835), rabbit anit-CHD3 (Epitomics 2969-1), rabbit anti-CHD4 (Active Motif 39289), rabbit anti-CHD5 (gift from M. Pazin) and rat anti-CHD5 (gift from T. Tachibana). After three sequential washes with PBS, sections were incubated with Alexa Fluor conjugated secondary antibodies and 4',6-diamidino-2-phenylindole (Dapi) for 90 min at RT. Sections were washed with PBS and mounted using ProLong Gold (Life Technologies). Images were acquired using SP5 confocal microscope (Leica) with LAS AF software and processed using ImageJ software.

Cell cycle index analysis

Cell cycle exit was determined using EdU/Ki67 immunolabelling. For EdU incorporation timed pregnant females received an intraperitoneal injection of EdU (Invitrogen) at embryonic day 12 or 15. EdU was given at a dose of 20 mg/kg body weight in a solution of 10 mg/ml PBS. Embryonic brains were collected 24h after EdU injection and processed for immunostaining. Coronal sections were stained for EdU incorporation using the Click-iT EdU cocktail with AlexaFlour 555 (Invitrogen), immunolabelled with Ki67 antibody and counterstained with DAPI.

All imaging and image analysis of CHD4 null and control brains were performed blind. Images were acquired using SP5 confocal microscope with a 40X or 63X objective at 1024x1024 pixel resolution. Quantification of labeled cells within the cortical wall was performed by dividing the images into bins 200μm wide (E13.5) or 100μm wide (E16.5). The bins spanned the entire coronal section from the ventricular surface to the pia. $EdU^+/Ki67^-$ cells and total number of EdU^+ cells were counted per each bin. Cell cycle exit index was calculated as the percentage of $EdU^+/Ki67^-$ cells over the total number of EdU^+ cells.

TUNEL analysis

Fragmented DNA of apoptotic cells was labeled using the ApopTag Fluorescein Direct In Situ Apoptosis Detection assay (Millipore) following manufacturers protocol. All imaging and image analysis were performed blind. Images were acquired on SP5 confocal microscope with a 40X objective at 1024x1024 pixel resolution. A rectangular marquee with fixed width and spanning the entire cortex was used. The total number of apoptotic cells in the cortex was counted and divided by the area of the marquee to account for differences in the thickness of the cortex. Values were represented as the number of TUNEL+ cells per 1 μm^2 tissue area.

Radial neural migration analysis

Radial migration analysis of embryos electroporated *in utero* with the indicated GFP vectors was performed as described previously (Hand et al., 2005) using ImageJ and excel macro. Images were acquired on SP5 confocal microscope with a 20X objective at 1024x1024 pixel resolution. Confocal images were run through a Bandpass Filter to segment and isolate cell-sized shapes, thresholded and segmented into 10 radial regions between the ventricle and the pial surface. Individual cell position along the radial axis was recorded and imported into Excel along with the coordinates of top (pial) and bottom (ventricle) boundaries obtained using ImageJ's Path Writer plugin. Distance and percentage of migrating cells was calculated using an Excel macro.

Quantification of layer specific markers

For CHD4 null and control embryonic brains all imaging and image analysis were performed blind. Images were acquired on SP5 confocal microscope with a 40X objective at 1024x1024 pixel resolution. Confocal images of the coronal sections of the cortex were cropped into rectangle of 200µm (E13.5) or 100µm (E16.5 and E18.5) width extending from the ventricular surface to the pia. The total number of Pax6, Sox2, Tbr2, Ctip2, Tbr1, SATB2 and Cux1 positive cells within rectangle was counted for each embryo.

Measurements of pixel intensity

For CHD4 null and control cortical progenitors in culture all imaging and image analysis were performed blind. Cultures were obtained from 6 CHD4 null and 6 control E12.5 embryos from three independent litters. Images were acquired on a SP5 confocal microscope with a 63X objective at 1024x1024 pixel resolution. Three random fields were acquired per each cover slip. Approximately 300 cells from each embryo were quantified. Nuclei were highlighted using DAPI staining, than mean pixel intensity for each nuclear staining was measured in ImageJ.

For embryos *in utero* electroporated with shCTL, shCHD3 and shCHD5 all imagining and image analysis were performed blind. 25-53 cells from three independent embryos were quantified per each condition. Images were acquired on a SP5 confocal microscope with a 63X objective at 1024x1024 pixel resolution. Three coronal sections were analyzed per each embryo and four fields were acquired per each section. Electroporated cells were highlighted using GFP signal than mean pixel intensity was measured using ImageJ. Each measurement was normalized to the background signal in the nucleus of a neighboring GFP negative cell.

RNA extraction and qRT-PCR

Cortices were isolated and RNA extracted using RNeasy Mini Kit according to manufacturers protocol (Qiagen). Total RNA was eluted in 40µl DEPC water, and immediately DNase treated using TURBO DNase kit, according to manufacturers instructions (Ambion). Total RNA was reverse transcribed in a 20µl reaction volume containing random hexamer mix and Superscript III reverse transcriptase (50U, Invitrogen) at 50ºC for 1h. As a control, reverse transcription was performed without the reverse transcriptase to confirm lack of genomic DNA contamination in the samples. Resulting cDNA was used for qPCR with the DyNAmo™ Flash qPCR Kit (Thermo Scientific). All reactions were performed in triplicate with a Mastercycler® ep realplex (Eppendorf) and each experiment included a standard curve. ΔΔCt method was used for relative quantification. Results were normalized to *rpl11* transcript. Primers used for qRT-PCR: *rpl11*, Fwd GCATCCGGAGAAATGAGAAA, Rev GAAATCCAGGCCATAGATGC; *CHD3*, Fwd CCACCTTCTCAACTTCCTCACC, Rev ACATCCGCCTTGAGTCTCCGAA; *CHD4*, Fwd GGACGACGATTTAGATGTAGAG Rew CCTGGTGGTCTGTCTCATAACC; *CHD5*, Fwd TGCAACCATCCGTACCTCTTCC, Rew TCAGCACTCTGTGCCCTTCATC.

Microarray analysis

RNA from E12.5, E15.5 and E18.5 cortices were isolated using RNeasy Mini Kit (Qiagen) according to manufacturers protocol. RNA quantity and quality was analyzed using Agilent 2100 Bioanalyser. RNA samples were amplified using the TotalPrep 96-RNA amplification kit from Ambion (Applied Biosystems). Whole-genome expression profiling of the samples was performed using mouseWG-6 v2.0 Expression BeadChip (Illumina) by Cambridge Genomic Services. Raw data were processed using the Bioconductor package lumi. Briefly, data were background corrected using the "forcePositive" method, log2 transformed and then quantile normalized. Fold changes in relative expression were calculated from the 2logfc (2^(2logfc)). Three pairwise comparisons E15_Vs_E12, E18_Vs_E12 and E18_Vs_E15 were performed. A 2 fold cut-off and FDR corrected p-value ≤ 0.01 was used to identify putative transcripts that were decreased (< 1/2) or increased (> 2) between comparisons.

To identify NuRD target genes, differentially regulated transcripts were compared with previously published CHD4 ChIP-seq data (Reynolds et al., 2012b, deposited in the ArrayExpress database accession number E-MTAB-888 and Hung et al., 2012 deposited in GEO database under accession number GSE30890). The comparisons were conducted using Galaxy tool (http:/fjfj /galaxyproject.org/). 12 candidate target genes were selected and validated by ChIP performed on lysates obtained from NPCs and PMNs. Heatmaps were generated with Matrix2Png (Pavlidis and Noble, 2003). Due to the poor annotation of the rat genome and the availability of mouse ChIP-seq data, mouse genome (mm9 and mm10 assembly) was used for reference when selecting genomic regions for ChIP experiments. Selected regions were classified as promoters based on 1) their distance from the TSS and 2) RNA polymerase II binding profile provided by ENCODE ChIP-seq datasets (Robertson et al., 2007). In addition Sox2, Pax6 and Tbr2 genomic regions were selected for targeted ChIP experiments based on published CHD4 ChIP-seq data (Reynolds et al., 2012b). An intergenic region 24kb downstream of *RhoA* TSS (*RhoA* neg) and a gene desert region of rat chromosome 1 (*Chr1*) were used as negative controls.

Chromatin immunoprecipitation

ChIP was performed as described previously (Hong et al., 2008) with minor modifications. Cells were crosslinked with 1% formaldehyde for 10 min at RT. Crosslinking was terminated by adding glycine to 125mM final concentration and incubation at RT for 5 min. Samples were washed once with PBS and homogenized in hand-potter homogeniser (Sigma) in PBS. Cells were collected by centrifugation and pellets were resuspended in buffer 1 (50mM Hepes-KOH, pH 7.5, 140 mM NaCl, 1 mM EDTA, pH 8.0, 10 % Glycerol, 0.5 % NP-40, 0.25 % Triton X-100) and lysed for 10 min at 4ºC. Nuclei were pelleted and washed with buffer 2 (200 mM NaCl, 1 mM EDTA, pH 8.0, 0.5 mM EGTA, pH 8.0, 10 mM Tris-HCl, pH 8.0) and resuspended in buffer 3 (1 mM EDTA, pH 8.0, 0.5 mM EGTA, pH 8.0, 10 mM Tris-HCl, pH 8.0). All buffers contained protease inhibitor cocktail (Sigma P8340), phosphatase inhibitor cocktail (Sigma P2850 and P5726) and 1mM PMSF (Sigma). Samples were sonicated using a Bioruptor UCD-200 sonicator (Diagenode), to obtain fragments of 200bp-2kb. Cell debris was removed by centrifugation, and salt and detergent were added to the lysates to adjust buffer 3 composition to 1% Triton X-100, 0.1% DOC, 10mMTris-Cl, pH8.0, 150mM NaCl, 1mM EDTA pH 8.0, and 0.5mM EGTA, pH 8.0. Supernatants were pre-cleared by incubation with 70μl of Protein A–Sepharose beads (Amersham Biosciences) for 1 h at 4ºC. 10% of the lysate was saved as total input control. 5μg of chromatin were used per each reaction and the volume of each sample was adjusted to 500μl with ChIP lysis buffer. 5–10μg of antibody was added and samples were rotated overnight at 4ºC. The following antibodies were used: rabbit IgG (Dako X0903), mouse IgG (Santa Cruz sc2025), rabbit CHD3 (Bethyl A301-220A), mouse CHD4 (Abcam ab70469), rabbit CHD5 (gift from M. Pazin), goat Sox2 (R&D AF2018). Immune complexes were collected by incubation with 80μl of Protein A–Sepharose beads for 2h at 4ºC. Beads were collected and subjected to a series of seven sequential washes, 2 x low salt buffer (0.1% SDS, 0.5% Triton X100, 2mM EDTA, 20mM Tris-HCl pH 8.1, 150mM NaCl), 2 x high salt buffer (0.1% SDS, 1% Triton X100, 2mM EDTA, 20mM Tris-HCl pH 8.1, 500mM NaCl), 2 x LiCl buffer (10 mM Tris-HCl pH 8.1, 1 mM EDTA, 0.25 M LiCl, 1% DOC, 1% NP40) and 2 x TE (10 mM Tris-HCl pH 8.1, 1 mM EDTA) for 10min each. After a final TE wash, the supernatant was entirely removed and beads were eluted in 150μl of freshly prepared elution buffer (0.1M $NaHCO_3$ pH 8.0, 1% SDS) and vortexed for 15 min at RT. Crosslinking was reversed by adding 10μl 5M NaCl to the samples and incubating them at 65^0C over night. DNA fragments from ChIP samples and total input were purified using the QIAquick PCR purification kit (Qiagen) according to the manufacturer's protocol and eluted twice in 40μl of EB buffer. Purified DNA was subjected to qPCR analysis. qPCR was performed using the DyNAmo™ Flash qPCR Kit (Thermo Scientific). All values were calculated as a percentage of total input. Primers are available upon request.

SUPPLEMENTAL REFERENCES

Hand, R., Bortone, D., Mattar, P., Nguyen, L., Heng, J.I.-T., Guerrier, S., Boutt, E., Peters, E., Barnes, A.P., Parras, C., *et al.* (2005). Phosphorylation of Neurogenin2 Specifies the Migration Properties and the Dendritic Morphology of Pyramidal Neurons in the Neocortex. Neuron *48*, 45-62.

Hong, E.J., McCord, A.E., and Greenberg, M.E. (2008). A Biological Function for the Neuronal Activity-Dependent Component of Bdnf Transcription in the Development of Cortical Inhibition. Neuron *60*, 610-624.
Pavlidis, P., and Noble, W.S. (2003). Matrix2png: a utility for visualizing matrix data. Bioinformatics *19*, 295-296.

Robertson, G., Hirst, M., Bainbridge, M., Bilenky, M., Zhao, Y., Zeng, T., Euskirchen, G., Bernier, B., Varhol, R., Delaney, A., *et al.* (2007). Genome-wide profiles of STAT1 DNA association using chromatin immunoprecipitation and massively parallel sequencing. Nat Meth *4*, 651-657.
