# Supplementary material for: A Functional Switch of NuRD Chromatin Remodeling Complex Subunits Regulates Mouse Cortical Development
Source: Cell Rep. 2016 Nov 1;17(6):1683–98. doi: 10.1016/j.celrep.2016.10.022 (PMC5149529; doi:10.1016/j.celrep.2016.10.022)
Supplement: Document S2. Article plus Supplemental Information [file mmc4.pdf]

Article

Cell Reports

A Functional Switch of NuRD Chromatin Remodeling Complex Subunits Regulates Mouse Cortical Development

Graphical Abstract

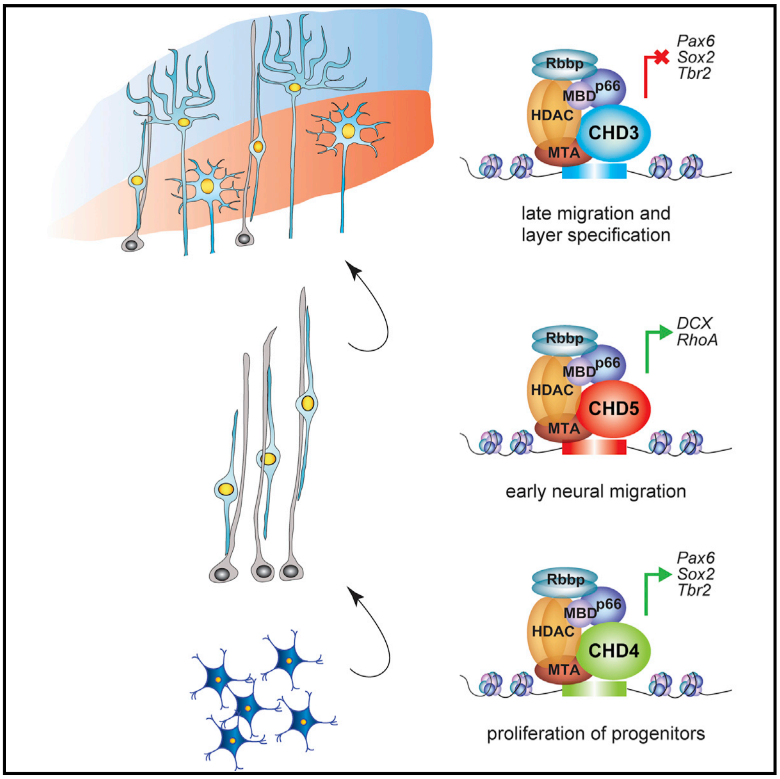

Authors

Justyna Nitarska, Jacob G. Smith, William T. Sherlock, ..., James A. Wohlschlegel, Richard Mitter, Antonella Riccio

Correspondence

a.riccio@ucl.ac.uk

In Brief

Neural development requires active chromatin remodeling. Nitarska et al. identify distinct NuRD chromatin remodeling complexes, containing CHD3, CHD4, or CHD5, during mouse embryonic cortical development. CHD4 promotes proliferation of basal progenitors, while CHD5 facilitates early radial migration and CHD3 drives late migration and laminar specification of neurons.

Highlights

- The ATPases CHD3, CHD4, and CHD5 are mutually exclusive subunits of the NuRD complex
- CHD3, CHD4, and CHD5 regulate distinct and non-redundant aspects of cortical development
- Loss of each CHD leads to specific defects of neuronal proliferation and migration
- CHD3, CHD4, and CHD5 regulate distinct set of genes essential for brain development

Accession Numbers

GSE70298

Nitarska et al., 2016, Cell Reports *17*, 1683–1698
November 1, 2016 © 2016 The Author(s).
http://dx.doi.org/10.1016/j.celrep.2016.10.022

Cell Reports
Article

OPEN ACCESS
CellPress

A Functional Switch of NuRD Chromatin Remodeling Complex Subunits Regulates Mouse Cortical Development

Justyna Nitarska,[1] Jacob G. Smith,[1] William T. Sherlock,[1] Michele M.G. Hillege,[1] Alexi Nott,[1,4] William D. Barshop,[2] Ajay A. Vashisht,[2] James A. Wohlschlegel,[2] Richard Mitter,[3] and Antonella Riccio[1,5,*]
[1]MRC Laboratory for Molecular and Cell Biology, University College London, London WC1E 6BT, UK
[2]Department of Biological Chemistry, David Geffen School of Medicine at UCLA, Los Angeles, CA 90095-1737 USA
[3]Lincoln's Inn Fields Laboratory, The Francis Crick Institute, London WC2A 3LY, UK
[4]Present address: The Picower Institute of Learning and Memory, Massachusetts Institute of Technology, Cambridge, MA 02139, USA
[5]Lead Contact
*Correspondence: a.riccio@ucl.ac.uk
http://dx.doi.org/10.1016/j.celrep.2016.10.022

SUMMARY

Histone modifications and chromatin remodeling represent universal mechanisms by which cells adapt their transcriptional response to rapidly changing environmental conditions. Extensive chromatin remodeling takes place during neuronal development, allowing the transition of pluripotent cells into differentiated neurons. Here, we report that the NuRD complex, which couples ATP-dependent chromatin remodeling with histone deacetylase activity, regulates mouse brain development. Subunit exchange of CHDs, the core ATPase subunits of the NuRD complex, is required for distinct aspects of cortical development. Whereas CHD4 promotes the early proliferation of progenitors, CHD5 facilitates neuronal migration and CHD3 ensures proper layer specification. Inhibition of each CHD leads to defects of neuronal differentiation and migration, which cannot be rescued by expressing heterologous CHDs. Finally, we demonstrate that NuRD complexes containing specific CHDs are recruited to regulatory elements and modulate the expression of genes essential for brain development.

INTRODUCTION

The ability to adjust the transcriptional output in response to ever-changing environmental conditions lies at the core of organismal development. The complex cytoarchitecture of the mammalian cortex provides a superb example of how extracellular and intracellular stimuli cooperate to transform a pool of undifferentiated neural progenitor cells (NPCs) into a highly organized layered tissue. Each cortical layer contains morphologically and functionally distinct subsets of neurons (Kwan et al., 2012) that derive from multipotent NPCs generated in the ventricular zone (VZ) of the embryonic brain. All developmental steps that lead to the formation of the mature cortex depend on the expression of specific genes that are necessary for NPC proliferation and, at later stages, neuronal migration and laminar specification.

Epigenetic modifications and changes of chromatin structure are emerging as fundamental mechanisms that regulate gene expression during brain development (Hirabayashi and Gotoh, 2010; Riccio, 2010). Chromatin is a highly dynamic structure that can be modified by a number of mechanisms, including DNA methylation, histone post-translational modifications, and ATP-dependent remodeling of chromatin (Borrelli et al., 2008). The latter mechanism uses energy released by hydrolysis of ATP to induce nucleosome sliding, facilitating the recruitment of transcriptional complexes (Narlikar et al., 2013) that activate or inhibit gene expression.

The nucleosome remodeling and deacetylase (NuRD) complex couples ATP-dependent chromatin remodeling with histone deacetylase activity (Tong et al., 1998; Xue et al., 1998; Zhang et al., 1998). NuRD is composed of six core subunits, each encoded by homologous gene families (Bowen et al., 2004). The ATPase activity of NuRD is provided by the chromodomain-helicase DNA-binding proteins (CHDs) 3, 4, or 5, and deacetylase activity by HDAC1 or HDAC2. Additional core subunits include methyl-CpG-binding domain proteins (MBDs) 1, 2, or 3; metastasis-associated proteins (MTAs) 1, 2, or 3; the histone-binding proteins Rbbp4 or Rbbp7; and the nuclear zinc-finger proteins Gata2a or Gata2b (Basta and Rauchman, 2015; Lai and Wade, 2011). NuRD has been linked to a number of basic cellular functions that take place during development, including the maintenance of genome integrity and cell-cycle progression (Lai and Wade, 2011). Recent studies have also linked NuRD to regulation of gene expression during embryonic stem cell (ESC) differentiation and lineage commitment of pluripotent cells (Kashiwagi et al., 2007; Reynolds et al., 2012a; Zhang et al., 2011). While NuRD has been implicated in the transcriptional repression of Polycomb-regulated genes in ESCs (Reynolds et al., 2012b) and in differentiated neurons (Egan et al., 2013), the role of NuRD in the developing nervous system remains largely unknown.

CrossMark

This is an open access article under the CC BY license (http://creativecommons.org/licenses/by/4.0/).

OPEN ACCESS CellPress

Here, we show that NuRD represents a major chromatin remodeling complex in the developing mouse cortex. We found that a sequential switch of CHDs within the complex results in the combinatorial assembly of NuRD complexes that regulate the transcription of genes necessary for neural progenitor proliferation, radial migration, and cortical layer specification.

RESULTS

NuRD Is the Major Nuclear Complex Associated with HDAC2 in the Developing Cortex

Recent work from our lab (Nott et al., 2008, 2013) and others (Hagelkruys et al., 2014, 2015) demonstrated that the class I histone deacetylases HDAC1 and HDAC2 control the expression of genes necessary for neuronal development. Class I HDACs do not bind DNA directly and are found within large multiprotein complexes that confer target specificity (Haberland et al., 2009). To identify HDAC2-containing complexes in the embryonic mouse brain, we performed mass spectrometry of proteins that co-immunoprecipitate with HDAC2 during cortical development. Cortices were dissected at embryonic day 12.5 (E12.5), E15.5, and E18.5, and cell lysates were incubated with HDAC2 antibody or normal immunoglobulin G (IgG). Immunoprecipitated proteins were proteolytically digested and analyzed by mass spectrometry (Figure S1A). The relative abundance of identified proteins was calculated using normalized spectra abundance factors (NSAFs). Proteins enriched in HDAC2 immunoprecipitates are listed in Figure S1B and Table S1. Strikingly, all known subunits of the NuRD complex were associated with HDAC2 at all embryonic stages (Figure S1B). Subunits of the CoREST (REST corepressor 1) and mSin3 complexes were also detected (Figure S1B). HDAC2 co-immunoprecipitation followed by western blot analysis of CHD3, CHD4, CHD5, MTA1, MTA2, and Rbbp7 confirmed the findings of mass spectrometry analysis and revealed that the association of CHDs with HDAC2 changed according to developmental stage (Figures 1A and 1B). While CHD4 co-immunoprecipitated with HDAC2 at E12.5 and E15.5, CHD3 and CHD5 were not detected at E12.5 and became part of the NuRD complex at later stages of cortical development. CHD3 co-immunoprecipitated with HDAC2 at both E15.5 and E18.5, whereas CHD5 was detected predominantly at E18.5. Importantly, co-immunoprecipitation experiments confirmed mutually exclusive occupancy of CHD3, CHD4, and CHD5 within NuRD complexes (Figure 1C). Glycerol gradient analysis performed on E15.5 cortices indicated that 78% of CHDs co-sediment as a complex in fractions that also contain the NuRD subunits MTA1, MTA2, and Mbd3 (Figures 1D and S1C). Taken together, these results indicate that the composition of NuRD complex changes during cortical development and that CHD3, CHD4, and CHD5 occupancy within the complex is mutually exclusive.

CHDs Are Developmentally Regulated in the Cortex

The mouse cortex is formed between E11 and E18 in a characteristic inside-out manner, with deep layers (layers IV–VI) generated first and more superficial layers (layers II and III) generated later (Florio and Huttner, 2014). Neurons that will eventually occupy the external layers of the cortex must migrate through the deeper layers in order to reach their final location. During migration and laminar formation, neurons mostly maintain the molecular properties acquired at early stages, although their laminar identity is further specified by postmitotic factors that contribute to the establishment of mature neuronal identity (Kwan et al., 2012). To investigate the expression of CHDs at different stages of cortical development, mouse cortices were dissected at E12.5, E15.5, and E18.5 and subjected to qRT-PCR and western blot analyses (Figures S2A and S2B). At E12.5, CHD3 and CHD5 were expressed at relatively low levels that increased at E15.5 and E18.5. In contrast, CHD4 expression remained constant throughout development. Immunostaining of coronal sections confirmed that CHD4 was the only CHD detected at E12.5, with strong expression observed in NPCs (Figure S2C). At later stages (E15.5 and E18.5), CHD3 and CHD5 were also expressed and found in differentiated neurons of the nascent cortical plate (CP), where they co-localized with the deep layer neuronal marker Ctip2 (Figures S2C and S3). A similar expression pattern was observed when neural progenitors isolated from E14.5 rat cortices were differentiated in vitro and immunostained for CHDs and either nestin, a marker of NPCs, or the neuronal marker MAP2 (Figures S4A and S4B). Co-immunoprecipitation experiments confirmed that HDAC2 and the core NuRD subunit MTA2 mostly interact with CHD4 in NPCs, whereas differentiation correlates with a decline in CHD4 interaction and association of both CHD3 and CHD5 (Figures S4C and S4D). Thus, the ATPase activity of NuRD is mostly mediated by CHD4 in NPCs, whereas this function is largely provided by CHD3 and CHD5 at later differentiation stages. Given that the occupancy of CHDs within the NuRD complex is mutually exclusive (Figure 1C), a possible interpretation of these findings is that the coexistence of distinct NuRD complexes within neurons may be necessary to regulate diverse molecular functions.

Deletion of CHD4 Causes Premature Cell-Cycle Exit of NPCs and Depletion of Intermediate Progenitor Cells

To investigate the role of CHDs during cortical development, we first analyzed the cortex of mice carrying a conditional mutation of the CHD4 gene (Williams et al., 2004). CHD4 floxed mice were crossed with transgenic mice carrying CRE recombinase under the control of the nestin promoter, which results in deletion of CHD4 in the nervous system from early embryonic stages (Dubois et al., 2006). Most mice lacking CHD4 (CHD4$^{fl/fl}$/nestin-CRE) died at birth and at E18.5 the brain was significantly smaller than control littermates (CHD4$^{fl/fl}$ and CHD4$^{WT/WT}$/nestin-CRE; Figure 2A), showing remarkable reduction of cortical thickness (Figure 2B). Although TUNEL staining performed at both E13.5 and E16.5 showed no increase in cell death in CHD4$^{fl/fl}$/nestin-CRE cortices compared to control littermates (Figures 2C and 2D), we observed a reduction of the number of cells expressing the proliferation marker Ki67 (Figures 2F and 2G). EdU (5-ethynyl-2’-deoxyuridine) labeling coupled with Ki-67 immunostaining revealed that in CHD4$^{fl/fl}$/nestin-CRE cortices, NPCs prematurely exited the cell cycle (Figures 2F and 2G), and this event was associated with increased apoptosis at E18.5 (Figure 2E). Thus, in cortices lacking CHD4, a proportion of NPCs precociously exit the cell cycle, fail to differentiate, and die.

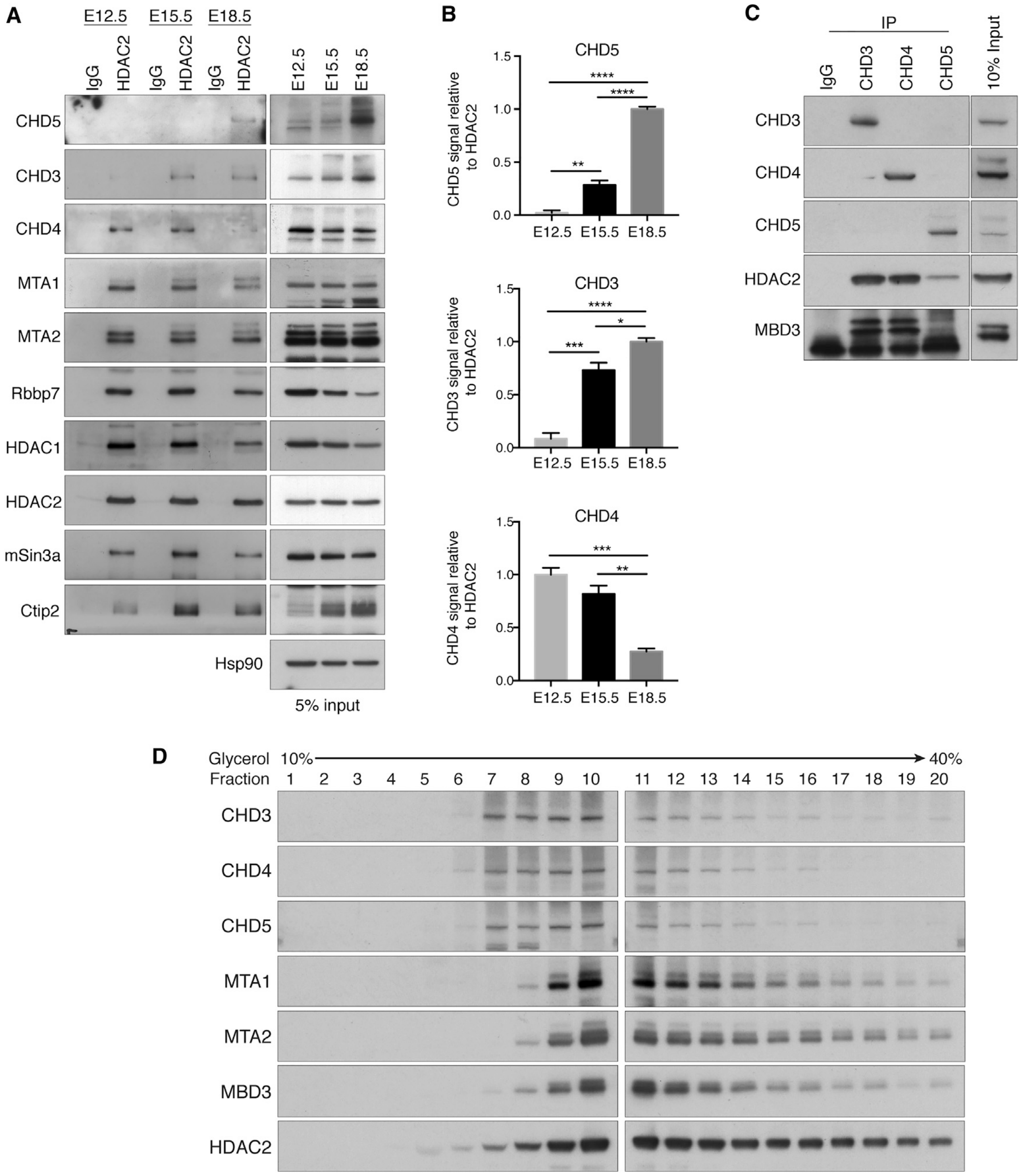

Figure 1. Characterization of NuRD Complexes in the Developing Cortex

(A) Lysates of cortices were subjected to co-immunoprecipitation with HDAC2 antibody followed by immunoblotting for NuRD subunits. Representative western blot; n = 3.

(B) Densitometry analysis of CHDs co-immunoprecipitating with HDAC2 in E12.5, E15.5, and E18.5 cortex. Data are presented as mean ± SEM of three independent experiments. *$p < 0.05$, **$p < 0.01$, ***$p < 0.001$, and ****$p < 0.0001$ by one way ANOVA with Tukey's multiple comparisons test.

(C) Lysates of E15.5 cortices were subjected to co-immunoprecipitation with CHD3, CHD4 or CHD5 antibodies followed by immunoblotting for HDAC2 and MBD3. Representative blot; n = 3.

(D) Glycerol gradient co-sedimentation analysis of nuclear extracts from E15.5 cortices. Representative blot; n = 3.

See also Figures S1–S4.

Progenitor cells, such as radial glia (RG) and apical progenitors (APs), are located in the VZ and express the transcription factors Pax6 and Sox2 (Götz et al., 1998; Graham et al., 2003). To study whether deletion of CHD4 affected NPC number, cortices of CHD4$^{fl/fl}$/nestin-CRE mice and control littermates were immunostained for Pax6 and Sox2. At E13.5 and E16.5, the number of

OPEN
ACCESS
CellPress

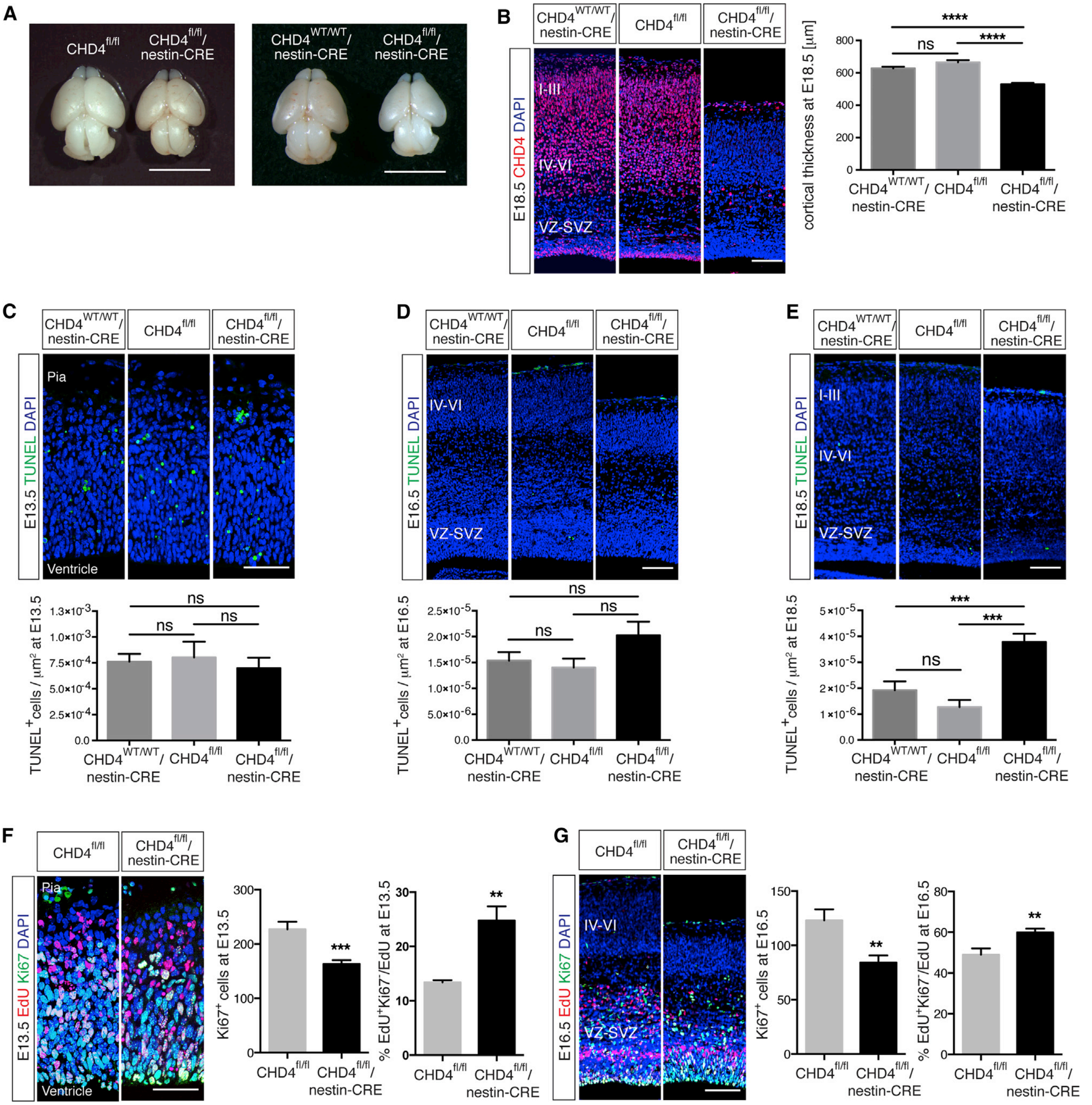

Figure 2. Deletion of CHD4 Causes Microcephaly and Premature Cell-Cycle Exit of NPCs

(A) CHD4$^{fl/fl}$/nestin-CRE and control (CHD4$^{fl/fl}$ and CHD4$^{WT/WT}$/nestin-CRE) brains dissected at E18.5. Scale bar, 5 mm.

(B) Left: coronal sections of CHD4$^{fl/fl}$/nestin-CRE and control brains at E18.5 immunolabeled for CHD4 (red). Scale bar, 100 μm. Right: cortical thickness in E18.5 embryos, measured across the cortical wall using coronal sections; five to ten embryos obtained from four litters were analyzed per genotype.

(C) Top: TUNEL staining of CHD4$^{fl/fl}$/nestin-CRE cortex at E13.5. Scale bar, 50 μm. Bottom: number of TUNEL-positive cells per 1 μm^2 of cortical area. Four to nine embryos obtained from four litters were analyzed per genotype.

(D) Top: TUNEL analysis of CHD4$^{WT/WT}$/nestin-CRE, CHD4$^{fl/fl}$ and CHD4$^{fl/fl}$/nestin-CRE cortices at E16.5. Scale bar, 100 μm. Bottom: number of TUNEL-positive cells per 1 μm^2 of cortical area. 5–12 embryos obtained from five litters were analyzed per genotype.

(E) Top: TUNEL analysis of CHD4$^{fl/fl}$/nestin-CRE cortex at E18.5. Scale bar, 100 μm. Bottom: number of TUNEL-positive cells per 1 μm^2 of cortical area. Five to ten embryos obtained from four litters were analyzed per genotype.

(legend continued on next page)

cells expressing Pax6 in the VZ was unchanged (Figures 3A and 3B), whereas at E16.5, the number of Sox2-expressing cells was slightly increased (Figure 3C). However, when Pax6 and Sox2 immunostaining were combined with a 2 hr EdU pulse, it revealed a significant reduction in the number of actively proliferating apical progenitors in CHD4$^{fl/fl}$/nestin-CRE mice (Figures 3B and 3C). RG and APs divide at the apical surface and give rise to neurons as well as another neuronal progenitor cell type called intermediate progenitor cells (IPCs, also known as basal progenitors) (Noctor et al., 2004). IPCs divide in a basal position within the VZ and subventricular zone (SVZ) and express the transcription factor Tbr2 (also known as EOMES) (Englund et al., 2005). In lissencephalic rodents, IPCs represent the principal neurogenic cells and are considered to be responsible for the amplification of neuronal cell number that drove cortical expansion during evolution (Martínez-Cerdeño et al., 2006). To investigate whether CHD4 influences IPC number, cortices of CHD4$^{fl/fl}$/nestin-CRE mice were immunostained for Tbr2 and analyzed using confocal microscopy. In the absence of CHD4, the number of Tbr2-expressing cells was reduced at E13.5 and E16.5 (Figures 3A and 3D), and EdU pulse experiments demonstrated a striking reduction of proliferating Tbr2-positive IPCs at E16.5 (Figure 3D). Because symmetric division of IPCs is necessary for the expansion of neuronal output their depletion may account for the cortical thinness observed in CHD4$^{fl/fl}$/nestin-CRE mice.

Deletion of CHD4 Alters Cortical Lamination

To ask how IPC depletion affected the formation of cortical layers we analyzed a number of markers specific for either upper (II and III) or deeper (IV–VI) layer neurons. E18.5 CHD4$^{fl/fl}$/nestin-CRE and control cortices were immunostained for upper (SATB2 and Cux1) (Alcamo et al., 2008; Britanova et al., 2008; Nieto et al., 2004) or deeper (Tbr1 and Ctip2) (Arlotta et al., 2005; Hevner et al., 2001) layer-specific markers. In the absence of CHD4, the number of neurons expressing Tbr1 and Ctip2 was unchanged (Figures 3E and 3F). In contrast, upper layer thickness and the number of neurons expressing SATB2 and Cux1 were greatly reduced (Figures 3E and 3F). Although IPCs generate neurons that populate all cortical layers (Sessa et al., 2008), they are the most abundant cell type in the SVZ, which develops between E13 and E15, at a time when deeper layer neurons are already generated. Thus, in CHD4$^{fl/fl}$/nestin-CRE mice IPC depletion in the SVZ severely and specifically affects the formation of the upper layers of the cortex.

CHD3, CHD4, and CHD5 Regulate Distinct Aspects of Neural Radial Migration

Contrary to CHD4, CHD3, and CHD5 expression is very low in neural progenitors and increases steadily during the late stages of neurogenesis, when neurons migrate radially to populate the CP (Figure S2). In the developing cortex, CHD3 and CHD5 show similar, albeit not entirely overlapping, expression profiles. CHD3 is expressed in neurons that have reached the CP, whereas CHD5 was also detected in the SVZ (Figure S2C). To ask whether CHD3 and CHD5 influence specific aspects of neuronal differentiation and radial migration, we employed the in utero electroporation technique. We tested the suitability of this approach by comparing the defects observed in the cortex of CHD4$^{fl/fl}$/nestin-CRE mice with CHD4$^{fl/fl}$ embryos subjected to in utero electroporation using either empty vector (EV) or CRE-containing vector. Brains were electroporated at E14.5 and Tbr2 expression was analyzed 24 hr later. Electroporation with the CRE vector resulted in a striking reduction of Tbr2-positive cells comparable to CHD4$^{fl/fl}$/nestin-CRE mice (Figures S5A and 3D). Similarly, electroporation of E14.5 brains with a CHD4-specific small hairpin RNA (shRNA) decreased both NPC proliferation and the number of IPCs (Figures S5B–S5D) compared to control shRNA (shCTL). Next, we performed in utero electroporation at E13.5 using shRNA targeting either CHD5 or CHD3 (shCHD5 and shCHD3, respectively), shCTL, or an EV (Figure 4). Potential non-specific effects were ruled out by co-electroporation of rescue vectors encoding human CHD5 and CHD3. Five days after electroporation, cortices were dissected and neural migration was assessed by counting the number of GFP expressing cells that had reached the CP. NPCs are electroporated in the VZ, exit the cell cycle and migrate radially crossing the SVZ and IZ to reach their final position in the CP. Most neurons electroporated with shCHD5 accumulated within the IZ and failed to reach the CP, whereas these defects were absent in brains electroporated with EV or shCTL and were completely rescued by co-electroporation with hCHD5 (Figure 4A). Newly generated postmitotic neurons are initially multipolar and transiently grow a variable number of immature neurites. This phase precedes the establishment of neuronal polarity that is essential for radial migration (Barnes and Polleux, 2009). To test whether abnormal neuronal polarization was responsible for the defects of radial migration, we analyzed the morphology of postmitotic neurons within the SVZ of embryos electroporated with shCTL or shCHD5. Lack of CHD5 resulted in the accumulation of multipolar neurons within the IZ (Figure 4B), suggesting that CHD5 contributes to the establishment of neuronal polarity.

Because CHD3 is confined to postmitotic, differentiated neurons (Figures S2C and S4B), we reasoned that it may influence the late stages of cortical development. E13.5 embryos were electroporated with EV, shCTL, shCHD3, or shCHD3 + hCHD3 and after 5 days, cortices were immunostained for GFP and CHD3. Knockdown of CHD3 induced a delay of neuronal migration, with a significant number of cells retained in the deeper layers (IV–VI) and fewer neurons reaching the upper layers (II and III) of the cortex (Figure 4C). Similarly to CHD5, multipolar cells accumulated in the deeper layers

(F) Analysis of cell-cycle exit index of CHD4$^{fl/fl}$/nestin-CRE cortices at E13.5. EdU in vivo labeling at E12.5 followed by analysis of Ki67 expression at E13.5. 5–11 embryos obtained from five litters were analyzed per genotype. Scale bar, 50 μm.

(G) Analysis of Ki67-positive cells and cell-cycle exit index of the CHD4$^{fl/fl}$/nestin-CRE cortex at E16.5 performed as in (F). Scale bar, 100 μm.

All data are presented as mean ± SEM. **p < 0.01 and ***p < 0.001 (ns, not significant) by one-way ANOVA with Tukey's multiple comparisons test (B–E) or unpaired t test (F and G).

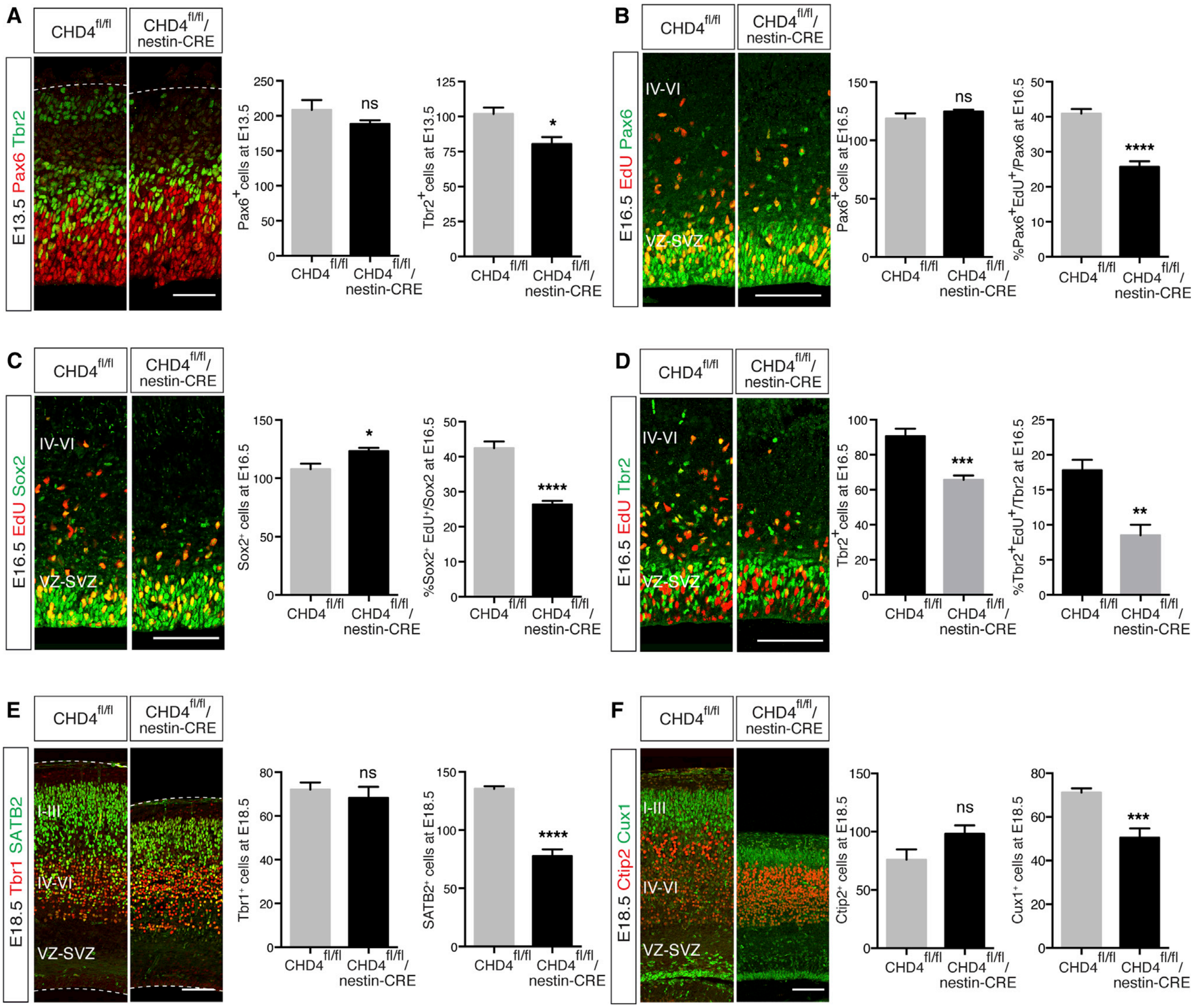

Figure 3. Deletion of CHD4 Causes IPCs Depletion and Defects of Cortical Lamination

(A) Left: coronal sections of E13.5 CHD4$^{fl/fl}$/nestin-CRE and CHD4$^{fl/fl}$ control brains immunostained for Pax6 (red) and Tbr2 (green). Scale bar, 50 μm. Right: quantification of Pax6- and Tbr2-expressing cells per section. 5–12 embryos obtained from five litters were analyzed per genotype.

(B–D) Left: coronal sections of E16.5 CHD4$^{fl/fl}$/nestin-CRE and control brains harvested 2 hr after EdU injection and immunolabeled with EdU (red) and Pax6 (B), Sox2 (C), or Tbr2 (D) (green). Scale bar, 100 μm. Right: number of cells expressing Pax6 (B), Sox2 (C), and Tbr2 (D) per section and percentage of cells co-labeled with EdU. Five to six embryos obtained from three litters were analyzed per genotype.

(E and F) Left: coronal sections of E18.5 CHD4$^{fl/fl}$/nestin-CRE and CHD4$^{fl/fl}$ control brains immunolabeled with Tbr1 (red) and SATB2 (green) (E) or Ctip2 (red) and Cux1 (green) (F). Scale bar, 100 μm. Right: quantification of cells expressing Tbr1 and SATB2 (E) or Ctip2 and Cux1 (F) in each section. Littermates were obtained from five litters, and five to ten embryos were analyzed per genotype.

All data are presented as mean ± SEM. *p < 0.05, **p < 0.01, ***p < 0.001, and ****p < 0.0001 (ns, not significant) by unpaired t test. See also Figure S5.

of the CP (Figure 4D), suggesting that also in this case abnormalities of neuronal polarity may contribute to radial neural migration defects. When brains of CHD4$^{fl/fl}$ mice were electroporated with EV or vector expressing CRE, radial migration and neuronal polarity were not affected (Figure S5E). These findings show that CHD3, CHD4, and CHD5 have non-overlapping roles during cortical development and further support the hypothesis that they are part of distinct NuRD complexes.

CHD3 and CHD5 Differentially Regulate Cortical Layer Specification

Neuronal differentiation, radial migration and laminar identity are known to be co-regulated processes (Kwan et al., 2012). It is therefore possible that CHD5 and CHD3 influence the transcription of genes that control both neural migration and layer positioning. We first investigated whether the early defects of neural radial migration in neurons lacking CHD5 depended on abnormal differentiation of NPCs. Brains electroporated with EV, shCTL,

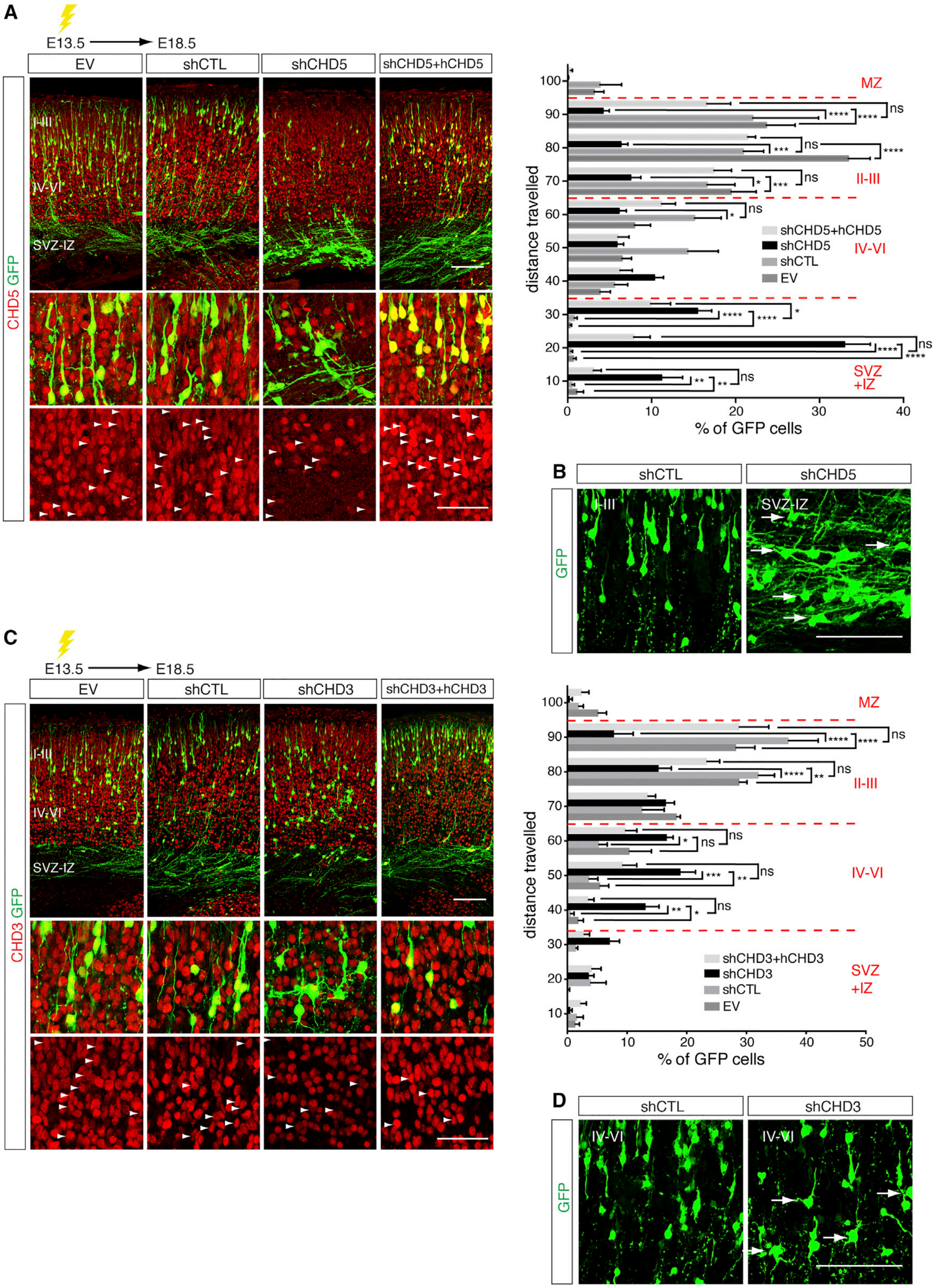

Figure 4. CHD5 and CHD3 Regulate Distinct Aspects of Neural Radial Migration

(A) Left: E13.5 embryos were in utero electroporated with the indicated shRNA-GFP vectors, and electroporated cells (green) expressing CHD5 (red) were analyzed at E18.5. Scale bar, 100 μm. Bottom: higher-magnification images of the sections. Arrowheads indicate electroporated cells. Scale bar, 50 μm.

(legend continued on next page)

shCHD5, or shCHD5 + hCHD5 were immunostained for the neural progenitor markers Pax6 and Ki67. In contrast to published data (Egan et al., 2013), we did not find that knockdown of CHD5 caused persistent expression of progenitor markers (Figures S5F and S5G). Instead, in brains electroporated with shCHD5, a remarkable number of neurons that did not migrate toward the CP expressed the upper layer markers Cux1 and SATB2 (Figures 5A and 5B). Because these neurons never reach layers II to III, they give rise to an ectopic layer located beneath the CP.

We next asked whether the defects of late neural radial migration caused by inhibition of CHD3 are coupled with abnormalities of laminar identity. E13.5 embryos were electroporated with shCHD3 or control vectors and after 5 days, brains were immunostained for the layer-specific markers Tbr1, Sox5, Brn2, and Cux1. Neurons lacking CHD3 were more likely to express Tbr1 and Sox5 (Figures 5C and 5D), which are transcription factors that regulate laminar positioning and differentiation of deeper cortical layers (Hevner et al., 2001; Lai et al., 2008), whereas a lower number of neurons expressed the upper layer markers Brn2 and Cux1 (Dominguez et al., 2012; Nieto et al., 2004) (Figures 5E and 5F). Thus, despite being largely co-expressed, deletion of CHD5 or CHD3 results in strikingly different cortical defects. CHD5 is necessary during early radial migration and does not affect the expression of laminar-specific markers. In contrast, CHD3 promotes late neural radial migration and layer specification, implicating that it may influence the expression of genes that couple radial migration with laminar identity.

Non-Redundant Functions of CHD3, CHD4, and CHD5 during Cortical Development

We reasoned that if CHD3, CHD4, and CHD5 regulate non-overlapping aspects of cortical development, the abnormalities observed in brains lacking one of them should not be reverted by co-expression of a rescue vector encoding another. First, we confirmed that mCHD4, hCHD3, and hCHD5 rescue constructs were expressed in cells transfected with shCHD3 or shCHD5. Overexpressed CHDs were easily detected in postmitotic neurons depleted of either CHD5 or CHD3 (Figures 4A, 4C, and S6A–S6C) and were incorporated within NuRD complexes under these conditions (Figure S6D). We used cortices depleted of CHD3 or CHD5, because these proteins are more likely to be functionally redundant due to a similar pattern of expression (Figure S2C). In brains co-electroporated with shCHD5 and either hCHD3 or mCHD4, the number of neurons that reached the CP (Figures 6A, 6C, and 6D) and ectopic expression of the upper layer markers SATB2 and Cux1 (Figures 6A and 6B) were comparable to neurons electroporated with shCHD5 alone. When similar experiments were performed in brains electroporated with shCHD3, expression of mCHD4 did not rescue the neural radial migration defects (Figures 6E and 6G) or change the number of cells expressing Cux1 and Tbr1 (Figures 6E and 6F). Moreover, co-electroporation of shCHD3 and hCHD5 did not influence the number of neurons expressing Cux1 or Tbr1 (Figures 6E and 6F), whereas radial migration defects were rescued under these conditions (Figures 6E and 6H), suggesting that CHD5 may provide functional compensation in regards to neuronal migration. Thus, CHD3, CHD4, and CHD5 have distinct and mostly non-redundant functions during cortical development that depend on their mutually exclusive inclusion within NuRD complexes.

CHD Subunits Are Recruited to Distinct Gene Promoters

First, we analyzed the transcriptional profile of the developing cortex by performing microarray analysis of mRNA purified from E12.5, E15.5, or E18.5 cortices (mouse WG-6 v2.0 expression BeadChip Illumina, n = 4 for condition). 30,869 transcripts were identified, 3,627 of which (corresponding to 2,835 genes) were differentially expressed during development (fold change of ±2 coupled with a false discovery rate [FDR] < 0.01) (Figures S7A and S7B; Table S2). The transcription factors Sox2, Pax6, and Tbr2 were of particular interest, as they were developmentally regulated (Figure S7C). Sox2 and Pax6 are both expressed in NPCs; Sox2 maintains apical progenitors in a proliferative state (Graham et al., 2003), and Pax6 regulates cell-cycle length of apical progenitors and IPC specification (Englund et al., 2005). Tbr2 is necessary for IPC proliferation and neurogenesis (Arnold et al., 2008), and ablation of either Pax6 or Tbr2 results in a substantial loss of cortical progenitors (Quinn et al., 2007; Sessa et al., 2008). To avoid potential confounding issues due to the cellular heterogeneity of the cortex, experiments were performed on NPCs maintained in vitro for 2 days or cultured in differentiating conditions for 7 days (postmitotic neurons [PMNs]) (Figure S4A). Binding of CHD subunits to *Sox2*, *Pax6*, and *Tbr2* promoters in NPCs and PMNs was assessed by chromatin immunoprecipitation (ChIP) assay. CHD4 was bound to *Sox2*, *Pax6*, and *Tbr2* gene promoters at much greater levels in NPCs than in PMNs (Figure 7A). Conversely, the recruitment of CHD3 to the same regions was higher in PMNs than in NPCs, whereas CHD5 binding remained unchanged. The switch of CHD4 to CHD3 binding correlated with transcriptional inhibition, suggesting that, at least for these genes, the role of NuRD complexes on gene expression may depend on the incorporation of specific CHD subunits. As expected, levels of Sox2, Pax6, and Tbr2 were remarkably reduced in NPCs obtained from CHD4 null mice (Figure 7B). To identify putative transcription factors

Right: quantification of electroporated cells traveling the distance between ventricular surface (0) and pial surface (100). 5–13 embryos were analyzed per condition; n = 3.

(B) Representative images of neurons within the layers II–III or SVZ-IZ electroporated with shRNA-GFP vectors. Arrows indicate multipolar neurons. Scale bar, 100 μm.

(C) Left: E13.5 embryos were in utero electroporated with shRNA-GFP vectors and CHD3 expression (red) in electroporated cells was analyzed at E18.5. Scale bar, 100 μm. Bottom: higher-magnification images of the sections. Arrowheads indicate electroporated cells. Scale bar, 50 μm. Right: distribution of electroporated cells quantified as in (A). 5–11 embryos were analyzed per condition; n = 3.

(D) Representative images of layers IV–VI neurons electroporated with shRNA-GFP vectors. Arrows indicate multipolar neurons. Scale bar, 100 μm.

All data are presented as mean ± SEM. *p < 0.05, **p < 0.01, ***p < 0.001, and ****p < 0.0001 (ns, not significant) by two-way ANOVA with Tukey's multiple comparisons test.

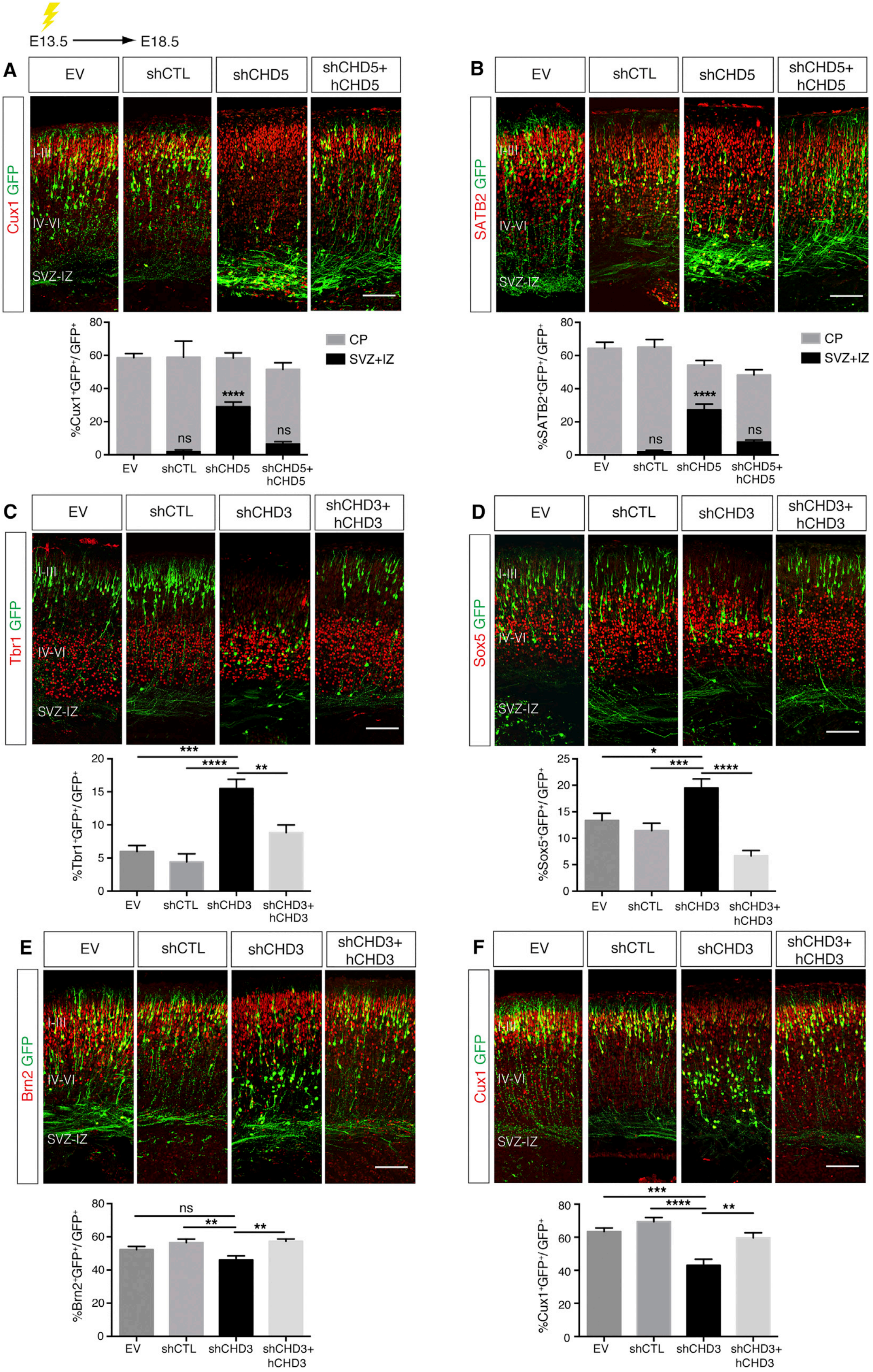

(legend on next page)

that may be involved in the recruitment of CHD4-containing NuRD complexes to target genes, we investigated whether Sox2 was bound to promoters occupied by CHD4. Sox2 represented an interesting candidate, as it interacts with CHD4 in neural stem cells (Engelen et al., 2011). ChIP experiments demonstrated that similar to CHD4, Sox2 was recruited to *Sox2*, *Pax6*, and *Tbr2* promoters in NPCs, and binding was significantly reduced in PMNs (Figure 7C). Strikingly, ectopic expression of hCHD3 at E13.5 had an effect similar to ablation of CHD4 and caused a reduction of Pax6, Sox2, and Tbr2 expression in NPCs (Figure 7D), indicating that the composition of NuRD complexes may represent a mechanism by which Pax6, Sox2, and Tbr2 expression is developmentally regulated.

CHD5 and CHD3 binding was tested on the promoters of doublecortin (Dcx) and apolipoprotein E receptor 2 (ApoER2), two genes that regulate neural radial migration and cortical lamination (Francis et al., 1999; Gleeson et al., 1999; Trommsdorff et al., 1999). Enrichment of CHD5 was detected on both promoters in PMNs (Figure 7E), whereas CHD3 binding was unchanged in NPCs and PMNs. A similar result was observed when the promoter of *RhoA*, a gene that regulates numerous aspects of neuronal migration in the cortex (Cappello, 2013), was analyzed (Figure 7E). Moreover, electroporation of shCHD5, but not shCHD3, reduced Dcx and RhoA levels in migrating cortical neurons (Figure 7F). Thus, CHD3 and CHD5 exhibit specificity in regards to both recruitment to target genes and transcriptional regulation at these regions.

DISCUSSION

During brain development, chromatin remodeling is essential for the expression of genes that regulate the differentiation of pluripotent cells into mature neurons. A number of components of the BAF (Brg1- and Brm-associated factors) complex, for example, undergo subunit switch during neuronal differentiation, generating unique complexes that activate specific transcriptional programs (Yoo and Crabtree, 2009). In this study, we show that NuRD represents a major ATP-dependent chromatin remodeling complex in the developing mouse brain. NuRD has been increasingly linked with transcriptional regulation of genes necessary for cell differentiation and growth (Basta and Rauchman, 2015). In addition to transcriptional repression and silencing, NuRD has more complex effects on gene expression, including transcriptional activation, regulation of enhancers, and inactivation of activity-dependent genes (Shimbo et al., 2013; Yang et al., 2016). One important function of NuRD is to maintain progenitor cell populations and to inhibit the expression of pluripotency and Polycomb-regulated genes (Egan et al., 2013; Reynolds et al., 2012a, 2012b). At least nine CHDs have been identified so far, and several have been implicated in brain functions (Basta and Rauchman, 2015). CHD4, for example, induces presynaptic differentiation of cerebellar granule neurons and regulates the transcription of genes necessary for establishing synaptic connectivity and neurotransmission in Purkinje cells (Yamada et al., 2014). CHD4 has also been shown to be responsible for the inactivation of activity-dependent genes in the cerebellum, by promoting deposition of the histone variant H2A.z at these loci (Yang et al., 2016).

Most CHDs are expressed in neurons (Micucci et al., 2015); however, mass spectrometry analysis indicated that only CHD3, CHD4, and CHD5 are associated with NuRD in the embryonic cortex (Figures 1 and S1). Knockdown of CHD5 in the developing cortex induces severe defects of neural radial migration (Egan et al., 2013). In cortical neurons, depletion of CHD5 alters the expression of neuron-specific genes, transcription factors, and, surprisingly, the BAF45b subunit of the BAF complex (Potts et al., 2011), perhaps suggesting a functional link between two major chromatin remodeling complexes. We found that in the brain, CHD3, CHD4, and CHD5 undergo a developmentally regulated subunit exchange that results in the assembly of stage-specific NuRD complexes (Figure 1A). Importantly, deletion or inhibition of each CHD has distinct effects on cortical development. Conditional deletion of CHD4 induced premature cell-cycle exit of NPCs (Figures 2F and 2G) that led to a depletion of IPCs (Figure 3D) and may have contributed to the reduced size of the cortex observed in CHD4$^{fl/fl}$/nestin-CRE mice (Figure 2A). Interestingly, CHD3, CHD4, and CHD5 are all detected in postmitotic neurons (Figure S3), yet they exert distinct and mostly non-overlapping functions. Lack of CHD5 affects the early stages of neural migration (Figure 4A) and induces ectopic expression of the upper layer markers Cux1 and SATB2 (Figures 5A and 5B), whereas CHD3 is necessary for the late stages of neural radial migration (Figure 4C) and laminar specification (Figures 5C–5F). Rescue experiments using human and mouse CHDs showed little functional redundancy among CHD3, CHD4, and CHD5 (Figure 6), indicating that intracellular expression of a CHD protein in and of itself is not sufficient to compensate for the specific developmental defects.

HDAC2 co-immunoprecipitated with all NuRD subunits; however, it should be noted that loss of HDAC2 is not expected to resemble the defects observed in cortices lacking CHD3, CHD4, or CHD5. HDAC2 is part of many nuclear complexes; therefore, its effect on gene expression is not limited to its inclusion within NuRD. Moreover, there is a significant functional redundancy between HDAC1 and HDAC2, perhaps even within the NuRD complex, which can recruit both deacetylases (Montgomery et al., 2009). In contrast, we found little functional compensation among CHD3, CHD4, and CHD5 (Figure 6);

Figure 5. CHD3 and CHD5 Regulate Cortical Layer Specification

(A and B) Cortical neurons were in utero electroporated with shRNA-GFP vectors and immunolabeled for GFP (green) and Cux1 (red) (A) or SATB2 (red) (B). Percentage of GFP-positive cells expressing Cux1 (A) or SATB2 (B) present in SVZ + IZ or CP. 6–13 embryos were analyzed per condition; n = 3. Scale bar, 100 μm.

(C–F) Cortical neurons were in utero electroporated with shRNA-GFP vectors and immunolabeled for GFP (green) and Tbr1 (red) (C), Sox5 (red) (D), Brn2 (red) (E), or Cux1 (red) (F). The percentage of co-labeled GFP-positive cells was quantified. Scale bar, 100 μm. 3–15 embryos were analyzed per condition; n = 3.

All data are presented as mean ± SEM. *p < 0.05, **p < 0.01, ***p < 0.001, and ****p < 0.0001 (ns, not significant) by two way (A) and (B) or one-way ANOVA (C–F) with Tukey's multiple comparisons test. See also Figure S5.

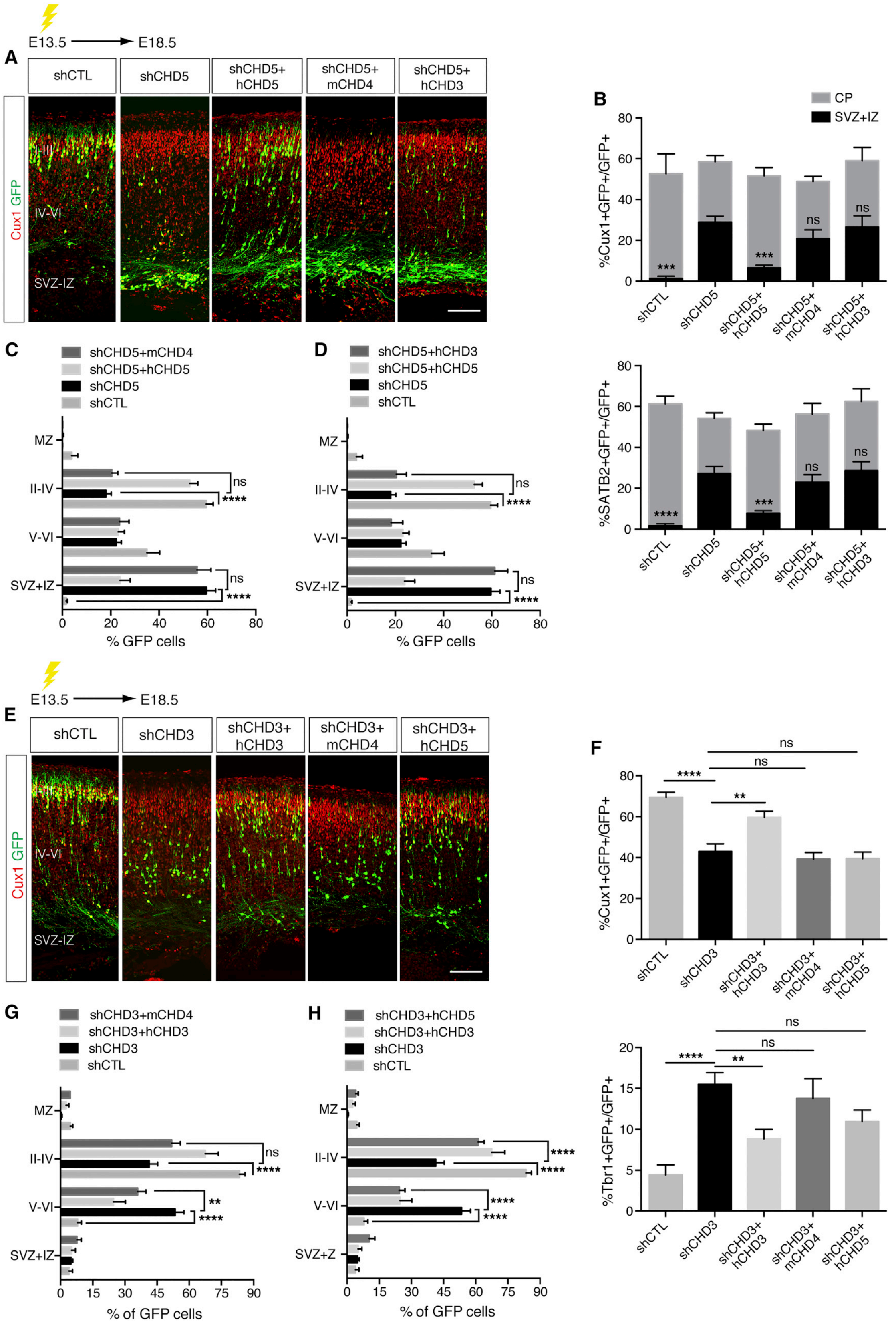

(legend on next page)

thus, inhibition of only one CHD is expected to induce defects that are distinct from loss of HDAC1 and/or HDAC2. Analysis of the remaining NuRD core subunits indicated that, in many instances, their inclusion within NuRD complexes is also developmentally regulated (Figure S1) and may undergo developmental switch. Of note, deletion of MBD3 results in cortical defects strikingly similar to the abnormalities observed in brains lacking CHD4 (Knock et al., 2015), suggesting that MBD3 and CHD4 may be part of NuRD complexes that regulate genes necessary for determining IPC number and upper layer specification. The majority (78%) of NuRD subunits co-sediment as a complex (Figures 1D and S1C); however, it is possible that at least some cortical defects depend on CHDs acting independently. CHD4, for example, interacts with the histone acetyl transferase p300 in thymocytes (Williams et al., 2004) and regulates the expression of the γ-globin gene in an MBD2-independent manner (Amaya et al., 2013). Further investigation will be needed to define whether there is an NuRD-independent role for CHDs during cortical development.

How do NuRD complexes containing different CHDs regulate the expression of specific genes? Chromatin remodeling factors usually lack sequence-specific DNA-binding ability; therefore, NuRD complexes may interact with transcription factors that mediate their targeting to regulatory elements, such as gene promoters and enhancers. In lymphocytes, NuRD interacts with the transcription factor Ikaros, which mediates NuRD recruitment to genes necessary for lymphoid differentiation (Zhang et al., 2011), and in neuroblastoma cell lines, NuRD complexes associate with Ctip2 (Topark-Ngarm et al., 2006). We found that in NPCs, the transcription factor Sox2 is recruited to promoters of CHD4-target genes and that this binding decreases upon differentiation to PMNs (Figure 7C). Since CHD4 and Sox2 interact in ESCs (Engelen et al., 2011), this represents a potential mechanism through which CHD4-containing NuRD complexes may be recruited to specific genomic loci. In addition, post-translational modifications of NuRD subunits may also influence the composition of NuRD complexes and their recruitment to chromatin.

Finally, our study highlights the potential role of NuRD complexes in establishing cortical connectivity. Mutations in a number of ATP-dependent chromatin remodeling enzymes have been associated with autism spectrum disorders, intellectual disability, and epilepsy (Neale et al., 2012; O'Roak et al., 2012; Allen et al., 2013). Further studies will be necessary to determine the role of ATP-dependent chromatin remodeling enzymes during the establishment of neuronal circuitry in early brain development and how alteration of their function contributes to neurodevelopmental defects.

EXPERIMENTAL PROCEDURES

Animals

All animal experiments were approved by the UCL Animal Welfare and Ethical Review Body and carried out in accordance to appropriate UK Home Office licenses.

WT, nestin-CRE and CHD4$^{fl/fl}$ conditional knockout mice were of C57BL/6J background. Nestin-CRE heterozygous mice were purchased from Jackson Laboratory (strain B6.Cg(SJL)-TgN(NesCre)1Kln). CHD4 homozygous mice were crossed with nestin-CRE heterozygous mice to obtain CHD4$^{fl/+}$/nestin-CRE offspring. CHD4$^{fl/+}$/nestin-CRE heterozygous mice were intercrossed with either CHD4$^{fl/+}$/nestin-CRE heterozygous or CHD4$^{fl/fl}$ homozygous mice to generate CHD4 null and control littermates.

Whole-Sample Mass Spectrometry

HDAC2-containing complexes were immunoprecipitated with HDAC2 antibody, and immune complexes were eluted from the beads in elution buffer containing 0.5% Progenta anionic acid labile surfactant I (AALS I) (Protea). Samples were digested by the sequential addition of lys-C and trypsin proteases, desalted using C-18 StageTips, and fractionated online using microscale C18 reverse-phase chromatography as previously described (Wohlschlegel, 2009). Tandem mass spectrometry (MS/MS) spectra were acquired in a data-dependent manner on a Q-Exactive mass spectrometer (Thermo Fisher Scientific). Peptide identifications were generated from MS/MS spectra by searching the UniProt SwissProt (May 2010 release) protein database using the ProLuCID algorithm. Peptide identifications were filtered using DTASelect and required at least two peptides per protein and a decoy-database estimated false-positive rate of less than 5% (Elias and Gygi, 2007). NSAF values were calculated for each protein in order to determine the approximate degree of enrichment of putative HDAC2-interacting proteins (Florens et al., 2006).

Nuclear Protein Extraction and Glycerol Gradient Co-sedimentation

Nuclear protein extraction from E15.5 cortices was carried out according to a protocol obtained from the Crabtree lab (http://crablab.stanford.edu/Protocolsneuronextra.htm). Extracts were run on 10%–40% glycerol gradients as previously described (Staahl et al., 2013) and analyzed by SDS-PAGE.

Figure 6. Non-redundant Functions of CHDs during Cortical Development

(A) E13.5 embryos were electroporated with the indicated vectors and immunolabeled with GFP (green) and Cux1 (red) antibodies at E18.5. Scale bar, 100 μm.

(B) Quantification of neurons expressing Cux1 and SATB2 in cortices electroporated as in (A). 5–13 embryos were analyzed per condition; n = 3. shCTL, shCHD5, and shCHD5 + hCHD5 conditions are identical to those shown in Figures 5A and 5B, because the experiments were performed at the same time, and data were split in two figures for clarity.

(C and D) Distribution of cells electroporated with the indicated vectors at E13.5 and analyzed at E18.5. 5–13 embryos were analyzed per condition; n = 3. shCTL, shCHD5, and shCHD5 + hCHD5 conditions are identical in (C) and (D), because the experiments were performed at the same time, and data were split in two graphs for clarity.

(E) E13.5 embryos were in utero electroporated with the indicated vectors and immunolabeled with GFP (green) and Cux1 (red) antibodies 5 days later. Scale bar, 100 μm.

(F) Quantification of neurons expressing Cux1 and Tbr1 in cortices in utero electroporated as in (E). 8–13 embryos were analyzed per condition; n = 3. shCTL, shCHD3, and shCHD3 + hCHD3 conditions are identical to those shown in Figures 5C and 5D, because the experiments were performed at the same time, and data were split in two figures for clarity.

(G and H) Distribution of cells electroporated with the indicated vectors at E13.5 and analyzed at E18.5. 8–13 embryos were analyzed per condition; n = 3. shCTL, shCHD3, and shCHD3 + hCHD3 conditions in (G) and (H) are identical, because the experiments were performed at the same time, and data were split in two graphs for clarity.

All data are presented as mean ± SEM. $^{**}p < 0.01$, $^{***}p < 0.001$, and $^{****}p < 0.0001$ (ns, not significant) by one-way (F) or two-way (B–D, G, and H) ANOVA with Tukey's multiple comparisons test. See also Figure S6.

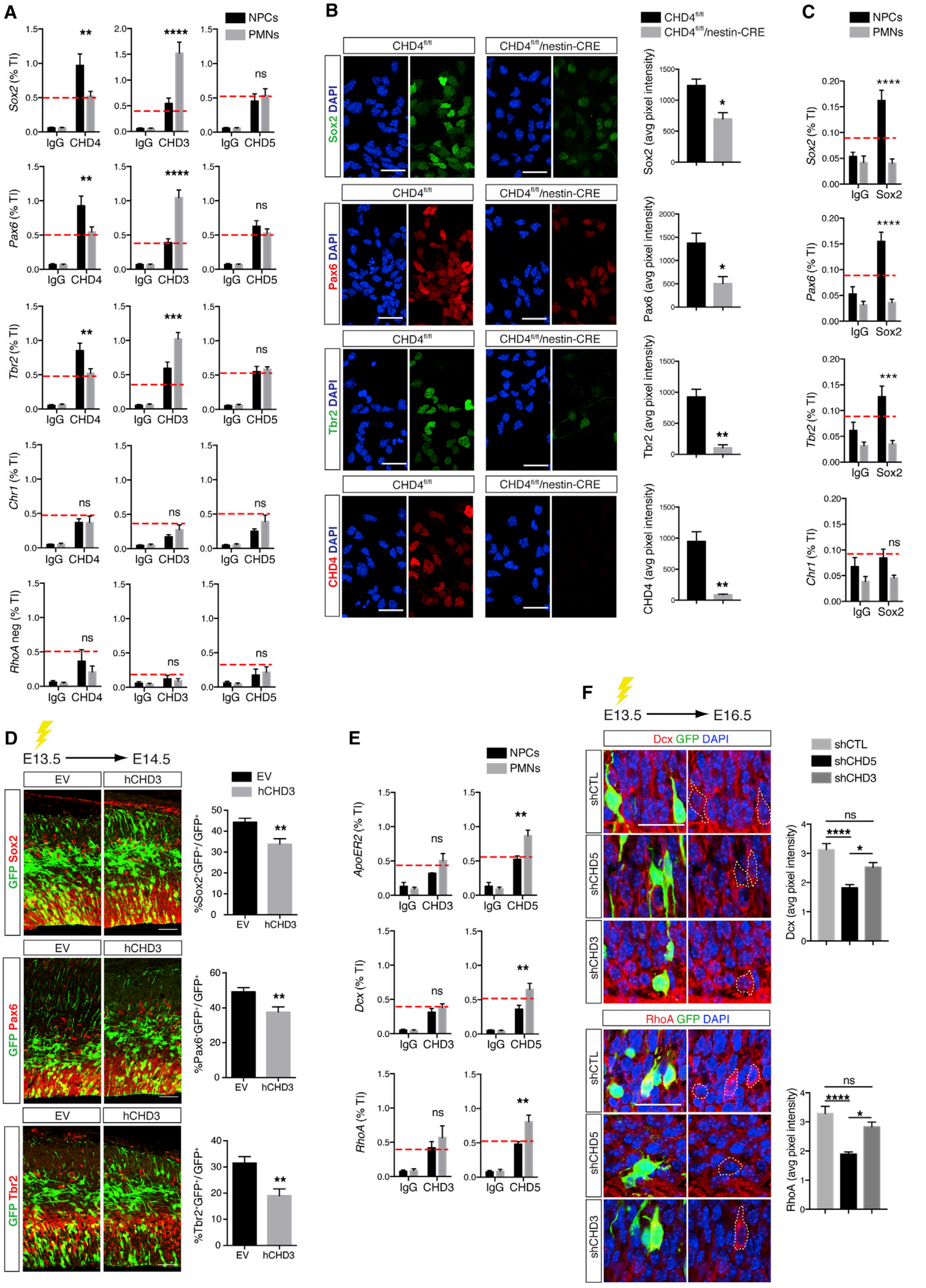

(legend on next page)

In Utero Electroporation

Timed-pregnant mice (E13.5 and E14.5) were anesthetized with isoflurane in oxygen carrier (Abbot Laboratories), and uterine horns were exposed through a small incision in the ventral peritoneum. Plasmid DNA solution (2–5 μg/μL), prepared using the EndoFree plasmid purification kit (QIAGEN), was mixed with 0.05% Fast Green (Sigma) and injected through the uterine wall into the lateral ventricles of the embryos using pulled borosilicate needles and a Femtojet microinjector (Eppendorf). Five electrical pulses were applied at 35 V (50 ms duration) across the uterine wall at 950-ms intervals using 5-mm platinum tweezertrodes (Harvard Apparatus) and an ECM-830 BTX square wave electroporator (Harvard Apparatus). The uterine horns were then replaced in the abdominal cavity, and the abdomen wall and skin were sutured. Pregnant mice were sacrificed 24 hr, 3 days, or 5 days following surgery, and embryos were subjected to immunofluorescence analyses to study neural radial migration and expression of proliferation and laminar-specific markers.

Statistical Analysis

Data are presented as mean ± SEM. Statistics for multiple comparisons was performed using either unpaired t test or one- or two-way ANOVA followed by appropriate post-test indicated in the figure legends. All analysis was performed using GraphPad Prism version 6.0 for Macintosh (GraphPad Software) (* $p < 0.05$, ** $p < 0.01$, *** $p < 0.001$, and **** $p < 0.0001$ for all statistical analysis).

ACCESSION NUMBERS

The accession number for the microarray data reported in this study is GEO: GSE70298.

SUPPLEMENTAL INFORMATION

Supplemental Information includes Supplemental Experimental Procedures, seven figures, and two tables and can be found with this article online at http://dx.doi.org/10.1016/j.celrep.2016.10.022.

AUTHOR CONTRIBUTIONS

J.N. performed most experiments and helped conceive the project and write the manuscript. J.G.S. performed experiments shown in Figures 1C, 1D, 7D, S1, S4, and S7 and helped write the manuscript. W.T.S. performed the experiments shown in Figure 7B. M.M.G.H. helped with in utero electroporation. A.N. performed the initial co-immunoprecipitation experiments. W.D.B., A.A.V., and J.A.W. performed the mass spectrometry, and R.M. was responsible for bioinformatics analysis. A.R. conceived the project and wrote the manuscript.

ACKNOWLEDGMENTS

We thank Francois Guillemot, Alison Lloyd, Emily Brookes, and Paolo Salomoni for insightful comments on the manuscript. We also thank all members of the Riccio laboratory for helpful suggestions. We are indebted to Katia Georgopoulos (Massachusetts General Hospital) for providing the CHD4$^{fl/fl}$ transgenic mice, Mike Pazin and Taro Tachibana for providing the CHD5 antibodies, and Francois Guillemot and Odd Stokke Gabrielsen for providing the DNA vectors.

This work was supported by Wellcome Trust Investigator Award 103717/Z/14/Z (to A.R.), MRC Senior Non Clinical Fellowship SNCF G0802010 (to A.R.), and Ruth L. Kirschstein National Research Service Award GM007185 (to W.D.B.).

Received: January 7, 2016
Revised: August 3, 2016
Accepted: October 7, 2016
Published: November 1, 2016

REFERENCES

Alcamo, E.A., Chirivella, L., Dautzenberg, M., Dobreva, G., Fariñas, I., Grosschedl, R., and McConnell, S.K. (2008). Satb2 regulates callosal projection neuron identity in the developing cerebral cortex. Neuron *57*, 364–377.

Allen, A.S., Berkovic, S.F., Cossette, P., Delanty, N., Dlugos, D., Eichler, E.E., Epstein, M.P., Glauser, T., Goldstein, D.B., Han, Y., et al.; Epi4K Consortium; Epilepsy Phenome/Genome Project (2013). De novo mutations in epileptic encephalopathies. Nature *501*, 217–221.

Amaya, M., Desai, M., Gnanapragasam, M.N., Wang, S.Z., Zu Zhu, S., Williams, D.C., Jr., and Ginder, G.D. (2013). Mi2β-mediated silencing of the fetal γ-globin gene in adult erythroid cells. Blood *121*, 3493–3501.

Arlotta, P., Molyneaux, B.J., Chen, J., Inoue, J., Kominami, R., and Macklis, J.D. (2005). Neuronal subtype-specific genes that control corticospinal motor neuron development in vivo. Neuron *45*, 207–221.

Arnold, S.J., Huang, G.-J., Cheung, A.F.P., Era, T., Nishikawa, S., Bikoff, E.K., Molnár, Z., Robertson, E.J., and Groszer, M. (2008). The T-box transcription factor Eomes/Tbr2 regulates neurogenesis in the cortical subventricular zone. Genes Dev. *22*, 2479–2484.

Barnes, A.P., and Polleux, F. (2009). Establishment of axon-dendrite polarity in developing neurons. Annu. Rev. Neurosci. *32*, 347–381.

Basta, J., and Rauchman, M. (2015). The nucleosome remodeling and deacetylase complex in development and disease. Transl. Res. *165*, 36–47.

Borrelli, E., Nestler, E.J., Allis, C.D., and Sassone-Corsi, P. (2008). Decoding the epigenetic language of neuronal plasticity. Neuron *60*, 961–974.

Bowen, N.J., Fujita, N., Kajita, M., and Wade, P.A. (2004). Mi-2/NuRD: multiple complexes for many purposes. Biochim. Biophys. Acta Structure and Expression *1677*, 52–57.

Britanova, O., de Juan Romero, C., Cheung, A., Kwan, K.Y., Schwark, M., Gyorgy, A., Vogel, T., Akopov, S., Mitkovski, M., Agoston, D., et al. (2008). Satb2 is a postmitotic determinant for upper-layer neuron specification in the neocortex. Neuron *57*, 378–392.

Cappello, S. (2013). Small Rho-GTPases and cortical malformations: fine-tuning the cytoskeleton stability. Small GTPases *4*, 51–56.

Dominguez, M.H., Ayoub, A.E., and Rakic, P. (2012). POU-III transcription factors (Brn1, Brn2, and Oct6) influence neurogenesis, molecular identity, and migratory destination of upper-layer cells of the cerebral cortex. Cereb. Cortex *23*, 2632–2643.

Figure 7. Specific CHD Subunits Regulate the Expression of Genes Necessary for Cortical Development

(A) ChIP of CHDs on *Sox2*, *Pax6*, and *Tbr2* promoters in NPCs and PMNs. Chromosome 1 (*Chr1*) and *RhoA* intergenic region (*RhoA neg*) were used as negative controls. The red dotted line indicates background signal detected on *Chr1* (CHD3 ChIP n = 8, CHD4 ChIP n = 7, and CHD5 ChIP n = 7).
(B) Immunofluorescence of Sox2, Pax6, and Tbr2 in NPCs derived from CHD4$^{fl/fl}$/nestin-CRE and control E12.5 embryos; n = 3. Scale bar, 50 μm.
(C) ChIP of Sox2 binding to *Pax6*, *Sox2*, and *Tbr2* promoters in NPCs and PMNs. *Chr1* was used as negative control; n = 4.
(D) E13.5 embryos were in utero electroporated with either EV or hCHD3 expressing vectors and immunolabeled for GFP (green) and Sox2, Pax6, or Tbr2 (red) at E14.5. Five to nine embryos were analyzed per condition; n = 3. Scale bar, 50 μm.
(E) ChIP of CHD subunits binding on *ApoER2*, *Dcx*, and *RhoA* promoters in NPCs and PMNs (CHD3 ChIP n = 6 and CHD5 ChIP n = 5).
(F) Expression of Dcx and RhoA in the cortex of mice electroporated with shCTL, shCHD5, or shCHD3 at E13.5 and harvested at E16.5 25–50 cells were analyzed per embryo; n = 3. Average pixel intensity of Dcx and RhoA in GFP-expressing cells was normalized to background (ImageJ). Scale bar, 25 μm.
Data are presented as mean ± SEM. *$p < 0.05$, **$p < 0.01$, ***$p < 0.001$, and ****$p < 0.0001$ (ns, not significant) by two-way ANOVA (A, C, and E) with Sidak's multiple comparisons test, unpaired t test (B and D), or one-way ANOVA (F) with Tukey's multiple comparisons test. See also Figure S7.

Dubois, N.C., Hofmann, D., Kaloulis, K., Bishop, J.M., and Trumpp, A. (2006). Nestin-Cre transgenic mouse line Nes-Cre1 mediates highly efficient Cre/loxP mediated recombination in the nervous system, kidney, and somite-derived tissues. Genesis *44*, 355–360.

Egan, C.M., Nyman, U., Skotte, J., Streubel, G., Turner, S., O'Connell, D.J., Rraklli, V., Dolan, M.J., Chadderton, N., Hansen, K., et al. (2013). CHD5 is required for neurogenesis and has a dual role in facilitating gene expression and polycomb gene repression. Dev. Cell *26*, 223–236.

Elias, J.E., and Gygi, S.P. (2007). Target-decoy search strategy for increased confidence in large-scale protein identifications by mass spectrometry. Nat. Methods *4*, 207–214.

Engelen, E., Akinci, U., Bryne, J.C., Hou, J., Gontan, C., Moen, M., Szumska, D., Kockx, C., van Ijcken, W., Dekkers, D.H.W., et al. (2011). Sox2 cooperates with Chd7 to regulate genes that are mutated in human syndromes. Nat. Genet. *43*, 607–611.

Englund, C., Fink, A., Lau, C., Pham, D., Daza, R.A., Bulfone, A., Kowalczyk, T., and Hevner, R.F. (2005). Pax6, Tbr2, and Tbr1 are expressed sequentially by radial glia, intermediate progenitor cells, and postmitotic neurons in developing neocortex. J. Neurosci. *25*, 247–251.

Florens, L., Carozza, M.J., Swanson, S.K., Fournier, M., Coleman, M.K., Workman, J.L., and Washburn, M.P. (2006). Analyzing chromatin remodeling complexes using shotgun proteomics and normalized spectral abundance factors. Methods *40*, 303–311.

Florio, M., and Huttner, W.B. (2014). Neural progenitors, neurogenesis and the evolution of the neocortex. Development *141*, 2182–2194.

Francis, F., Koulakoff, A., Boucher, D., Chafey, P., Schaar, B., Vinet, M.-C., Friocourt, G., McDonnell, N., Reiner, O., Kahn, A., et al. (1999). Doublecortin is a developmentally regulated, microtubule-associated protein expressed in migrating and differentiating neurons. Neuron *23*, 247–256.

Gleeson, J.G., Lin, P.T., Flanagan, L.A., and Walsh, C.A. (1999). Doublecortin is a microtubule-associated protein and is expressed widely by migrating neurons. Neuron *23*, 257–271.

Götz, M., Stoykova, A., and Gruss, P. (1998). Pax6 controls radial glia differentiation in the cerebral cortex. Neuron *21*, 1031–1044.

Graham, V., Khudyakov, J., Ellis, P., and Pevny, L. (2003). SOX2 functions to maintain neural progenitor identity. Neuron *39*, 749–765.

Haberland, M., Montgomery, R.L., and Olson, E.N. (2009). The many roles of histone deacetylases in development and physiology: implications for disease and therapy. Nat. Rev. Genet. *10*, 32–42.

Hagelkruys, A., Lagger, S., Krahmer, J., Leopoldi, A., Artaker, M., Pusch, O., Zezula, J., Weissmann, S., Xie, Y., Schöfer, C., et al. (2014). A single allele of Hdac2 but not Hdac1 is sufficient for normal mouse brain development in the absence of its paralog. Development *141*, 604–616.

Hagelkruys, A., Mattes, K., Moos, V., Rennmayr, M., Ringbauer, M., Sawicka, A., and Seiser, C. (2015). Essential nonredundant function of the catalytic activity of histone deacetylase 2 in mouse development. Mol. Cell. Biol. *36*, 462–474.

Hevner, R.F., Shi, L., Justice, N., Hsueh, Y., Sheng, M., Smiga, S., Bulfone, A., Goffinet, A.M., Campagnoni, A.T., and Rubenstein, J.L. (2001). Tbr1 regulates differentiation of the preplate and layer 6. Neuron *29*, 353–366.

Hirabayashi, Y., and Gotoh, Y. (2010). Epigenetic control of neural precursor cell fate during development. Nat. Rev. Neurosci. *11*, 377–388.

Kashiwagi, M., Morgan, B.A., and Georgopoulos, K. (2007). The chromatin remodeler Mi-2beta is required for establishment of the basal epidermis and normal differentiation of its progeny. Development *134*, 1571–1582.

Knock, E., Pereira, J., Lombard, P.D., Dimond, A., Leaford, D., Livesey, F.J., and Hendrich, B. (2015). The methyl binding domain 3/nucleosome remodelling and deacetylase complex regulates neural cell fate determination and terminal differentiation in the cerebral cortex. Neural Dev. *10*, 13.

Kwan, K.Y., Sestan, N., and Anton, E.S. (2012). Transcriptional co-regulation of neuronal migration and laminar identity in the neocortex. Development *139*, 1535–1546.

Lai, A.Y., and Wade, P.A. (2011). Cancer biology and NuRD: a multifaceted chromatin remodelling complex. Nat. Rev. Cancer *11*, 588–596.

Lai, T., Jabaudon, D., Molyneaux, B.J., Azim, E., Arlotta, P., Menezes, J.R.L., and Macklis, J.D. (2008). SOX5 controls the sequential generation of distinct corticofugal neuron subtypes. Neuron *57*, 232–247.

Martínez-Cerdeño, V., Noctor, S.C., and Kriegstein, A.R. (2006). The role of intermediate progenitor cells in the evolutionary expansion of the cerebral cortex. Cereb. Cortex *16* (*Suppl 1*), i152–i161.

Micucci, J.A., Sperry, E.D., and Martin, D.M. (2015). Chromodomain helicase DNA-binding proteins in stem cells and human developmental diseases. Stem Cells Dev. *24*, 917–926.

Montgomery, R.L., Hsieh, J., Barbosa, A.C., Richardson, J.A., and Olson, E.N. (2009). Histone deacetylases 1 and 2 control the progression of neural precursors to neurons during brain development. Proc. Natl. Acad. Sci. USA *106*, 7876–7881.

Narlikar, G.J., Sundaramoorthy, R., and Owen-Hughes, T. (2013). Mechanisms and functions of ATP-dependent chromatin-remodeling enzymes. Cell *154*, 490–503.

Neale, B.M., Kou, Y., Liu, L., Ma'ayan, A., Samocha, K.E., Sabo, A., Lin, C.F., Stevens, C., Wang, L.S., Makarov, V., et al. (2012). Patterns and rates of exonic de novo mutations in autism spectrum disorders. Nature *485*, 242–245.

Nieto, M., Monuki, E.S., Tang, H., Imitola, J., Haubst, N., Khoury, S.J., Cunningham, J., Gotz, M., and Walsh, C.A. (2004). Expression of Cux-1 and Cux-2 in the subventricular zone and upper layers II-IV of the cerebral cortex. J. Comp. Neurol. *479*, 168–180.

Noctor, S.C., Martínez-Cerdeño, V., Ivic, L., and Kriegstein, A.R. (2004). Cortical neurons arise in symmetric and asymmetric division zones and migrate through specific phases. Nat. Neurosci. *7*, 136–144.

Nott, A., Watson, P.M., Robinson, J.D., Crepaldi, L., and Riccio, A. (2008). S-Nitrosylation of histone deacetylase 2 induces chromatin remodelling in neurons. Nature *455*, 411–415.

Nott, A., Nitarska, J., Veenvliet, J.V., Schacke, S., Derijck, A.A., Sirko, P., Muchardt, C., Pasterkamp, R.J., Smidt, M.P., and Riccio, A. (2013). S-nitrosylation of HDAC2 regulates the expression of the chromatin-remodeling factor Brm during radial neuron migration. Proc. Natl. Acad. Sci. USA *110*, 3113–3118.

O'Roak, B.J., Vives, L., Fu, W., Egertson, J.D., Stanaway, I.B., Phelps, I.G., Carvill, G., Kumar, A., Lee, C., Ankenman, K., et al. (2012). Multiplex targeted sequencing identifies recurrently mutated genes in autism spectrum disorders. Science *338*, 1619–1622.

Potts, R.C., Zhang, P., Wurster, A.L., Precht, P., Mughal, M.R., Wood, W.H., 3rd, Zhang, Y., Becker, K.G., Mattson, M.P., and Pazin, M.J. (2011). CHD5, a brain-specific paralog of Mi2 chromatin remodeling enzymes, regulates expression of neuronal genes. PLoS ONE *6*, e24515.

Quinn, J.C., Molinek, M., Martynoga, B.S., Zaki, P.A., Faedo, A., Bulfone, A., Hevner, R.F., West, J.D., and Price, D.J. (2007). Pax6 controls cerebral cortical cell number by regulating exit from the cell cycle and specifies cortical cell identity by a cell autonomous mechanism. Dev. Biol. *302*, 50–65.

Reynolds, N., Latos, P., Hynes-Allen, A., Loos, R., Leaford, D., O'Shaughnessy, A., Mosaku, O., Signolet, J., Brennecke, P., Kalkan, T., et al. (2012a). NuRD suppresses pluripotency gene expression to promote transcriptional heterogeneity and lineage commitment. Cell Stem Cell *10*, 583–594.

Reynolds, N., Salmon-Divon, M., Dvinge, H., Hynes-Allen, A., Balasooriya, G., Leaford, D., Behrens, A., Bertone, P., and Hendrich, B. (2012b). NuRD-mediated deacetylation of H3K27 facilitates recruitment of Polycomb Repressive Complex 2 to direct gene repression. EMBO J. *31*, 593–605.

Riccio, A. (2010). Dynamic epigenetic regulation in neurons: enzymes, stimuli and signaling pathways. Nat. Neurosci. *13*, 1330–1337.

Sessa, A., Mao, C.A., Hadjantonakis, A.K., Klein, W.H., and Broccoli, V. (2008). Tbr2 directs conversion of radial glia into basal precursors and guides neuronal

amplification by indirect neurogenesis in the developing neocortex. Neuron *60*, 56–69.

Shimbo, T., Du, Y., Grimm, S.A., Dhasarathy, A., Mav, D., Shah, R.R., Shi, H., and Wade, P.A. (2013). MBD3 localizes at promoters, gene bodies and enhancers of active genes. PLoS Genet. *9*, e1004028.

Staahl, B.T., Tang, J., Wu, W., Sun, A., Gitler, A.D., Yoo, A.S., and Crabtree, G.R. (2013). Kinetic analysis of npBAF to nBAF switching reveals exchange of SS18 with CREST and integration with neural developmental pathways. J. Neurosci. *33*, 10348–10361.

Tong, J.K., Hassig, C.A., Schnitzler, G.R., Kingston, R.E., and Schreiber, S.L. (1998). Chromatin deacetylation by an ATP-dependent nucleosome remodelling complex. Nature *395*, 917–921.

Topark-Ngarm, A., Golonzhka, O., Peterson, V.J., Barrett, B., Jr., Martinez, B., Crofoot, K., Filtz, T.M., and Leid, M. (2006). CTIP2 associates with the NuRD complex on the promoter of p57KIP2, a newly identified CTIP2 target gene. J. Biol. Chem. *281*, 32272–32283.

Trommsdorff, M., Gotthardt, M., Hiesberger, T., Shelton, J., Stockinger, W., Nimpf, J., Hammer, R.E., Richardson, J.A., and Herz, J. (1999). Reeler/Disabled-like disruption of neuronal migration in knockout mice lacking the VLDL receptor and ApoE receptor 2. Cell *97*, 689–701.

Williams, C.J., Naito, T., Arco, P.G., Seavitt, J.R., Cashman, S.M., De Souza, B., Qi, X., Keables, P., Von Andrian, U.H., and Georgopoulos, K. (2004). The chromatin remodeler Mi-2beta is required for CD4 expression and T cell development. Immunity *20*, 719–733.

Wohlschlegel, J. (2009). Identification of SUMO-conjugated proteins and their SUMO attachment sites using proteomic mass spectrometry. In SUMO Protocols, H. Ulrich, ed. (Humana Press), pp. 33–49.

Xue, Y., Wong, J., Moreno, G.T., Young, M.K., Côté, J., and Wang, W. (1998). NURD, a novel complex with both ATP-dependent chromatin-remodeling and histone deacetylase activities. Mol. Cell *2*, 851–861.

Yamada, T., Yang, Y., Hemberg, M., Yoshida, T., Cho, H.Y., Murphy, J.P., Fioravante, D., Regehr, W.G., Gygi, S.P., Georgopoulos, K., and Bonni, A. (2014). Promoter decommissioning by the NuRD chromatin remodeling complex triggers synaptic connectivity in the mammalian brain. Neuron *83*, 122–134.

Yang, Y., Yamada, T., Hill, K.K., Hemberg, M., Reddy, N.C., Cho, H.Y., Guthrie, A.N., Oldenborg, A., Heiney, S.A., Ohmae, S., et al. (2016). Chromatin remodeling inactivates activity genes and regulates neural coding. Science *353*, 300–305.

Yoo, A.S., and Crabtree, G.R. (2009). ATP-dependent chromatin remodeling in neural development. Curr. Opin. Neurobiol. *19*, 120–126.

Zhang, Y., LeRoy, G., Seelig, H.-P., Lane, W.S., and Reinberg, D. (1998). The dermatomyositis-specific autoantigen Mi2 is a component of a complex containing histone deacetylase and nucleosome remodeling activities. Cell *95*, 279–289.

Zhang, J., Jackson, A.F., Naito, T., Dose, M., Seavitt, J., Liu, F., Heller, E.J., Kashiwagi, M., Yoshida, T., Gounari, F., et al. (2011). Harnessing of the nucleosome-remodeling-deacetylase complex controls lymphocyte development and prevents leukemogenesis. Nat. Immunol. *13*, 86–94.

Cell Reports, Volume *17*

Supplemental Information

A Functional Switch of NuRD Chromatin Remodeling Complex Subunits Regulates Mouse Cortical Development

Justyna Nitarska, Jacob G. Smith, William T. Sherlock, Michele M.G. Hillege, Alexi Nott, William D. Barshop, Ajay A. Vashisht, James A. Wohlschlegel, Richard Mitter, and Antonella Riccio

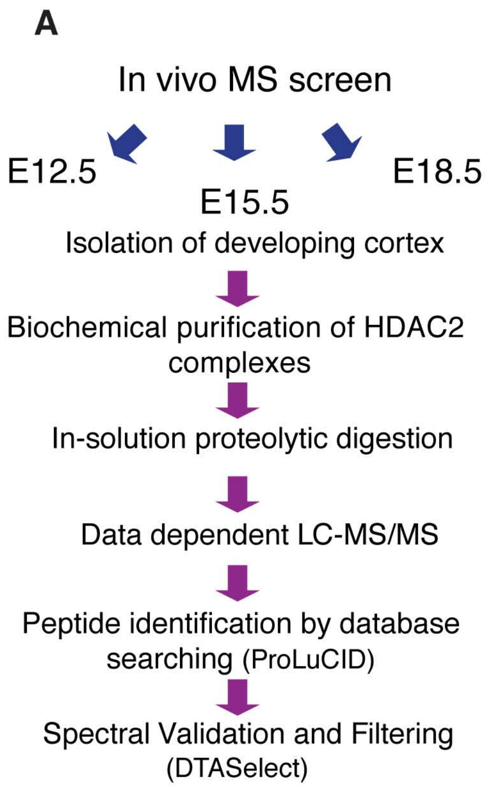

B

	Name	Locus ID	MW	E12.5	E15.5	E18.5
NuRD	HDAC2	spIP70288IHDAC2_MOUSE	55302	975.513	595.091	634.144
	HDAC1	spIO09106IHDAC1_MOUSE	55075	631.452	248.650	351.115
	CHD3	trIB1AR17IB1AR17_MOUSE	232744	337.988	439.650	437.652
	CHD4	trIE9QAS4IE9QAS4_MOUSE	216369	984.385	493.971	678.162
	CHD5	trIE9PYL1IE9PYL1_MOUSE	222683	201.085	256.550	181.902
	MTA1	trIF8WHY8IF8WHY8_MOUSE	79189	1319.318	805.688	907.494
	MTA2	spIQ9R190IMTA2_MOUSE	75030	1203.327	848.772	1056.828
	MTA3	trIE9Q794IE9Q794_MOUSE	58454	926.168	573.957	620.881
	MBD2	spIQ9Z2E1IMBD2_MOUSE	43501	131.953	111.343	81.757
	MBD3	trID3YTR5ID3YTR5_MOUSE	29070	1209.492	782.440	838.603
	GATA2a	trIE9QMN5IE9QMN5_MOUSE	67462	1078.565	446.680	591.456
	GATA2b	spIQ8VHR5IP66B_MOUSE	65411	998.504	783.786	830.312
	Rbbp4	spIQ60972IRBBP4_MOUSE	47656	3415.442	2917.601	3606.601
	Rbbp7	spIQ60973IRBBP7_MOUSE	47790	2038.247	1041.226	1228.747
CoREST	CoREST1	trIK3W4P9IK3W4P9_MOUSE	52715	471.498	230.480	332.430
	CoREST2	spIQ8C796IRCOR2_MOUSE	57908	626.716	273.226	536.234
	CoREST3	spIQ6PGA0IRCOR3_MOUSE	49779	138.432	81.767	0
mSin3	Sin3a	spIQ60520ISIN3A_MOUSE	145088	336.912	296.693	288.451
	Sin3b	spIQ62141ISIN3B_MOUSE	126405	106.613	205.710	162.940
	Sds3	spIQ8BR65ISDS3_MOUSE	38107	214.137	196.751	221.129
	Sap130	trIJ3QNK5IJ3QNK5_MOUSE	111240	66.449	82.859	82.343
	Sap30L	spIQ5SQF8ISP30L_MOUSE	20745	0	50.655	53.136

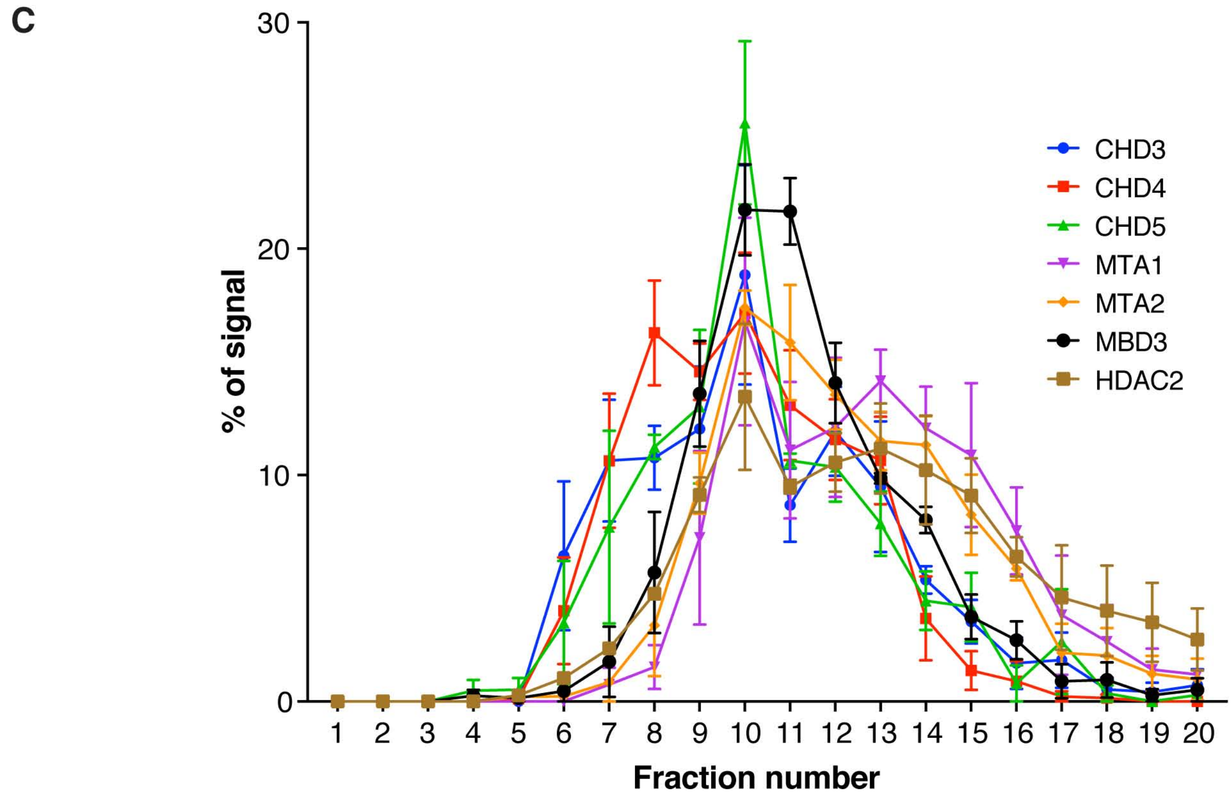

Figure S1. Related to Fig.1 NuRD complex subunits co-immunoprecipitate with HDAC2 in the developing cortex (**A**) Workflow of purification and identification of proteins that co-immunoprecipitate with HDAC2 in the embryonic cortex at the indicated stages. (**B**) List of the most abundant proteins that interact with HDAC2 identified by mass spectrometry. The numbers shown represent the normalised spectral abundance factor (NSAF) for each protein. MW, molecular weight. Complete list of HDAC2 interacting proteins can be found in **Table S1**. (**C**) Densitometry analysis of the glycerol gradient analysis represented in **Fig.1D**. For each protein, the percentage of signal detected in each fraction was assessed using Image J. n=3, except for fraction 11 (n=2).

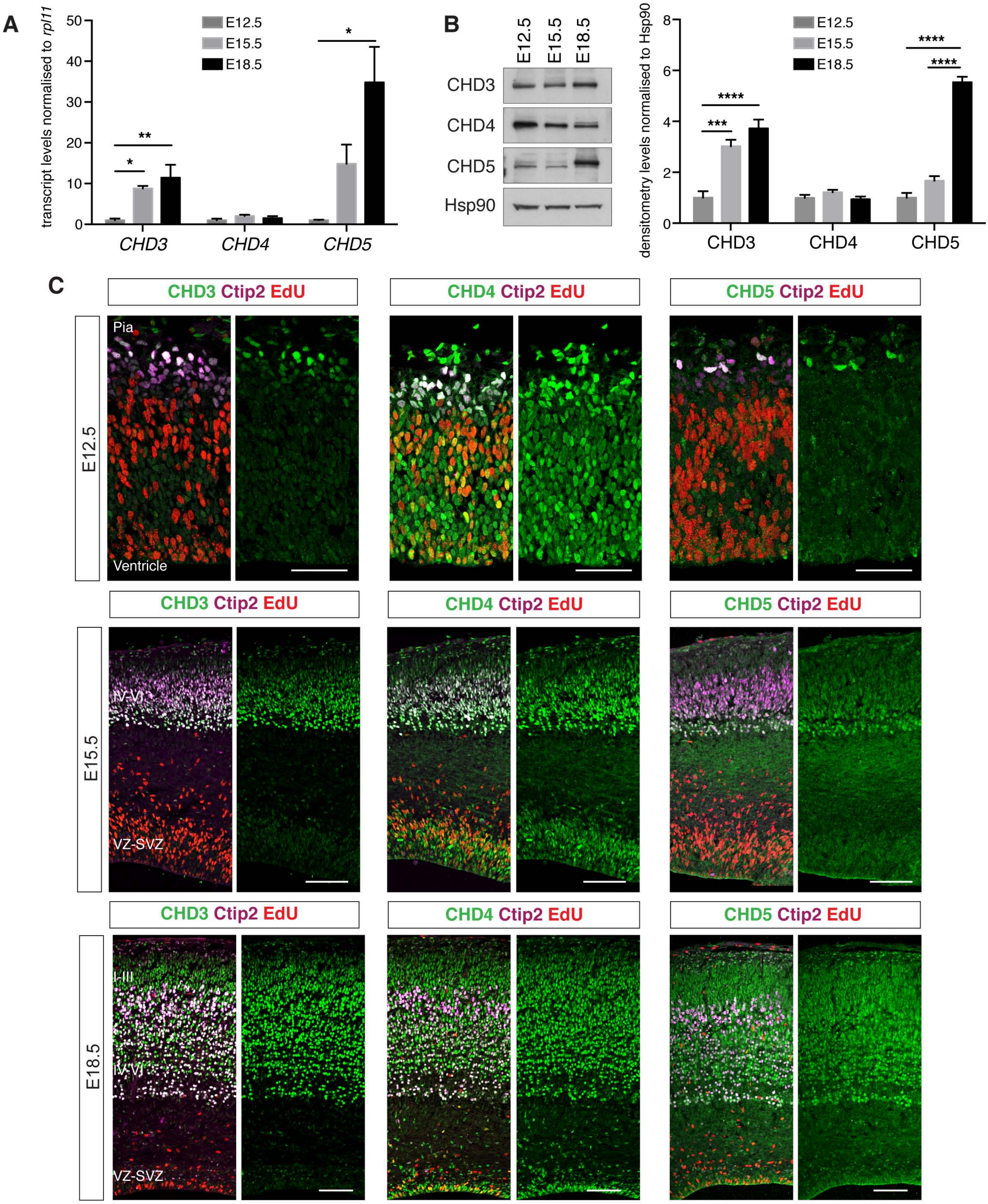

Figure S2. Related to Fig.1 CHD3, CHD4 and CHD5 proteins are differentially expressed during cortical development *in vivo*
(**A**) RT-qPCR analysis of *CHD3*, *CHD4* and *CHD5* mRNA at the indicated embryonic stages. Transcript levels were normalised to *rpl11* transcript. n=6. Data are represented as mean ± SEM. * $p<0.05$, ** $p<0.01$, ns, not significant; one way Anova with Tukey's multiple comparisons test. (**B**) Western blot and densitometry analysis of CHD3, CHD4 and CHD5 in embryonic cortex at the indicated embryonic stages. Hsp90 was used as a loading control. Data are represented as mean ± SEM of 7 independent experiments. *** $p<0.001$, **** $p<0.0001$; one way Anova with Tukey's multiple comparisons test. (**C**) Immunofluorescence analysis of CHD3, CHD4 and CHD5 (green) in cortical coronal sections dissected 2h after the administration of an EdU pulse at the indicated developmental stages. Proliferating neural progenitors are labelled with EdU (red) and deep layer neurons are labelled with Ctip2 (magenta) antibody. Bar=50 µm (E12.5) and 100 µm (E15.5 and E18.5) n=3.

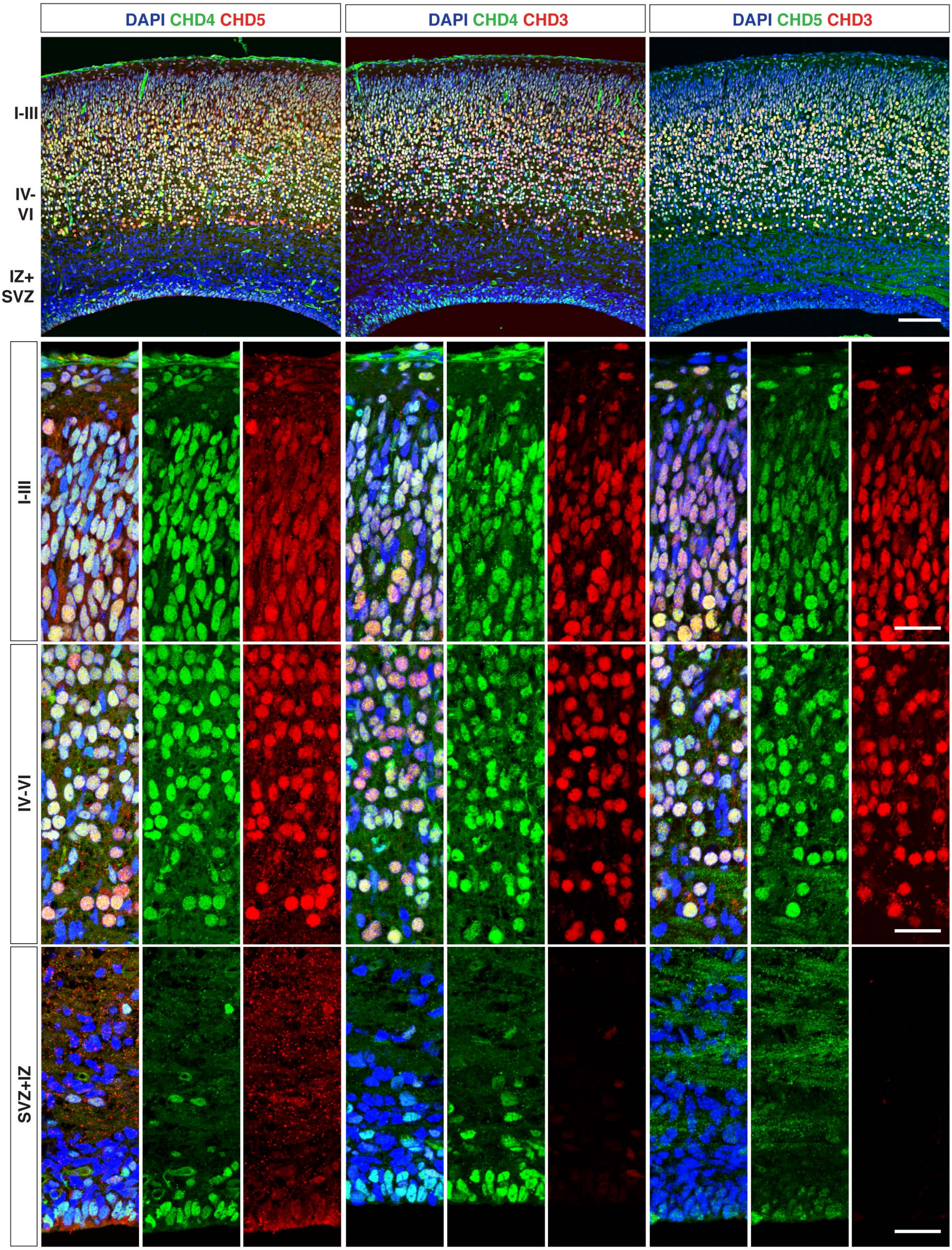

Figure S3. Related to Fig.1 CHD3, CHD4 and CHD5 proteins are co-expressed in the majority of cells within the CP. Coronal sections of E18.5 cortex immunostained with CHD3, CHD4 and CHD5. Bar=100µm. High magnification images demonstrate co-localisation of CHD4 and CHD5, CHD4 and CHD3 and CHD5 and CHD3 proteins in cells within the upper (I-III) and deeper (IV-VI) layers of the CP, as well as the SVZ. Bar= 25µm

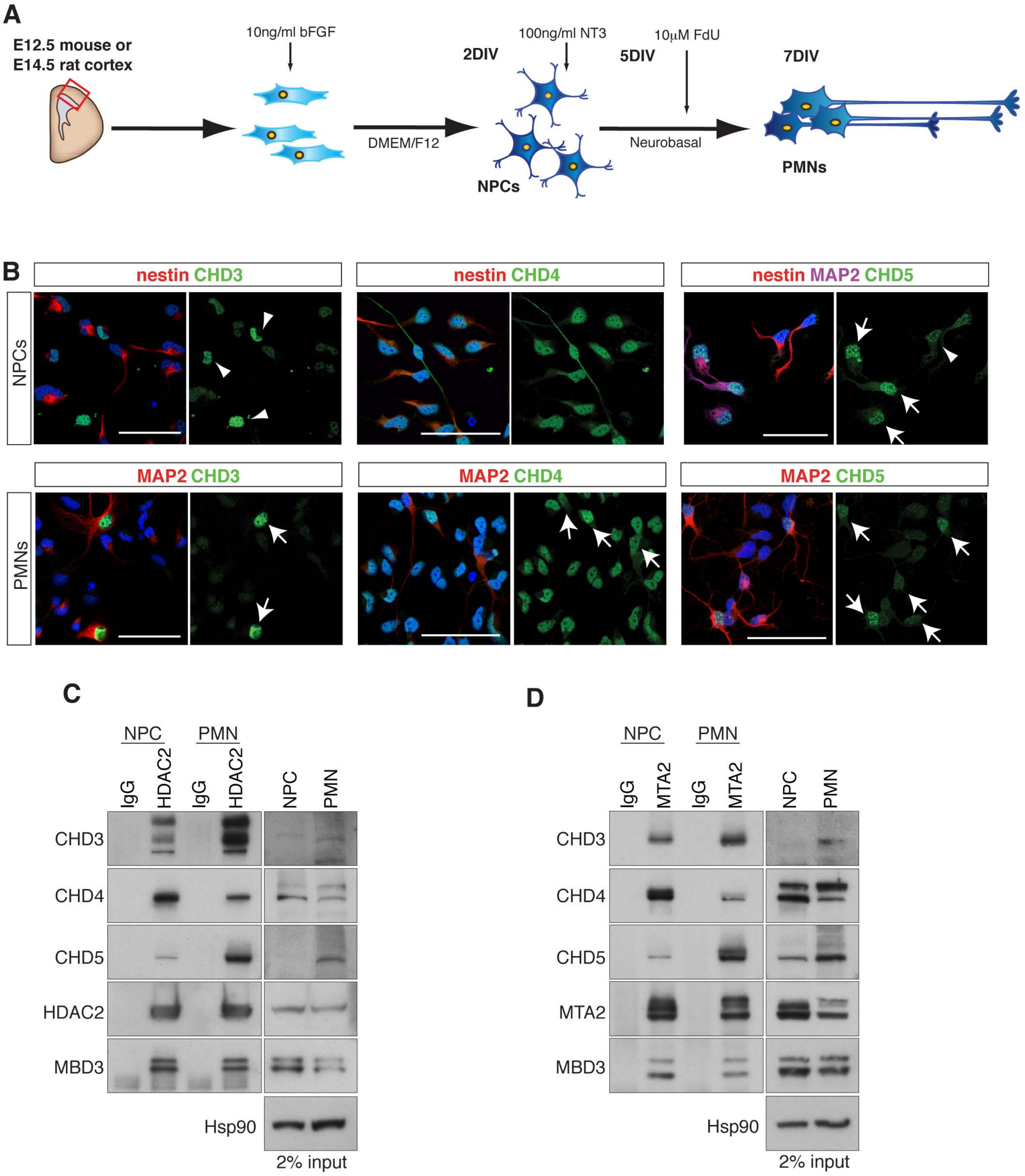

Figure S4. Related to Fig.1 CHD3, CHD4 and CHD5 proteins are differentially expressed during neuronal differentiation *in vitro*
(**A**) Diagram of cortical NPC cultures derived from E12.5 mouse or E14.5 rat embryos and differentiated into PMNs. (**B**) CHD3, CHD4 and CHD5 immunostaining (green) of rat cortical NPCs and PMNs. Neural progenitors were immunostained with nestin (arrowheads) and postmitotic neurons with MAP2 (arrows). n=3. Bar= 50µm (**C**) Western blot of proteins co-immunoprecipitating with HDAC2 in rat cortical NPCs and PMNs. Hsp90 was used as a loading control. Representative blot; n=3. (**D**) Western blot of proteins co-immunoprecipitating with MTA2 in rat cortical NPCs and PMNs. Hsp90 was used as a loading control. Representative blot; n=3

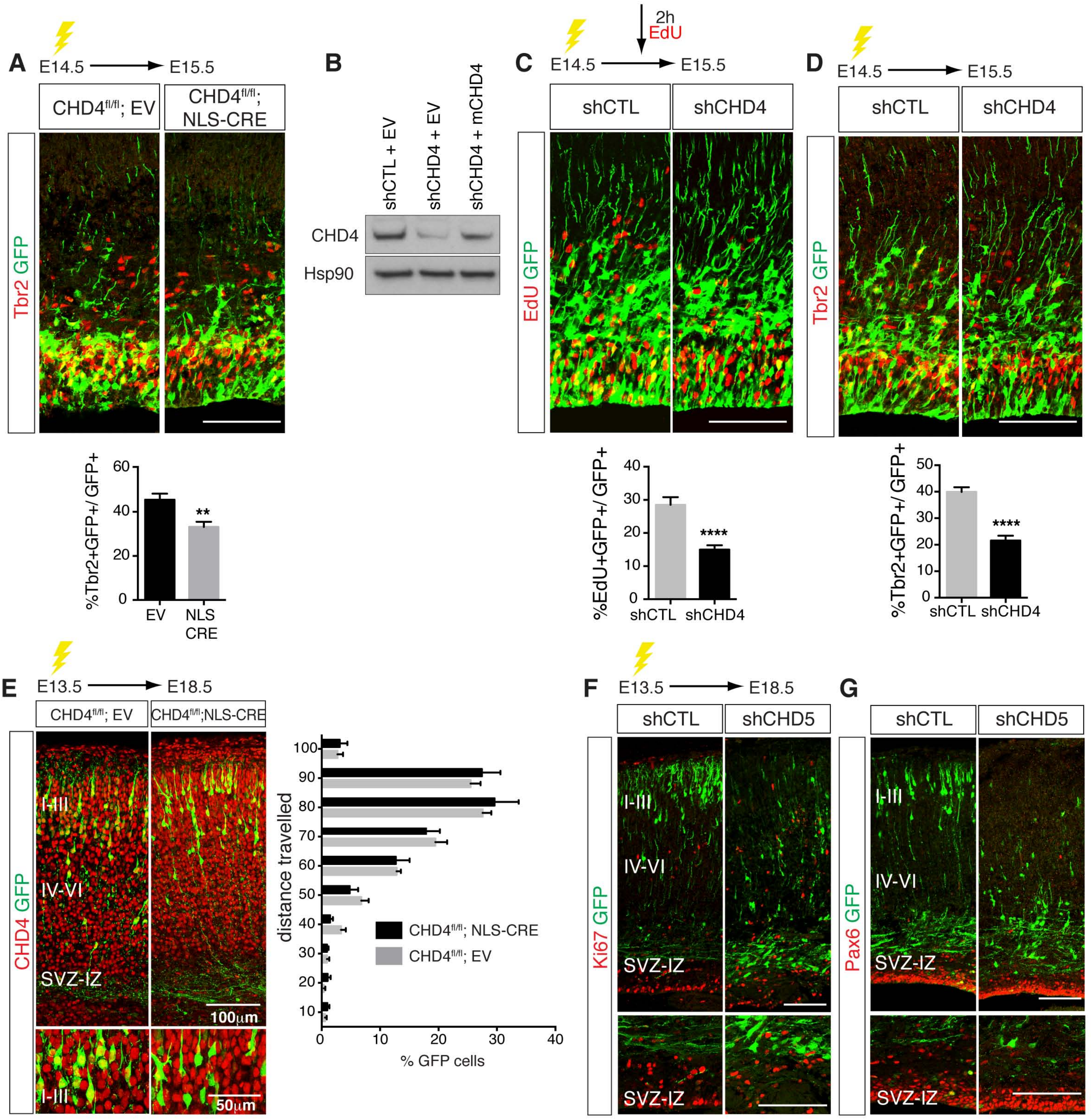

Figure S5. Related to Fig.3 and Fig.5 Proliferation analysis of cells depleted of CHD4 or CHD5 (**A**) E14.5 CHD4$^{fl/fl}$ embryos were subjected to *in utero* electroporation with GFP empty vector (EV) or NLS CRE constructs and immunolabeled with GFP (green) and Tbr2 (red) antibodies at E15.5. Shown are representative images and quantification of Tbr2 positive cells. Data were obtained from 6 to 10 embryos per condition harvested from 3 independent experiments. Bar=100μm. (**B**) shRNA designed against the 3' UTR region of CHD4 was expressed in N2A cells. Cells were cotransfected with either EV or a vector expressing a shRNA-resistant CHD4 coding sequence. CHD4 levels were assessed by western blot with Hsp90 as a loading control. Representative blot of n=3. (**C**) E14.5 embryos were *in utero* electroporated with shCTL or shCHD4 constructs. Embryos were subjected to a 2h EdU pulse and harvested at E15.5 then immunolabeled with GFP (green) antibody and EdU (red). Shown are representative images and quantification of EdU positive cells. Data were obtained from 6 to 14 embryos per condition, harvested from three independent experiments. Bar=100μm. (**D**) E14.5 embryos were *in utero* electroporated with either shCTL or shCHD4 constructs and immunolabeled with GFP (green) and Tbr2 (red) antibodies at E15.5. Shown are representative images and quantification of Tbr2 positive cells. Data were obtained from 10 to 14 embryos per condition harvested from 3 independent experiments. Bar=100μm. (**E**) E13.5 CHD4$^{fl/fl}$ embryos were *in utero* electroporated with either EV or NLS CRE constructs and analysed at E18.5. Shown are representative images of coronal sections immunostained for CHD4 (red) and GFP (green) and quantification of the migration of electroporated neurons at E18.5. Data were obtained from 8 embryos per condition harvested from 3 independent experiments. Bar=100μm (top) and 50μm (bottom). (**F and G**) E13.5 embryos were subjected to *in utero* electroporation with indicated shRNA-GFP constructs and analysed at E18.5. Shown are representative images of coronal sections stained for GFP (green) and Ki67 (red) (**F**) or Pax6 (red) (**G**). Bar= 100μm; n= 5. Data are represented as mean ± SEM. ** p<0.01, **** p<0.0001, unpaired t test.

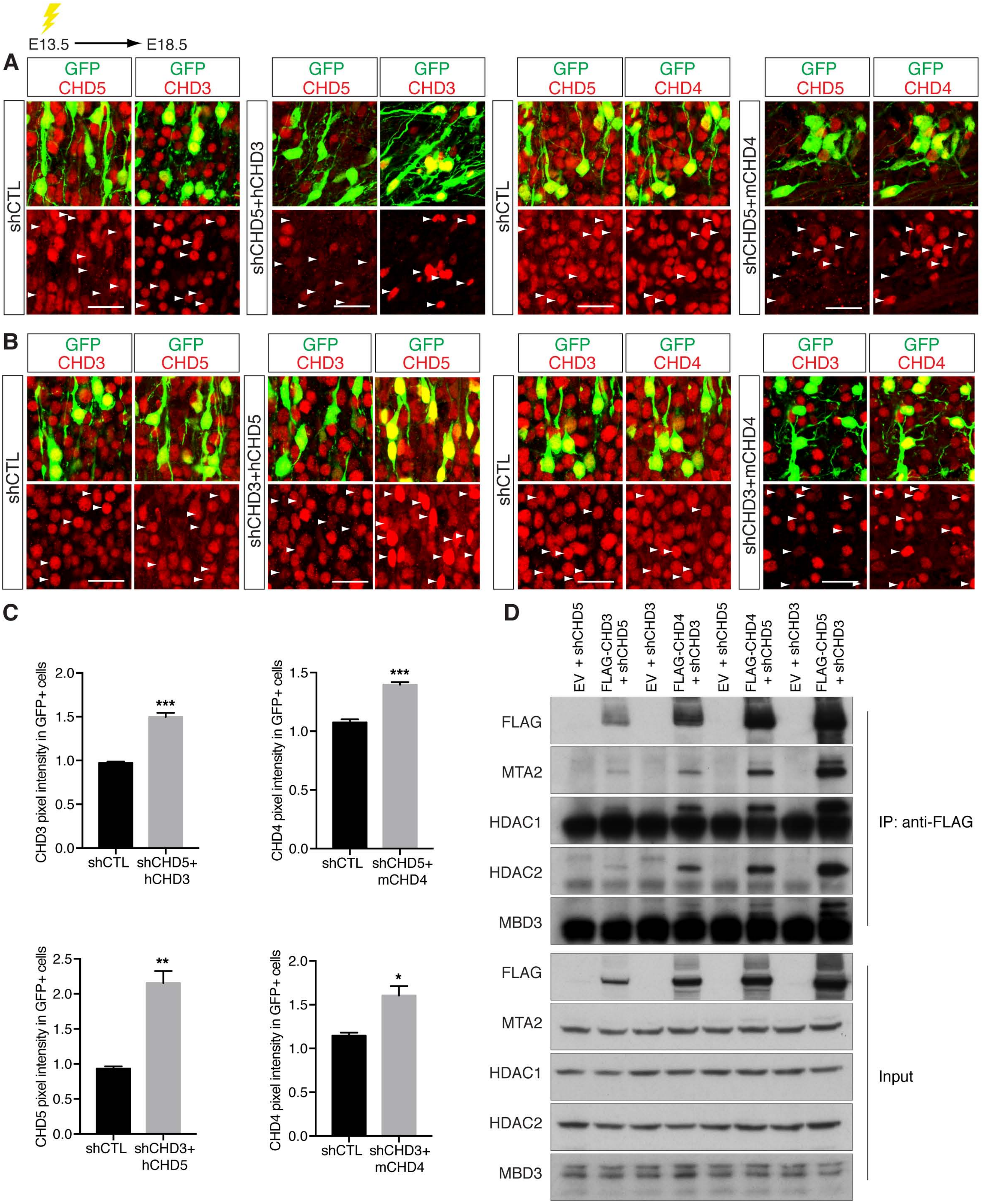

Figure S6. Related to Fig.6 Overexpression of CHD rescue constructs leads to the formation of NuRD complexes
(**A**) Immunofluorescence analysis of E13.5 embryos *in utero* electroporated with either shCTL or shCHD5 and the indicated CHD rescue constructs and analyzed at E18.5 (as in **Fig.6A**). Bar=25µm (**B**) Immunofluorescence analysis of E13.5 embryos *in utero* electroporated with either shCTL or shCHD3 with and the indicated rescue constructs and analyzed at E18.5 (as in **Fig.6E**). Bar=25µm (**C**) Quantification of pixel intensity of the experiments shown in (**A** and **B**). Average pixel intensity of overexpressed CHD proteins in GFP-expressing cells was normalised to average signal of the endogenous protein measured in neighbouring GFP-negative cells. 25-50 GFP positive and GFP negative cells analysed per embryo, n=3. Data are represented as mean ± SEM. * $p<0.05$, ** $p<0.01$, *** $p<0.001$; unpaired t-test (**D**) N2A cells were transfected with FLAG tagged forms of CHD3, CHD4 or CHD5 along with shRNAs against CHD3 and CHD5, as indicated. Co-immunoprecipitation was carried out using an anti-FLAG antibody, followed by western blotting for HDAC1, HDAC2, MTA2 and MBD3. Representative blot, n=3.

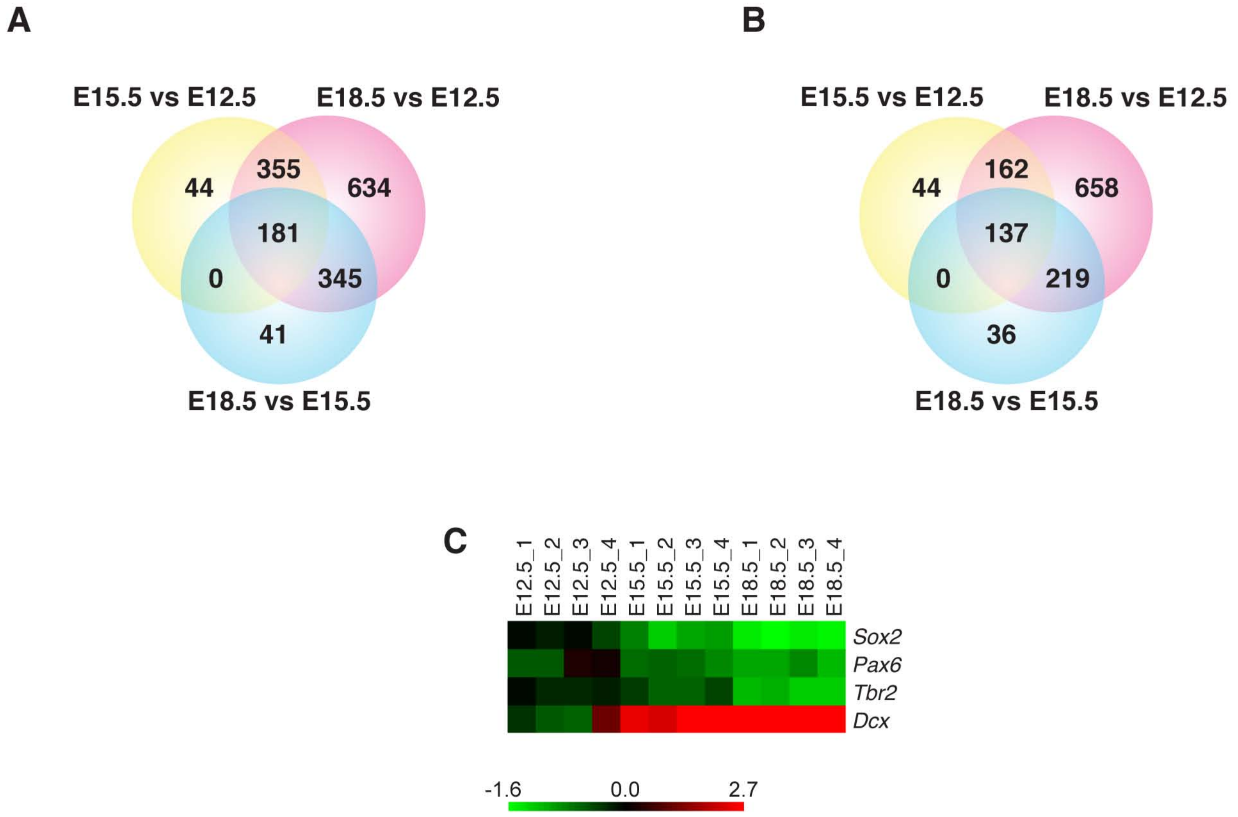

Figure S7. Related to Fig.1 and Fig.7 Microarray analysis of transcripts expressed during mouse cortical development (**A-B**) Venn diagrams of the 2035 genes upregulated (**A**) and 1613 genes downregulated (**B**) during cortical development. (**C**) Heatmap representing the log base 2 transformed expression levels of 4 putative NuRD target genes at the indicated embryonic stages. Colour scale bar represents log base 2 fold changes between each replicate and E12.5 group [2log(expression sample) – 2log(average expression E12.5 group)]; red, up-regulation; green, down-regulation; black, no change; n = 4.

SUPPLEMENTAL TABLES

Table S1. Proteins interacting with HDAC2 in the developing embryonic cortex. Related to Figure 1 and S1. The list of proteins identified as HDAC2 interactors in the E12.5, E15.5 and E18.5 embryonic mouse cortex. The list comprises NSAF values for proteins identified in the HDAC2 samples but absent from the normal IgG controls. Two biological repeats were analyzed for each time point. Components of the NuRD complex are highlighted in red.

Table S2. Differentially expressed genes in the developing mouse embryonic cortex. Related to Figure 7 and S6. The list of 2835 significantly differentially expressed genes identified by performing pairwise comparisons between each two developmental time points analyzed, (E15_Vs_E12, E18_Vs_E12 and E18_Vs_E15). FDR controlled p-value threshold of <0.01 together with a +/- 2 fold change was applied to assess significance, cortices from four independent embryos per each condition were analyzed.

SUPPLEMENTAL EXPERIMENTAL PROCEDURES

Co-immunoprecipitation

Co-immunoprecipitation experiments were performed using mouse embryonic cortices or rat cortical progenitor cultures. Cells were plated on 90mm dishes and after 5 days in culture, were washed with cold PBS and harvested in cold RIPA buffer (50mM Tris pH 7.5, 150mM NaCl, 1% NP40, 0.5% Deoxycholate) containing a protease inhibitor cocktail (Sigma). Cells or homogenized cortices were lysed in cold RIPA buffer containing the protease inhibitor cocktail for 30 minutes on ice, and lysates were sheared using 23G needle and cleared by centrifugation at 1000xg for 10 minutes at 4ºC. Protein concentration was determined using BCA Protein Assay (Thermo Scientific), according to the manufacture instructions and 0.5-1mg of protein was used for each co-immunoprecipitation. Lysates were pre-cleared with protein G-Sepharose (GE Healthcare Life Science) for 1 hour at 4^0C and incubated with the following antibodies: rabbit anti-CHD3 (5µg, Bethyl A301-220A), rabbit anti-CHD4 (2µg, Abcam ab72418), rabbit anti-CHD5 (5µL, generated by the Pazin Lab), rabbit IgG (Dako X 0903), mouse anti-HDAC2 (4µg, Abcam ab12169), mouse IgG (Santa Cruz sc2025), rabbit anti-MTA2 (5µg, Abcam ab8106) and mouse anti-FLAG (2.8µg, Sigma F31650). Lysates were rotated overnight at 4ºC and immune complexes were collected with protein G–Sepharose beads (2 hours at 4ºC). Beads were washed 4 times with washing buffer (50mM Tris pH 7.5, 150mM NaCl, 0.1% TX100, 5% glycerol) and two times with PBS. Proteins were eluted by boiling the beads with 2x Laemmli buffer (2% SDS, 20% glycerol, 2% 2-Mercaptoethanol, 0.006% bromophenol blue and 0.125 M Tris HCl, pH 6.8) and samples were analyzed by SDS-polyacrylamide gel electrophoresis (SDS-PAGE).

Cortical progenitor cultures

E14.5 rat cortices or E12.5 mouse cortices were isolated in dissociation buffer (2.5mM Hepes pH 7.4, 30mM glucose, 98mM Na_2SO_4, 1mM $CaCl_2$, 1mM $MgSO_4$, 4mM $NaHCO_3$, 1xHBSS) supplemented with 4mg/ml collagenase (Worthington, NJ) and 0.6mg/ml DNase (Sigma). Dissociated cortices were digested in dissociation media (1mM Hepes pH 7.4, 20mM glucose, 98mM Na_2SO_4, 30mM K_2SO_4, 5.8mM $MgCl_2$, 0.25mM $CaCl_2$, 0.001% Phenol red, 0.126mN NaOH) supplemented with 20U/ml of papain (Wortington) for 25min at 37^0C. After digestion, cortices were washed, dissociated and plated on Nunc dishes (Thermo Scientific) or glass coverslips coated with 40μg/ml poly-D-lysine (Sigma) and 2 μg/ml Laminin (BD Bioscience) in DMEM/F12 medium supplemented with 1x B27, 1x N2, 1mM glutamine and 1mM $NaHCO_3$. Plating medium was initially supplemented with 10ng/ml of bFGF (Life technologies). Cells were plated at $1.25*10^6$ cells per 90mm dish and $2.5*10^4$ cells on glass cover slips in 4 well plates. After 2 days *in vitro*, half of the medium was changed into Neurobasal medium with 1x B27, 1mM glutamine and supplemented with 100ng/ml NT3 (Alomone labs). NT3 was supplemented every 3 days. After 5 days cells were supplemented with 10μM 5-Fluoro-2′ -deoxyuridine (FdU). Cells were maintained in 37ºC, 5% CO_2 incubators for up to 7 days.

Western blotting

Tissues were homogenized using a hand-potter homogeniser (Sigma) in cold PBS. Cells were lysed in RIPA buffer containing a protease inhibitor cocktail (Sigma) on ice and sonicated for 5sec (Branson Sonifier 450). Proteins were measured using a BCA Protein Assay (Thermo Scientific). Prior to loading on gel, samples were boiled with 5 x Laemmli sample buffer (0.3125M Tris-HCl, pH 6.8, 5% SDS, 50% Glycerol, 5% 2-Mercaptoethanol and 0.015% bromphenol blue) for 5 min Samples were separated either on 8% polyacrylamide gels (Bio-Rad) or precast NuPAGE Novex 4-12% Bis-Tris gels (Life Technologies) and transferred onto PVDF membrane (GE Healthcare Life Science) using the Mini Trans-Blot tank transfer system (Bio-Rad) for 3h at 4ºC at constant 100V. Membranes were incubated with 5% milk in TBST (50mM Tris pH 7.6, 150mM NaCl, 0.1% Tween-20) for 1h at room temperature followed by primary antibodies diluted 1:1000 in 5% milk in TBST overnight at 4ºC. The primary antibodies used were goat anti-Hsp90 (Santa Cruz sc1055), rabbit anit-CHD3 (Abcam 109195), rabbit anti-CHD4 (Active Motif 39289), rabbit anti-CHD5 (gift from M. Pazin), rabbit anti-HDAC2 (Santa Cruz sc-7899), rabbit anti-HDAC1 (Abcam ab19845-100), mouse anti-MTA1 (Abcam ab50263), rabbit anti-MTA2 (Abcam ab8106), rabbit anti-MTA3 (Bethyl A300-160A), rabbit anti-p66 (Millipore 07-365), rabbit anti-RbAp46 (Abcam ab3535), rabbit anti-MBD3 (Abcam ab157464) and mouse anti-tubulin α (Sigma T9026). After three washes with TBST membranes were incubated with the appropriate secondary antibody for 2h at room temperature. Anti-mouse (GE Healthcare Life Science), anti-rabbit (GE Healthcare Life Science), anti-goat (Sigma) and anti-rat (Dako) secondary antibodies conjugated to horseradish peroxidase (HRP) were diluted 1:10000 in 5% milk in TBST. Signal was detected using ECL or ECL Prime detecting reagents (GE Healthcare Life Science) and by exposing the immunoblot to the Amersham Hyperfilm (GE Healthcare Life Science).

Plasmids

pCIG-NLSCRE plasmid was a gift from Francois Guillemot. pClneoB-3Flag-hCHD3 plasmid containing full sequence of human CHD3 was provided by Odd Stokke Gabrielsen. pCMV-SPORT6-mCHD4 plasmid containing complete coding sequence of mouse CHD4 was purchased from Open Biosystems. pDEST26-hCHD5 plasmid encoding full sequence of human CHD5 was purchased from Source Bioscience. Complete hCHD3, mCHD4 and hCHD5 coding sequences were subcloned into pCIG-IRES-GFP vector downstream of Flag tag using Gibson Assembly (NEB). In order to generate hCHD3 construct resistant to shCHD3 seven silent mutations were introduced into pCIG-hCHD3 plasmid using QuikChange Site-Directed Mutagenesis Kit (Agilent Technologies).

shRNA plasmids

For in utero electroporation experiments the oligo hairpins were ligated into pSUPER GFP plasmid (Oligoengine) using BglII/HindIII restriction sites. The shRNA sequences targeting mouse transcripts were as follows: shCHD5 5'-GATGCAAACATGTTTGTCTTG-3', shCHD3 5'-GCCAGGCCAACAAAGTGATG-3' and shCHD4 5'-AGTGAAAGACCCAGAGTGAT-3'. shCTL shRNA sequence 5'-GCGTACGGGGAAACTTCGA-3' was described previously (Egan, et al., 2014).

Tissue preparation

Embryonic brains were fixed using 4% paraformaldehyde (PFA) in PBS overnight at 4ºC. Fixed samples were cryoprotected using 30% sucrose overnight at 4ºC. Brains were frozen in Optimal Cutting Temperature (O.C.T, Sakura) and 10µm coronal sections were cut using a Leica cryostat.

Immunostaining

Cortical progenitor cultures were fixed with 4% PFA for 10min at RT. Fixed cells or tissue sections were permeabilised using 0.3% Triton X100 and 10% normal goat or donkey serum in PBS at room temperature for 1h and incubated with primary antibodies overnight at 4ºC. The following primary antibodies were used: rabbit anti-Ki67 (Abcam ab16667), chicken anti-GFP (Abcam ab13970), rat anti-Ctip2 (Abcam, ab18465), chicken anti-Tbr2 (Millipore AB15894), mouse anti-SATB2 (Abcam ab51502), rabbit anti-Cux1 (Santa Cruz sc13024), mouse anti-NeuN (Millipore MAB377), mouse anti-nestin (Santa Cruz sc33677), rabbit anti-MAP2 (Santa Cruz sc20172), rabbit anti-Pax6 (Covance PRB-278P), rabbit anti-Sox2 (Cell Signalling 27485), goat anti Brn2 (Santa Cruz sc-6029), rabbit anti Sox5 (Abcam ab94396), guinea pig anti Dcx (Millipore AB2253), mouse anto RhoA (Abcam ab54835), rabbit anit-CHD3 (Epitomics 2969-1), rabbit anti-CHD4 (Active Motif 39289), rabbit anti-CHD5 (gift from M. Pazin) and rat anti-CHD5 (gift from T. Tachibana). After three sequential washes with PBS, sections were incubated with Alexa Fluor conjugated secondary antibodies and 4',6-diamidino-2-phenylindole (Dapi) for 90 min at RT. Sections were washed with PBS and mounted using ProLong Gold (Life Technologies). Images were acquired using SP5 confocal microscope (Leica) with LAS AF software and processed using ImageJ software**.**

Cell cycle index analysis

Cell cycle exit was determined using EdU/Ki67 immunolabelling. For EdU incorporation timed pregnant females received an intraperitoneal injection of EdU (Invitrogen) at embryonic day 12 or 15. EdU was given at a dose of 20 mg/kg body weight in a solution of 10 mg/ml PBS. Embryonic brains were collected 24h after EdU injection and processed for immunostaining. Coronal sections were stained for EdU incorporation using the Click-iT EdU cocktail with AlexaFlour 555 (Invitrogen), immunolabelled with Ki67 antibody and counterstained with DAPI.

All imaging and image analysis of CHD4 null and control brains were performed blind. Images were acquired using SP5 confocal microscope with a 40X or 63X objective at 1024x1024 pixel resolution. Quantification of labeled cells within the cortical wall was performed by dividing the images into bins 200µm wide (E13.5) or 100µm wide (E16.5). The bins spanned the entire coronal section from the ventricular surface to the pia. $EdU^+/Ki67^-$ cells and total number of EdU^+ cells were counted per each bin. Cell cycle exit index was calculated as the percentage of $EdU^+/Ki67^-$ cells over the total number of EdU^+ cells.

TUNEL analysis

Fragmented DNA of apoptotic cells was labeled using the ApopTag Fluorescein Direct In Situ Apoptosis Detection assay (Millipore) following manufacturers protocol. All imaging and image analysis were performed blind. Images were acquired on SP5 confocal microscope with a 40X objective at 1024x1024 pixel resolution. A rectangular marquee with fixed width and spanning the entire cortex was used. The total number of apoptotic cells in the cortex was counted and divided by the area of the marquee to account for differences in the thickness of the cortex. Values were represented as the number of TUNEL+ cells per 1 μm^2 tissue area.

Radial neural migration analysis

Radial migration analysis of embryos electroporated *in utero* with the indicated GFP vectors was performed as described previously (Hand et al., 2005) using ImageJ and excel macro. Images were acquired on SP5 confocal microscope with a 20X objective at 1024x1024 pixel resolution. Confocal images were run through a Bandpass Filter to segment and isolate cell-sized shapes, thresholded and segmented into 10 radial regions between the ventricle and the pial surface. Individual cell position along the radial axis was recorded and imported into Excel along with the coordinates of top (pial) and bottom (ventricle) boundaries obtained using ImageJ's Path Writer plugin. Distance and percentage of migrating cells was calculated using an Excel macro.

Quantification of layer specific markers

For CHD4 null and control embryonic brains all imaging and image analysis were performed blind. Images were acquired on SP5 confocal microscope with a 40X objective at 1024x1024 pixel resolution. Confocal images of the coronal sections of the cortex were cropped into rectangle of 200μm (E13.5) or 100μm (E16.5 and E18.5) width extending from the ventricular surface to the pia. The total number of Pax6, Sox2, Tbr2, Ctip2, Tbr1, SATB2 and Cux1 positive cells within rectangle was counted for each embryo.

Measurements of pixel intensity

For CHD4 null and control cortical progenitors in culture all imaging and image analysis were performed blind. Cultures were obtained from 6 CHD4 null and 6 control E12.5 embryos from three independent litters. Images were acquired on a SP5 confocal microscope with a 63X objective at 1024x1024 pixel resolution. Three random fields were acquired per each cover slip. Approximately 300 cells from each embryo were quantified. Nuclei were highlighted using DAPI staining, than mean pixel intensity for each nuclear staining was measured in ImageJ.

For embryos *in utero* electroporated with shCTL, shCHD3 and shCHD5 all imagining and image analysis were performed blind. 25-53 cells from three independent embryos were quantified per each condition. Images were acquired on a SP5 confocal microscope with a 63X objective at 1024x1024 pixel resolution. Three coronal sections were analyzed per each embryo and four fields were acquired per each section. Electroporated cells were highlighted using GFP signal than mean pixel intensity was measured using ImageJ. Each measurement was normalized to the background signal in the nucleus of a neighboring GFP negative cell.

RNA extraction and qRT-PCR

Cortices were isolated and RNA extracted using RNeasy Mini Kit according to manufacturers protocol (Qiagen). Total RNA was eluted in 40μl DEPC water, and immediately DNase treated using TURBO DNase kit, according to manufacturers instructions (Ambion). Total RNA was reverse transcribed in a 20μl reaction volume containing random hexamer mix and Superscript III reverse transcriptase (50U, Invitrogen) at 50°C for 1h. As a control, reverse transcription was performed without the reverse transcriptase to confirm lack of genomic DNA contamination in the samples. Resulting cDNA was used for qPCR with the DyNAmo™ Flash qPCR Kit (Thermo Scientific). All reactions were performed in triplicate with a Mastercycler® ep realplex (Eppendorf) and each experiment included a standard curve. ΔΔCt method was used for relative quantification. Results were normalized to *rpl11* transcript. Primers used for qRT-PCR: *rpl11*, Fwd GCATCCGGAGAAATGAGAAA, Rev GAAATCCAGGCCATAGATGC; *CHD3*, Fwd CCACCTTCTCAACTTCCTCACC, Rev ACATCCGCCTTGAGTCTCCGAA; *CHD4*, Fwd GGACGACGATTTAGATGTAGAG Rew CCTGGTGGTCTGTCTCATAACC; *CHD5*, Fwd TGCAACCATCCGTACCTCTTCC, Rew TCAGCACTCTGTGCCCTTCATC.

Microarray analysis

RNA from E12.5, E15.5 and E18.5 cortices were isolated using RNeasy Mini Kit (Qiagen) according to manufacturers protocol. RNA quantity and quality was analyzed using Agilent 2100 Bioanalyser. RNA samples were amplified using the TotalPrep 96-RNA amplification kit from Ambion (Applied Biosystems). Whole-genome expression profiling of the samples was performed using mouseWG-6 v2.0 Expression BeadChip (Illumina) by Cambridge Genomic Services. Raw data were processed using the Bioconductor package lumi. Briefly, data were background corrected using the "forcePositive" method, log2 transformed and then quantile normalized. Fold changes in relative expression were calculated from the 2logfc (2^(2logfc)). Three pairwise comparisons E15_Vs_E12, E18_Vs_E12 and E18_Vs_E15 were performed. A 2 fold cut-off and FDR corrected p-value ≤ 0.01 was used to identify putative transcripts that were decreased (< 1/2) or increased (> 2) between comparisons.

To identify NuRD target genes, differentially regulated transcripts were compared with previously published CHD4 ChIP-seq data (Reynolds et al., 2012b, deposited in the ArrayExpress database accession number E-MTAB-888 and Hung et al., 2012 deposited in GEO database under accession number GSE30890). The comparisons were conducted using Galaxy tool (http:/fjfj /galaxyproject.org/). 12 candidate target genes were selected and validated by ChIP performed on lysates obtained from NPCs and PMNs. Heatmaps were generated with Matrix2Png (Pavlidis and Noble, 2003). Due to the poor annotation of the rat genome and the availability of mouse ChIP-seq data, mouse genome (mm9 and mm10 assembly) was used for reference when selecting genomic regions for ChIP experiments. Selected regions were classified as promoters based on 1) their distance from the TSS and 2) RNA polymerase II binding profile provided by ENCODE ChIP-seq datasets (Robertson et al., 2007). In addition Sox2, Pax6 and Tbr2 genomic regions were selected for targeted ChIP experiments based on published CHD4 ChIP-seq data (Reynolds et al., 2012b). An intergenic region 24kb downstream of *RhoA* TSS (*RhoA* neg) and a gene desert region of rat chromosome 1 (*Chr1*) were used as negative controls.

Chromatin immunoprecipitation

ChIP was performed as described previously (Hong et al., 2008) with minor modifications. Cells were crosslinked with 1% formaldehyde for 10 min at RT. Crosslinking was terminated by adding glycine to 125mM final concentration and incubation at RT for 5 min. Samples were washed once with PBS and homogenized in hand-potter homogeniser (Sigma) in PBS. Cells were collected by centrifugation and pellets were resuspended in buffer 1 (50mM Hepes-KOH, pH 7.5, 140 mM NaCl, 1 mM EDTA, pH 8.0, 10 % Glycerol, 0.5 % NP-40, 0.25 % Triton X-100) and lysed for 10 min at 4°C. Nuclei were pelleted and washed with buffer 2 (200 mM NaCl, 1 mM EDTA, pH 8.0, 0.5 mM EGTA, pH 8.0, 10 mM Tris-HCl, pH 8.0) and resuspended in buffer 3 (1 mM EDTA, pH 8.0, 0.5 mM EGTA, pH 8.0, 10 mM Tris-HCl, pH 8.0). All buffers contained protease inhibitor cocktail (Sigma P8340), phosphatase inhibitor cocktail (Sigma P2850 and P5726) and 1mM PMSF (Sigma). Samples were sonicated using a Bioruptor UCD-200 sonicator (Diagenode), to obtain fragments of 200bp-2kb. Cell debris was removed by centrifugation, and salt and detergent were added to the lysates to adjust buffer 3 composition to 1% Triton X-100, 0.1% DOC, 10mMTris-Cl, pH8.0, 150mM NaCl, 1mM EDTA pH 8.0, and 0.5mM EGTA, pH 8.0. Supernatants were pre-cleared by incubation with 70μl of Protein A–Sepharose beads (Amersham Biosciences) for 1 h at 4°C. 10% of the lysate was saved as total input control. 5μg of chromatin were used per each reaction and the volume of each sample was adjusted to 500μl with ChIP lysis buffer. 5–10μg of antibody was added and samples were rotated overnight at 4°C. The following antibodies were used: rabbit IgG (Dako X0903), mouse IgG (Santa Cruz sc2025), rabbit CHD3 (Bethyl A301-220A), mouse CHD4 (Abcam ab70469), rabbit CHD5 (gift from M. Pazin), goat Sox2 (R&D AF2018). Immune complexes were collected by incubation with 80μl of Protein A–Sepharose beads for 2h at 4°C. Beads were collected and subjected to a series of seven sequential washes, 2 x low salt buffer (0.1% SDS, 0.5% Triton X100, 2mM EDTA, 20mM Tris-HCl pH 8.1, 150mM NaCl), 2 x high salt buffer (0.1% SDS, 1% Triton X100, 2mM EDTA, 20mM Tris-HCl pH 8.1, 500mM NaCl), 2 x LiCl buffer (10 mM Tris-HCl pH 8.1, 1 mM EDTA, 0.25 M LiCl, 1% DOC, 1% NP40) and 2 x TE (10 mM Tris-HCl pH 8.1, 1 mM EDTA) for 10min each. After a final TE wash, the supernatant was entirely removed and beads were eluted in 150μl of freshly prepared elution buffer (0.1M $NaHCO_3$ pH 8.0, 1% SDS) and vortexed for 15 min at RT. Crosslinking was reversed by adding 10μl 5M NaCl to the samples and incubating them at 65^0C over night. DNA fragments from ChIP samples and total input were purified using the QIAquick PCR purification kit (Qiagen) according to the manufacturer's protocol and eluted twice in 40μl of EB buffer. Purified DNA was subjected to qPCR analysis. qPCR was performed using the DyNAmo™ Flash qPCR Kit (Thermo Scientific). All values were calculated as a percentage of total input. Primers are available upon request.

SUPPLEMENTAL REFERENCES

Hand, R., Bortone, D., Mattar, P., Nguyen, L., Heng, J.I.-T., Guerrier, S., Boutt, E., Peters, E., Barnes, A.P., Parras, C., *et al*. (2005). Phosphorylation of Neurogenin2 Specifies the Migration Properties and the Dendritic Morphology of Pyramidal Neurons in the Neocortex. Neuron *48*, 45-62.

Hong, E.J., McCord, A.E., and Greenberg, M.E. (2008). A Biological Function for the Neuronal Activity-Dependent Component of Bdnf Transcription in the Development of Cortical Inhibition. Neuron *60*, 610-624.

Pavlidis, P., and Noble, W.S. (2003). Matrix2png: a utility for visualizing matrix data. Bioinformatics *19*, 295-296.

Robertson, G., Hirst, M., Bainbridge, M., Bilenky, M., Zhao, Y., Zeng, T., Euskirchen, G., Bernier, B., Varhol, R., Delaney, A., *et al*. (2007). Genome-wide profiles of STAT1 DNA association using chromatin immunoprecipitation and massively parallel sequencing. Nat Meth *4*, 651-657.
